# Analysis of an Ordered, Comprehensive STM Mutant Library in Infectious *Borrelia burgdorferi*: Insights into the Genes Required for Mouse Infectivity

**DOI:** 10.1371/journal.pone.0047532

**Published:** 2012-10-25

**Authors:** Tao Lin, Lihui Gao, Chuhua Zhang, Evelyn Odeh, Mary B. Jacobs, Loïc Coutte, George Chaconas, Mario T. Philipp, Steven J. Norris

**Affiliations:** 1 Department of Pathology and Laboratory Medicine, Medical School, University of Texas Health Science Center at Houston, Houston, Texas, United States of America; 2 Department of Microbiology and Molecular Genetics, Medical School, University of Texas Health Science Center at Houston, Houston, Texas, United States of America; 3 Department of Parasitology, Tulane Regional Primate Research Center, Tulane University Health Sciences Center, Covington, Louisiana, United States of America; 4 Department of Biochemistry and Molecular Biology and Department of Microbiology and Infectious Diseases, The University of Calgary, Calgary, Alberta, Canada; University of Kentucky College of Medicine, United States of America

## Abstract

The identification of genes important in the pathogenesis of Lyme disease *Borrelia* has been hampered by exceedingly low transformation rates in low-passage, infectious organisms. Using the infectious, moderately transformable *B. burgdorferi* derivative 5A18NP1 and signature-tagged versions of the *Himar1* transposon vector pGKT, we have constructed a defined transposon library for the efficient genome-wide investigation of genes required for wild-type pathogenesis, in vitro growth, physiology, morphology, and plasmid replication. To facilitate analysis, the insertion sites of 4,479 transposon mutants were determined by sequencing. The transposon insertions were widely distributed across the entire *B. burgdorferi* genome, with an average of 2.68 unique insertion sites per kb DNA. The 10 linear plasmids and 9 circular plasmids had insertions in 33 to 100 percent of their predicted genes. In contrast, only 35% of genes in the 910 kb linear chromosome had incapacitating insertions; therefore, the remaining 601 chromosomal genes may represent essential gene candidates. In initial signature-tagged mutagenesis (STM) analyses, 434 mutants were examined at multiple tissue sites for infectivity in mice using a semi-quantitative, Luminex-based DNA detection method. Examples of genes found to be important in mouse infectivity included those involved in motility, chemotaxis, the phosphoenolpyruvate phosphotransferase system, and other transporters, as well as putative plasmid maintenance genes. Availability of this ordered STM library and a high-throughput screening method is expected to lead to efficient assessment of the roles of *B. burgdorferi* genes in the infectious cycle and pathogenesis of Lyme disease.

## Introduction

Lyme borreliosis is the most common tick-borne disease in North America and Eurasia. It is a multi-stage, systemic infection caused by the spirochete *Borrelia burgdorferi* sensu stricto in North America, and by *B. garinii*, *B. afzelii*, and *B. burgdorferi* in Eurasia [1], [2]. Lyme disease borreliae are maintained in nature via transmission cycles between reservoir hosts and tick vectors. Spirochetes are transmitted to a variety of small mammals, some species of birds, and humans via the bite of infected ticks of the *Ixodes ricinus* complex, including *Ixodes scapularis* in the Northeastern, North Central, middle Atlantic, and Southeastern U.S., *Ixodes pacificus* in Northern California, and *Ixodes ricinus* and *Ixodes persulcatus* in Eurasia [3]. The spirochetes disseminate throughout the mammalian host. Acute and chronic infections give rise to inflammation and morbidity in multiple organs and tissues, including the skin, joints, heart, and nervous system [1].

The complete genome sequence of *B. burgdorferi* B31 includes a 910 kb linear chromosome and 12 linear and 9 circular plasmids that total 610 kb [4], [5]. However, no toxins and only a few potential virulence determinants could be inferred by sequence homology. Despite recent advances in genetic analysis of Lyme disease *Borrelia* [6], site-directed mutagenesis and complementation using low-passage, infectious *B. burgdorferi* has been a challenging approach due to exceedingly low transformation rates, a 6–8 hour doubling time, and spontaneous plasmid loss during *in vitro* culture. To date, fewer than 50 genes have been disrupted by site-directed mutagenesis in infectious *B. burgdorferi* strains [6], [7].

Several plasmid-encoded products have been shown to be required for survival or infectivity of *B. burgdorferi* strain B31 [8]–[14]. These include the cp26-encoded proteins ResT (telomere resolvase, required for linear chromosome and plasmid replication) [15], [16], OspC (outer surface protein C, required for early mammalian infection) [17]–[21], and BBB26/BBB27 (homologous proteins with an unknown function essential for *in vitro* survival that cross-complement one another) [15], [16]. lp25 encodes PncA, a nicotinamidase required during mammalian infection and involved in the conversion of nicotinamide to NAD [9]. lp36 contains the gene for AdeC, an adenine deaminase also required for infection of mice [14]. Approximately 10 kb of lp28-1 is devoted to the VMP-like sequence (*vls*) antigenic variation system, which is involved in immune evasion and long-term survival of Lyme disease *Borrelia* in mammals [22], [23]. Finally, OspA and OspB, encoded on lp54, interact with a tick epithelial protein called TROSPA and are required for tick midgut colonization [24]–[28]. The presence of the linear plasmids lp25 and lp56 correlates with the low electroporation efficiency of low-passage, infectious clones and has been attributed to two large open reading frames (*bbe02* and *bbq67*, respectively) that have sequence similarity to Type IV restriction endonucleases with modification activity [29]. Kawabata et al. [30] constructed the infectious but moderately transformable *Borrelia burgdorferi* B31 clone 5A18NP1 by selecting a clone in which lp56 had been lost and inactivating *bbe02* on lp25.

Transposons are DNA elements with the ability to move or transpose from one location to another in a genome [31]. The ‘cut-and-paste’ transposons in the *mariner* family have been widely utilized for random inactivation of genes because of their insertion at common 5′-TA-3′ sequences with little additional sequence bias and the lack of requirement for host-derived factors [31]–[33]. *Himar1* transposase mutants such as the C9 derivative developed by Lampe et al. [34] have been particularly useful because of their heightened transposition frequency, and have been employed to generate transposon mutant libraries in a broad range of prokaryotes. Signature-tagged mutagenesis (STM) is a powerful negative selection method that has been widely used to identify bacterial virulence factors required for the successful adhesion, colonization, dissemination, persistence, and survival in the host [35]–[37]. In STM, clones are identified by unique DNA sequences incorporated into the transposable element, permitting co-infection of animal models with multiple transposon mutants, each with a different STM ‘tag’. Sequencing of the transposon insertion sites creates a defined library, allowing the targeted analysis of mutations in genes of interest. The STM approach has been used to analyze more than 31 bacterial species, resulting in the identification of over 1700 virulence determinant gene candidates [35]–[37].

Mariner transposition was adapted successfully to provide random mutagenesis in non-infectious *B. burgdorferi* using the suicide *Himar1* delivery vector pMarGent [38]; subsequently, a small library of 33 insertion mutants was created by using the same vector in the infectious, moderately transformable strain 5A18NP1 [39]. However, examination of the infectivity of a large number of mutants individually is impractical, and represents a major limiting step. To facilitate the systematic identification of virulence determinants required for *B. burgdorferi* infection *in vivo* in mammalian hosts and tick vectors, essential genes for *in vitro* growth, genes required for plasmid replication and maintenance, and biological roles of genes of unknown functions, we created and assembled a comprehensive library of sequence-defined STM mutants of infectious *B. burgdorferi*. The defined genome-scale library provides a convenient resource for analysis of nonessential genes of interest. Currently, the insertion sites of 4,479 unique mutants have been determined and 434 STM mutants have been screened for infectivity in mice using a novel Luminex-based technology, leading to the identification of many virulence determinant candidates.

## Results

### Assembly of a Sequence-defined, High Density Signature-tagged Mutagenesis Library

**Figure 1:**
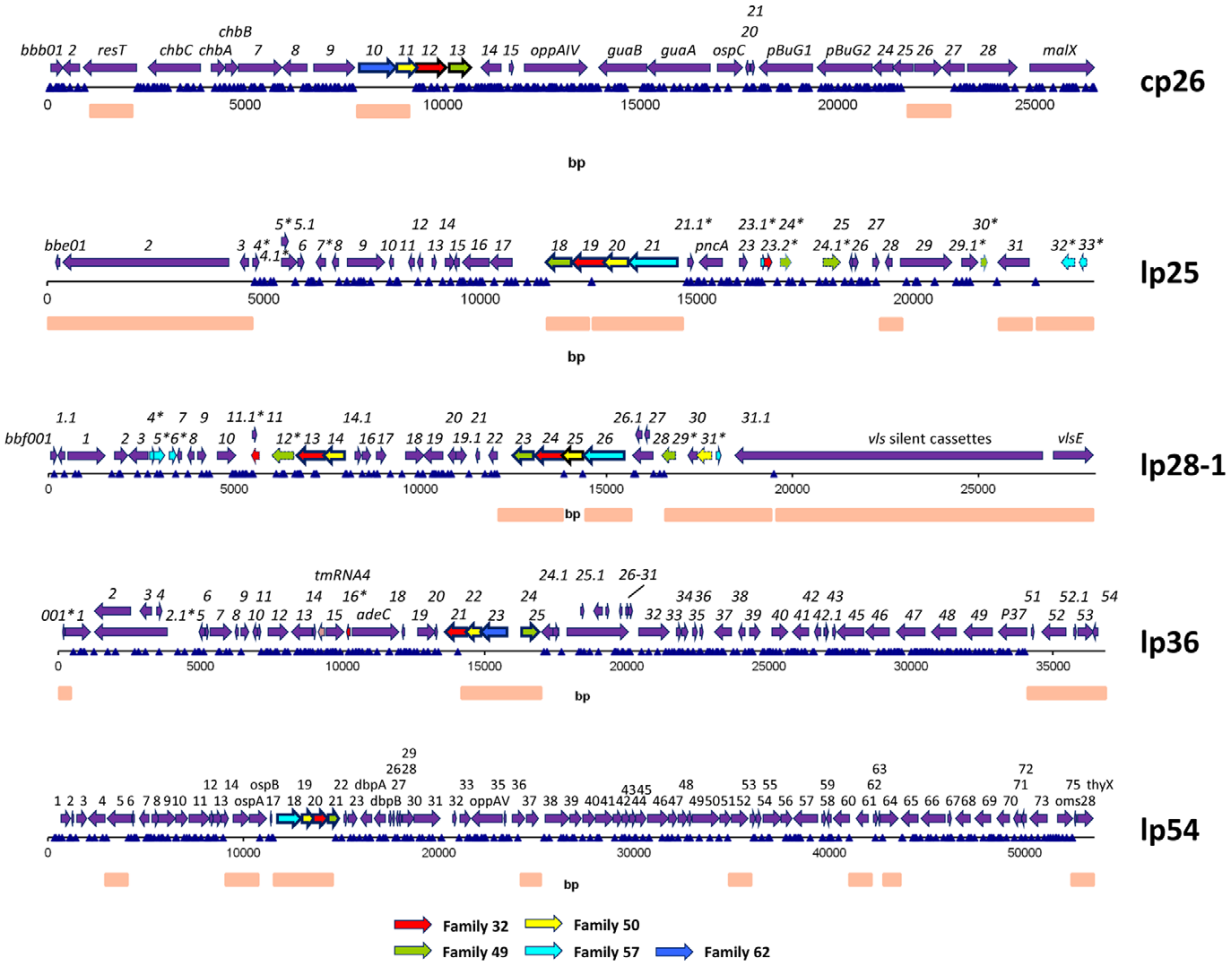
Maps of the STM transposon insertion points in representative plasmids of *B. burgdorferi* B31 5A18NP1. The following plasmids are shown: cp26 (Plasmid B, 26,498 bp, 307 transposon mutants, 250 unique sites), lp25 (Plasmid E, 24,177 bp, 102 transposon mutants, 85 unique sites), lp28-1 (Plasmid F, 26,921 bp, 83 transposon mutants, 71 unique sites), lp36 (Plasmid K, 29,766 bp, 224 transposon mutants, 209 unique sites), and lp54 (Plasmid A, 223 transposon mutants, 195 unique sites). Transposon insertion sites are depicted as blue triangles. ORFs that represent apparent pseudogenes or gene fragments are indicated by asterisks after the gene number. Regions that lack transposon insertions are indicated by orange rectangles below each map. A key for the color coding of paralogous gene families commonly associated with the origins of replication of *B. burgdorferi* plasmids is provided at the bottom of the figure. Maps of the transposon insertion sites for all of the plasmids are provided in Supporting Information, Fig. 3 in File S1.

The *Himar1* transposon vector pGKT [40] was used as the basis for the construction of suicide *Himar1* transposon vectors for use in *B. burgdorferi*. The vectors pGKTSTM1 through pGKTSTM11 were constructed by inserting eleven 7-bp tags into a region between the ColE1 origin and inverted terminal repeat 2 (ITR2) in plasmid pGKT (Fig. 1 in File S1). The infectious, moderately transformable *B. burgdorferi* strain 5A18NP1 was transformed with these plasmids to create an STM library in an infectious background. Between 100 and 300 clones were obtained with each transformation of 5×10^9^ organisms with 10 µg DNA, yielding an electroporation frequency of 2 x10^−8^ to 6×10^−8^. A library of 6,625 STM mutants has been created, and the insertion sites have been determined for 4,479 of these mutants (Table 1; Spreadsheet S1).

**Table 1:** Distribution of transposon insertions among the *B. burgdorferi* replicons.

Replicon	Total BP perReplicon	Total Number of Insertions Mapped	Total InsertionSites perkb DNA	Number of Unique Insertion Sites	Unique Insertion Sites per kb DNA
Chromosome	910,724	1,486	1.63	1,284	1.41
cp9	9,386	225	23.97	169	18.01
cp26	26,498	372	14.04	302	11.40
cp32-1	30,750	28	0.91	26	0.85
cp32-3	30,223	73	2.41	70	2.32
cp32-4	30,299	169	5.58	149	4.92
cp32-6	29,838	163	5.46	148	4.96
cp32-7	30,800	116	3.77	110	3.57
cp32-8	30,885	244	7.90	201	6.51
cp32-9	30,651	128	4.18	114	3.72
lp5	5,228	14	2.68	14	2.68
lp17	16,823	156	9.27	124	7.37
lp21	18,753	107	5.71	100	5.33
lp25	24,177	115	4.76	96	3.97
lp28-1	26,921	97	3.60	82	3.05
lp28-2	29,766	104	3.49	96	3.23
lp28-3	28,601	160	5.59	134	4.69
lp36	36,849	285	7.73	266	7.22
lp38	38,829	214	5.51	185	4.76
lp54	53,561	223	4.16	195	3.64
lp28-4	27,323	–	–	–	–
lp56	52,971	–	–	–	–
Total	1,439,572	4,479	3.11	3,865	2.68

Total genome values exclude lp28-4 and lp56, which are absent in the parental strain used in this study (B31 5A18NP1).

Mapping of the transposon insertion sites permitted the assessment of their distribution. Insertion sites were distributed on all 20 of the linear and circular replicons present in the parent strain, 5A18NP1 (Table 1, Spreadsheet S1). However, the number of insertions per kb of DNA varied widely from 0.91 in the circular plasmid cp32-1 to 23.97 in cp9; the average value for the genome was 3.11. The chromosome had a relatively low value of 1.63 insertions per kb DNA, consistent with a high proportion of essential genes in that replicon. The library included 3,865 unique insertion sites, *i.e.* 614 clones had insertion sites identical to those of other transposon mutants. These included 124 sibling clones (isolated from the same transformation); most of the siblings were obtained in early transformations which utilized longer incubation periods (*e.g.* 48 h) in liquid medium prior to plating. The remaining 490 clones with shared insertion sites (n  = 230) were independently derived from different transformations. No indication of sequence bias beyond the recognition of the well-characterized 5′-TA-3′ insertion site was observed. Anomalous (non-TA) insertion points were present in 55 (1.2%) of the clones, with sequences of TT and AT (12 clones each), AA (9), GA, TC, and TG (5 each), AG, CT, and GT (2 each), and AC (1).

**Figure 2:**
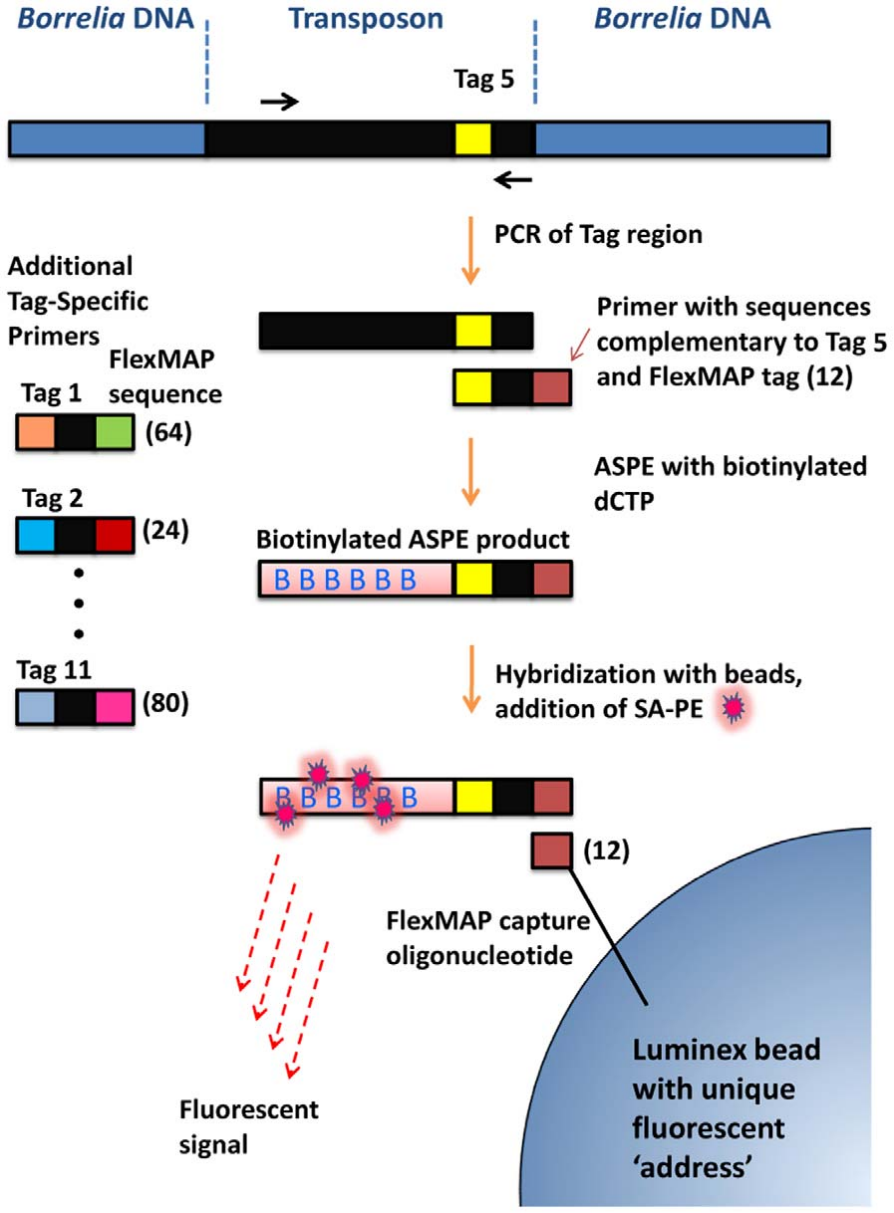
Luminex-based procedure for detection of STM clones in tissues and in cultures. A region of the transposon containing the signature tags was first amplified using PCR. The product was treated with Exonuclease I and shrimp alkaline phosphatase to remove unused primers and nucleotides, respectively. An asymmetric primer extension (ASPE) step was then used to simultaneously add a Luminex FlexMAP sequence and label the product with biotinylated dCTP. Streptavidin-phycoerythrin was added to provide the fluorescent signal, and the products were bound to Luminex beads with specific Flexmap capture oligonucleotides and unique fluorescent addresses. Distinct sets of ASPE primers (left side) and corresponding FlexMAP beads were included in the multiplex reaction to specifically detect the presence of each STM clone.

Analysis was further extended to the number of genes with transposon insertions (Table 2). Overall, 790 (45.5%) of the predicted protein-encoding genes in the genome were disrupted in the STM transposon library. This value was approaching saturation toward the end of the transposon mutant selection process (Fig. 2A in File S1); for example, in the final transformation only 11 of 260 clones examined (4.2%) had insertions in genes that had not been disrupted previously. As expected, a lower proportion of genes were disrupted in the chromosome, with only 287 of 857 (34%) of predicted protein-encoding genes having insertions. In contrast, over 90% of the genes were disrupted in cp9, cp26, and cp32-6, indicating a small number of the encoded products of these plasmids are needed for *in vitro* growth of *B. burgdorferi* or plasmid maintenance. Each of the two 23S rRNA-encoding loci (22 mutants total) and one of two 5S rRNA genes (1 mutant) had insertions, but the single 16S rRNA locus and the many tRNA loci were not disrupted. A much lower proportion of chromosome intergenic regions had insertions than those in the plasmids (14% as compared to 38%), perhaps related to the smaller average size of chromosomal intergenic regions and the higher density of promoters and other regulatory elements that could affect borrelial survival and growth. Several of the clones with transposon insertions in cp32 plasmids could not be assigned unambiguously to a single plasmid; in these cases, the sequence obtained from circularized genome fragments containing the transposon (typically ∼500 bp) was identical to homologous regions of two or more plasmids in the highly conserved cp32 sequences. For those genes with insertions, there was a median of 4.7 insertions per kb DNA and 6.4 insertions per gene; ten genes had more than 30 transposon insertions. There were no overall differences in the number of insertions per kb DNA in the genes of the chromosome, linear plasmids, or circular plasmids (Fig. 2B in File S1).

**Table 2:** Proportion of predicted protein genes and intergenic regions with transposon insertions in the *B. burgdorferi* replicons.

Replicon	Number of ORFS	Number of BPin ORFs	Average BPper ORF	Median BPper ORF	Number of ORFs with Insertions	Percent of ORFs with Insertions
Chromosome	857	856,064	999	858	287	34
cp9	11	7,125	648	558	10	91
cp26	29	23,355	805	633	26	90
cp32-1	42	28,464	678	577.5	12	29
cp32-3	45	28,077	624	567	26	58
cp32-4	45	28,326	629	567	35	78
cp32-6	42	27,783	662	592.5	38	90
cp32-7	44	28,941	658	571.5	34	77
cp32-8	43	28,686	667	573	34	79
cp32-9	43	28,468	662	567	32	74
lp5	7	3,850	550	432	2	29
lp17	29	11,351	391	270	20	69
lp21 (w/o repeat region)	12	6,178	515	441.5	4	33
lp25	39	16,375	420	186	20	51
lp28-1 (w/o vls region)	37	13,597	367	285	23	62
lp28-2	34	27,619	812	765	27	79
lp28-3	48	20,340	424	228	26	54
lp36	60	30,571	510	288	40	67
lp38	59	28,233	479	285	35	59
lp54	76	44,649	587	564	59	78
lp28-4	47	18,219	388	219	–	–
lp56	90	48,741	542	480	–	–
Total	1739	1,355,012	779	637.5	790	46

	Number of Intergenic Regions (IRs)	Number of BPin IRs	Average BPper IR	Median BPper IR	Number of IRswith insertions	Percent of IRswith insertions
All Intergenic Regions	1322	161,306	122	58	310	25
Chromosomal IRs	644	57,746	90	39	91	14
Plasmid IRs	678	103,560	153	78.5	219	38

We used the insertion ratio (distance in bp from the insertion site to the 5′ end of the gene divided by the total gene length) to provide a convenient measure of the location of the transposon insertion within the gene. The insertion ratio was also determined for intergenic regions, but in this case was simply oriented relative to the 5′ end of the replicon. There were 234 genes with only a single transposon disruption: 87 in the chromosome and 147 in the plasmids. Plotting the percentage of insertions at different insertion ratios (Fig. 2C in File S1) indicated that insertions in the last 10% of the gene length were recovered at a higher rate (33.33% of the chromosomal genes and 15.65% of the plasmid genes). This result is consistent with improved preservation of functional gene products when insertions occur late in the gene [41]. Examples of probable essential genes in which single insertions occurred in the final 10% of the reading frame include those encoding the cell division protein FtsH, replicative DNA helicase DnaB, ribosomal protein S21, glucose-6-phosphate isomerase (Pgi), a C-terminal protease (Ctp), two pantothenate transport and utilization proteins (PanF and Dfp), two enzymes involved in peptidoglycan synthesis (MurD and MurG), and the cp26-encoded telomere resolvase ResT. Other genes with only late insertions may also be essential (Tables 1 and 2 in File S1).

There was also a subset of genes that had an unexpectedly high number of transposon insertions, including 105 genes >100 bp in length that had more than 10 insertions per kb DNA. This set comprises the genes for histidine kinase 1 (*bb420*; 49 unique insertion sites, 10.93 per kb DNA), the cp26-encoded oligopeptide transporter periplasmic binding protein OppAIV (*bbb16*, 29 insertions, 18.20 per kb DNA), conserved integral membrane protein *bb0017* (26 insertions, 27.08 per kb DNA), and cp9-encoded conserved hypothetical protein *bbc12* (31 insertions, 23.98 per kb DNA).

The ordered transposon library described here is not saturating in most replicons, so essential genes cannot be identified simply as those without insertions. However, functional groups of genes that are either essential or nonessential for 1) *in vitro* growth in BSK-II medium or 2) plasmid replication can be discerned (Table 3; Table 1 in File S1). The functional groups that were found to be nonessential for *in vitro* growth included some regulatory factors, genes of the phosphoenolpyruvate phosphotransferase system (PEP-PTS), additional transport systems, genes involved in glycerol transport and incorporation, DNA repair and recombination genes, chemotaxis and motility genes, and several known virulence determinants and candidates. As expected, only a few genes involved in DNA replication, transcription and translation, peptidoglycan biosynthesis and intermediate metabolic pathways had transposon insertions, and these insertions were commonly at the 3′ ends of the genes or were in genes that encoded accessory functions. A detailed listing of the genes with assigned functions and their transposon disruption patterns is provided in Table 1 in File S1.

**Table 3:** Summary of essential gene group candidates, and apparent infectivity phenotypes of nonessential genes (based on STM results).

**Essential gene candidates (not disrupted in transposon library)**
DNA replication, transcription, translation
Glycolysis and pentose phosphate pathways
Fatty acid and phospholipid interconversion
V-type ATPase, thioredoxin, thioredoxin reductase
Peptidoglycan synthesis, protein translocation (*e.g.* Sec)
Core chaperones (GroELS, HP70)
Cofactor interconversion (*e.g.* pantothenate)
**Nonessential gene candidates (disrupted in transposon library)**
** Candidate virulence determinants (required for full infectivity in mice)**
Chemotaxis (9/13 genes tested required), motility
Nucleoside interconversion enzymes (GuaA, GuaB*, AdeC); nicotinamidase PncA
PF32, PF49 plasmid maintenance proteins
Chaperones HtpG, DnaJ-2, DnaK-1*
Regulators RpoN, RpoS, SpoT, arginine deiminase ArcA
Outer surface proteins: OspC
Adhesins: decorin binding protein DbpA, GAG binding protein Bgp/Pfs-2, RevA protein BBM27
Transport proteins:
ABC systems: MglA, BB0573, BBJ26, ChbB, ProX, ProW
PEP-PTS system: PtsG, FruA-1, FruA-2*, MalX-1*, MalX-2, ChbB
Glycerol uptake facilitator (GlpF)
Other: GltP, LctP, NhaC 1,2, OppA 1,2,3*,4,5*, BesA, BesC
DNA recombination and repair proteins: RuvAB, MutS, MutS-II, NucA, RnhB
Protein, DNA, RNA, polysaccharide degradation: Ung, PepX
**Not required for mouse infectivity**
DNA recombination and repair (except RuvAB, MutS, MutS-II, NucA, RnhB)
Most lipoproteins, protein antigens (*e.g.* P35, P37)
Chaperone BB0602
Outer surface proteins: OspB, ErpG,K,L,Y, OspD, Tpn50 (BB0167)
Adhesins: DbpB, BBK32
CRASP1, CRASP2 (BBH06)†, Arp (BBF01)
Transport proteins:
PEP-PTS system: PtsH-1, ChbA, ChbC
Other: BesB
Phosphoglycolate phosphatase Gph
Transposon-like proteins
Exonuclease SbcC

* Gene disruptions that gave rise to an intermediate infectivity phenotype.

† Equivocal; disparate results for different clones or experiments.

**Figure 3:**
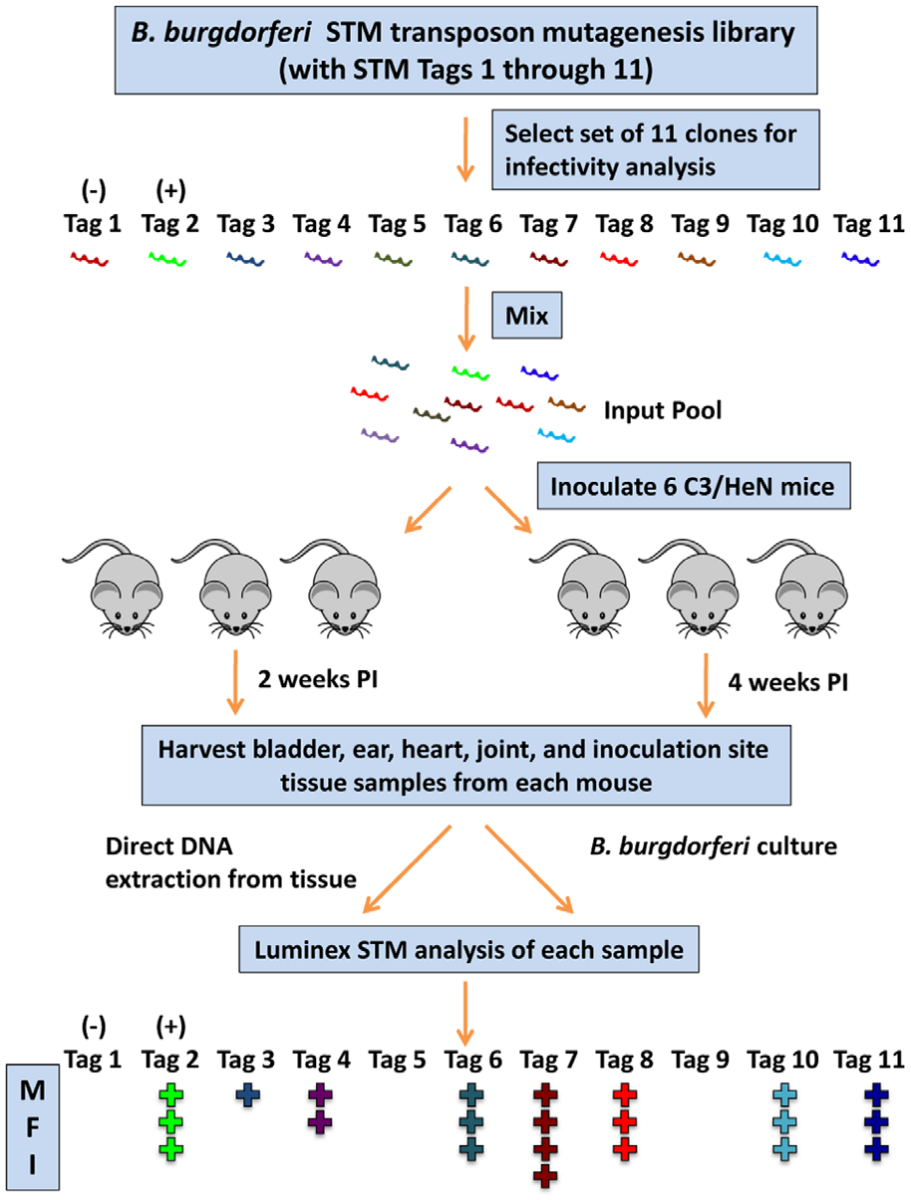
Procedure for high-throughput STM infectivity analysis of *B. burgdorferi* transposon mutants in this study. A mixture of 11 STM clones, each with a different signature tag, were inoculated (10^5^/clone i.d.) into two groups of three C3H/HeN mice. Two and four weeks post inoculation (PI), a group of mice were sacrificed and the five tissues indicated were collected. A portion of each tissue was used for direct DNA extraction, whereas another portion was cultured *in vitro*. Each of the 60 samples was then analyzed using the Luminex STM procedure. The median fluorescence intensity (MFI) obtained for 100 beads for each tag provided a measure of the concentration of each STM-tagged organism in the clone, and results were compared to those obtained with the input pool. Tag 1 and Tag 2 clones represent infectivity-negative (*pncA^−^*) and -positive (*BB0051^-^*) control transposon mutants, respectively.

Some of the plasmids had a high number of insertions per kb of DNA, so the distribution of insertions in these cases may more reliably identify candidate essential genes. The insertion maps of plasmids of interest are shown in Fig. 1; the maps for all plasmids are provided in the Supporting Information (Fig. 3 in File S1). The circular plasmid cp26 will be used as an example of the identification of candidate essential genes here. Of the 29 annotated genes in cp26, 26 genes (90%) were disrupted in the transposon mutagenesis library (Fig. 1). No insertions were observed in 3 ORFs: *bbb10*, *bbb11*, and *bbb26*. BBB10 and BBB11 are predicted proteins belonging to the Paralogous Families (PFs) 62 and 50, and are likely involved in plasmid replication or partitioning. *bbb10, bbb11*, and *bbb13* were shown recently to support the maintenance of a partition-defective MiniF plasmid in a heterologous *E. coli* plasmid system [42]. Jewett et al. [15] determined that BBB26 and BBB27 are highly homologous, membrane-associated periplasmic proteins, and that one or the other gene had to be intact to promote bacterial viability. In our mapping of transposon insertion sites in cp26, *bbb27* had three independent insertions (two at the same location), whereas no insertions were identified in *bbb26*. In the STM mutant T08TC298, the gene encoding the telomere resolvase ResT (*bbb03*) was disrupted at the 3′ end (insertion ratio  = 0.94). ResT is critical for the replication of the linear chromosome and linear plasmids [43], [44]. We did not notice an obvious *in vitro* growth defect in T08TC298; indeed, this *resT* mutant was both viable and infectious. It is likely that T08TC298 expresses a truncated but functional ResT protein.

### Plasmid Profiles of STM Transposon Mutants

A total of 4464 STM mutants have been examined for plasmid content to date [45]. In this PCR-based Luminex analysis, the fluorescence output obtained for each plasmid was evaluated as positive, intermediate, or negative (Spreadsheet S1) [45]. The intermediate category was included because the median fluorescent intensity (MFI) values obtained for some plasmids could not be clearly subdivided into positive and negative groups; the clones that have intermediate MFI values may contain a mixture of cells that have retained or lost the plasmid in question. For the 4464 STM clones examined, 1799 (40.3%) retained all plasmids. Plasmid loss occurred most commonly with lp5 (1916 clones, 42.9%), cp9 (602, 13.5%), lp21 (301, 6.7%), lp28-1 (291, 6.5%), and cp32-6 (135, 3.0%). One hundred three mutants (1.6%) had negative or intermediate plasmid analysis results for lp36. Only one clone had lost lp25 due to consistent inclusion of both kanamycin and gentamycin in the culture medium for the transposon mutants, resulting in selection for the kanamycin resistance cassette present in lp25 of the parent clone 5A18NP1; the one lp25-negative clone may be a spontaneous kan^R^ mutant. Loss of other plasmids occurred in less than 2% of examined STM mutants; cp26 and lp54 were consistently present, and cp32-1, cp32-8, cp32-9, and lp28-3 were absent from 0.3% or less of the clones. Prior studies have shown that lp25, lp28-1, and lp36 are required for full infectivity of *B. burgdorferi*, so clones lacking these plasmids were excluded from infectivity analysis. lp5 and lp21 are not essential for infection of mice [8], [46]. The potential role of cp32-6 (deficient in 3% of transposon mutants) in *B. burgdorferi* infectivity of mice or ticks is not known.

### Screening of STM Mutants for Infectivity in Mice

Initial efforts to screen the STM mutants for infectivity utilized PCR and gel electrophoresis (data not shown). However, examination of a large number of mutants from input and output pools using this method is time-consuming and laborious, and generally provides only a subjective measure of infectivity. To overcome this barrier and facilitate the high-throughput detection of *B. burgdorferi* STM mutants in mammalian hosts, we developed a Luminex-based multiplex PCR procedure for semi-quantitative detection of STM mutants (Fig. 2). The Luminex protocol was performed in parallel using DNA extracted directly from the mouse tissue, and DNA extracted from *B. burgdorferi* cultured from the same tissue specimen. Because each tissue specimen required only one well in a 96-well plate, it was also possible to evaluate multiple tissue sites (bladder, ear, heart, tibiotarsal joint, and inoculation site) and sets of three mice per time point. In this manner, we were able to assess the occurrence of localized and disseminated infection at 2 and 4 weeks post inoculation in five tissues from 3 mice with two DNA preparation methods (Fig. 3). Thus, in a typical analysis, there were 60 MFI values collected for each clone inoculated.

**Figure 4:**
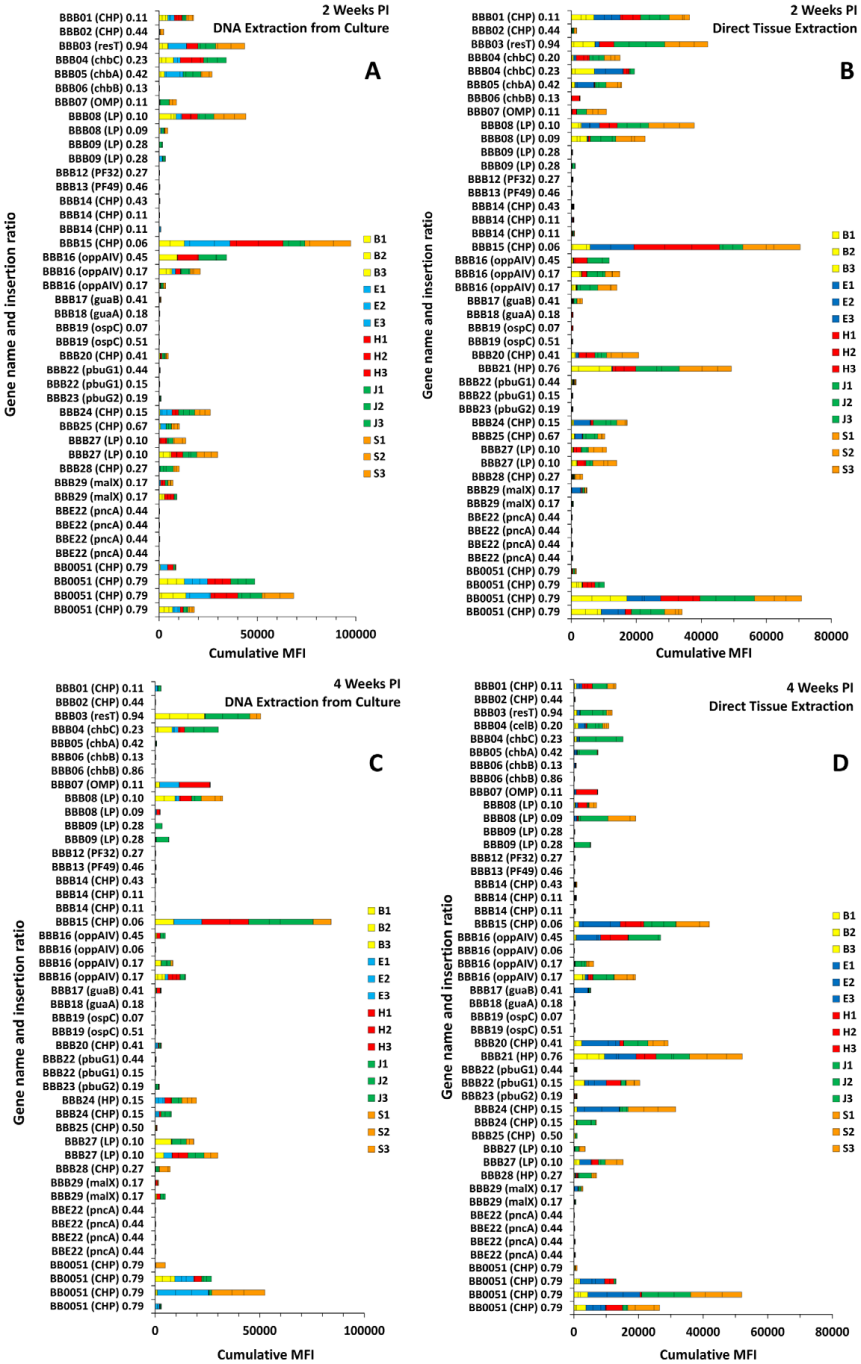
Luminex STM infectivity analysis of *B. burgdorferi* clones with transposon insertions in 26 of the 29 cp26 genes (*bbb01-bbb29*). Results are shown as cumulative MFI for bladder (B), ear (E), heart (H), tibiotarsal joint (J), and inoculation site (skin, S) samples from 3 mice per time point. A and B, 2 weeks PI, C and D, 4 weeks PI. A and C are results from cultured specimens, whereas B and D utilized DNA extracted directly from the tissue specimens. The Y axis indicates the gene number, name, and insertion ratio of each transposon insertion. *bbe22* and *bb0051* mutants are negative and positive controls, respectively, from the experiments in this analysis. Some mutants were analyzed by the direct DNA extraction method only (*e.g.* the *bbb21* mutant). Independent clones with different insertions in same gene (as indicated by different insertion ratios) were analyzed and some clones were analyzed multiple times in different STM sets to evaluate the reproducibility of results. HP, hypothetical protein; CHP, conserved hypothetical protein; LP, lipoprotein; OMP, outer membrane protein; OSP, outer surface protein.

In this article, we report the STM analysis results from 434 STM transposon mutants, representing 28,371 individual MFI measurements (Spreadsheet S2). To provide an example of the results obtained, the evaluation of mutants in the plasmid cp26 is provided in Fig. 4 and Table 3 in File S1. Negative and positive controls consisted of mutants in *pncA* and the chromosomal conserved hypothetical protein gene *bb0051*, respectively; the results obtained for these controls in three infectivity experiments are shown at the bottom of each panel. We found that the cumulative (or average) MFI value provided the most reliable indicator of the infectivity of a given mutant (see Materials and Methods). In general, there was good correlation between the cumulative MFI values obtained with the tissue DNA extraction and culture DNA extraction methods (Figs. 3A and 3C, and Figs. 3B and 3D) as well as between the 2 week and 4 week post inoculation results (compare Figs. 3A and 3B, and Figs. 3C and 3D). Additional information regarding the clones tested and tabular results using a positive/negative cutoff of 100 MFI for each tissue are provided in Table 3 in File S1.

**Figure 5:**
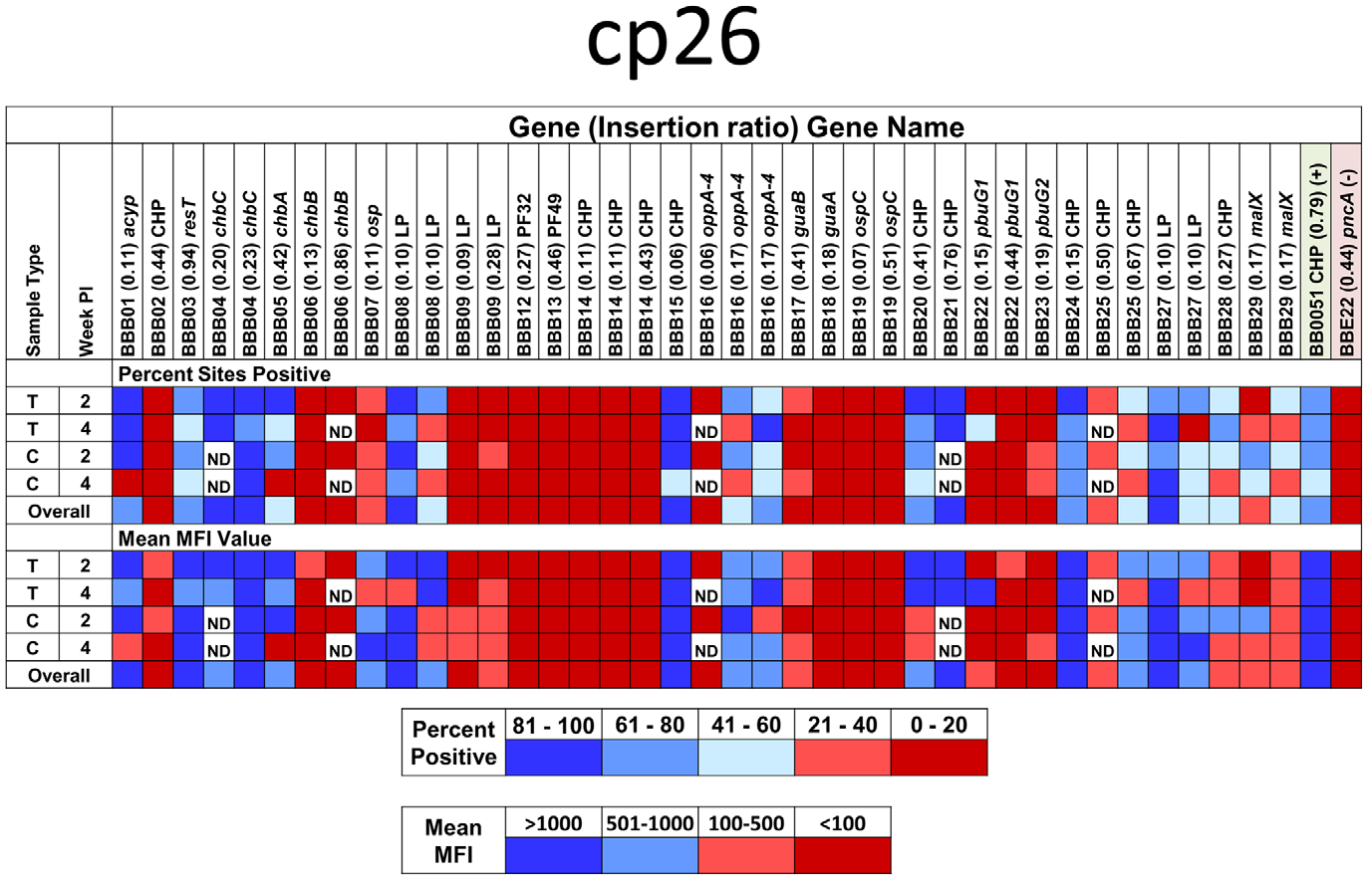
A simple heat map representation of the mouse infectivity of transposon mutants in cp26 genes. For each clone, results from Luminex STM analysis (see Fig. 4, Table 3 in File S1) were grouped by week post inoculation (2 weeks or 4 weeks) and by DNA preparation method (Culture  =  use of organisms cultured from tissue; Tissue  =  use of DNA extracted directly from tissue). The data in each group were scored according to the percentage of samples with MFI values above the negative threshold (100) or to the mean MFI value; each result was then color coded as indicated in the key. Each colored box in the figure corresponds to 12 to 15 data points (typically 5 tissues from 3 mice). Heat maps for other plasmids and gene function groups are provided in Figs. S7 and S8. The composite results obtained in all experiments for the positive (*bb0051*) and negative (*pncA*) control mutants are provided at the right side of the figure. Abbreviations are as described in the Figure 4 legend.

**Figure 6:**
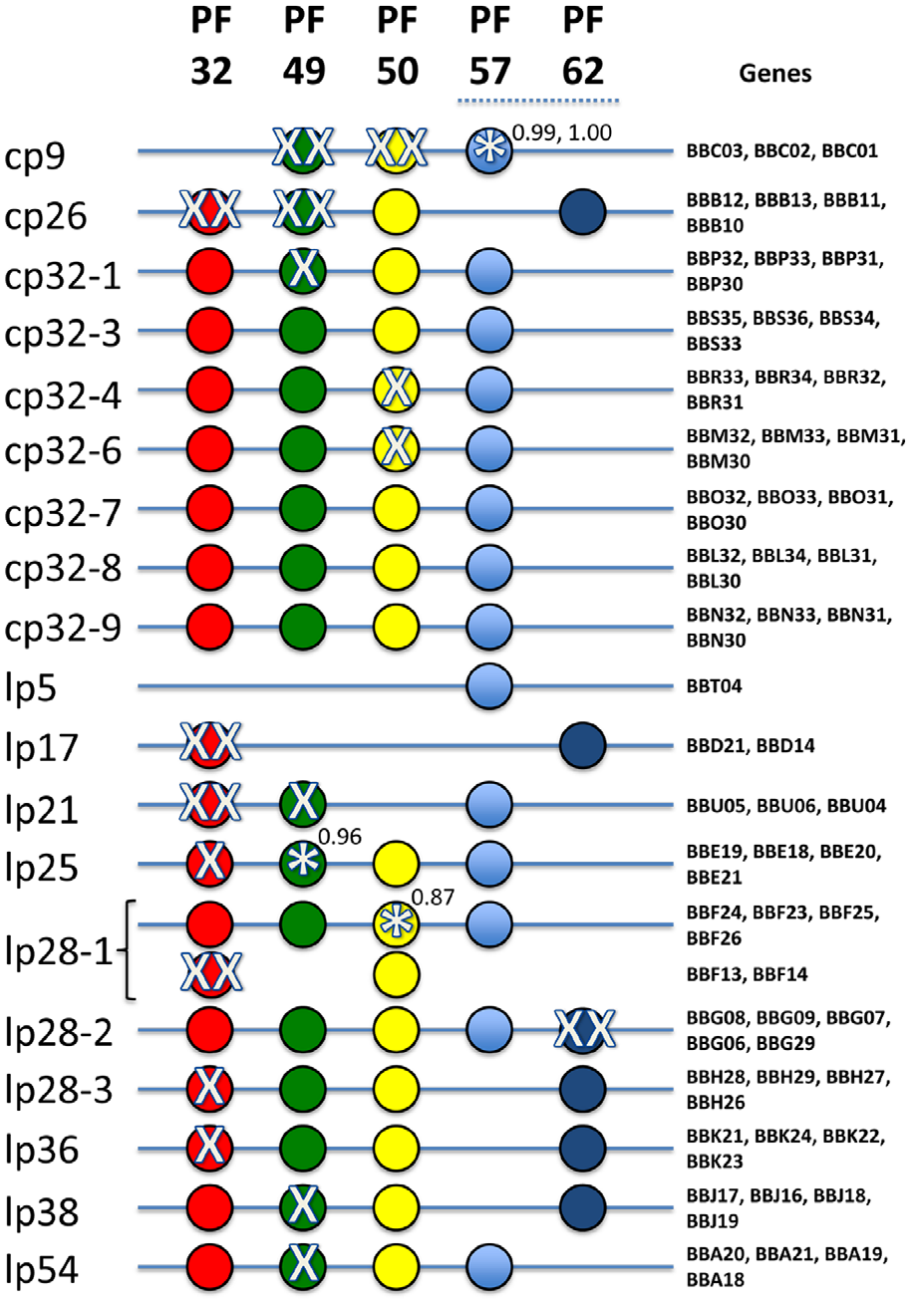
Transposon mutations in Protein Family (PF) genes postulated to be involved in plasmid maintenance. PF gene families are listed across the top. Presence of a full-length PF gene of each family is indicated by a colored circle. The plasmid and plasmid-associated PF genes are indicated on the left and right sides, respectively; the PF genes are listed in the same order (left to right) as in the figure. lp28-1 has two PF gene loci. Genes with multiple transposon insertions are marked as XX, whereas those with single mutations have a single X. Genes that have insertions only at the end of the gene are marked by an asterisk; the adjacent number indicates the insertion ratio.

Although infectivity patterns for individual clones could be discerned easily by examining the set of 60 data points, there was considerable variability among the individual MFI values obtained (Fig. 4). Even in clones with high infectivity phenotypes, 8% of individual samples processed by culturing the organisms from the tissue specimens had MFI values less than 100 (Fig. 4 in File S1); the proportion was higher (14% to 17%) in samples prepared by direct extraction of tissue, presumably due to the lack of ‘amplification’ resulting from multiplication of the organisms in culture. As expected, the percentage of MFI<100 samples increased in intermediate and low infectivity clones (Fig. 4 in File S1). To determine whether the Luminex procedure yielded reproducible values, we repeated the Luminex assay 6 times with the same inoculation site specimen from STM set 51. The results indicated that the MFI values were well clustered for each of the 11 clones in the set, and reinforced the occurrence of low, intermediate and high MFI values (Fig. 5 in File S1). It was possible that the variation observed was due to uneven distribution of organisms, in keeping with the paucibacillary nature of *B. burgdorferi* infection. Indeed, there was a low quantitative correlation between the culture- and direct DNA extraction-derived MFI values obtained from the same tissue, which utilized neighboring tissue samples. To examine this possibility, we subdivided STM set 51 inoculation site specimens from 6 mice (three each at 2 weeks and 4 weeks) into 9 neighboring samples. The DNA was extracted from each piece and then tested using the Luminex assay. The data in Fig. 6 in File S1 indicates that there is considerable variability in the MFI values for high infectivity clones in each of the 6 animals. The low MFI values obtained for the pncA mutant T01P01A01 were all within the range obtained with ‘no DNA’ controls. These results suggest that sampling error due to the uneven distribution of organisms accounts for much of the MFI value variation observed for individual clones.

A summary of the gene disruption and infectivity results obtained is provided in Table 3. The essentiality of certain gene function groups required for *in vitro* culture is based on the initial isolation of clones, as described above. In terms of mouse infectivity, a given clone was considered to be defective in infectivity if less than 20% of specimens yielded MFI values above 100. The requirement of several genes in a functional group provided strong evidence that the associated activities are essential for mouse infection under the conditions tested.

### Roles of Plasmid-encoded Genes in Mouse Infectivity

Plasmid-encoded genes play a vital role in the enzootic cycle of Lyme *Borrelia*. As shown in Fig. 4 and Table 3 in File S1, the results obtained with mutants in cp26 provide evidence that a number of genes encoded in this plasmid are needed for full infectivity in mice.

The STM analysis provides a large amount of data that is challenging to interpret *en masse*. To provide a clearer view of genes required for infectivity in mice, a simple heat map approach was developed that provides a visual overview of the infectivity of mutants in these gene groups. We analyzed groups of mutants based on the genes associated with certain plasmids or with different functional groups. An example of a heat map for genes associated with cp26 is shown in Fig. 5; additional maps for lp25, lp28-1, lp36, and lp54 are shown in Fig. 7 in File S1. In the upper portion of each panel, the coloration is based on the percentage of samples with an MFI greater than 100 (percent positive); very similar patterns were obtained using the mean MFI, i.e. the mean of the 12 to 15 MFI values for each time point and DNA preparation method (lower portion of each panel).

Overall, a surprisingly high number of the plasmid genes appear to be required for full mouse infectivity. Using the conservative cutoff of ≤20% of tissue sites positive and an overall mean MFI ≤100, transposon insertions in the following cp26 genes appeared to engender defective infectivity: *bbb02* (HP), *bbb06* (*chbB*), *bbb09* (HP), *bbb12* (PF32 plasmid maintenance protein), *bbb13* (PF49 plasmid maintenance protein), *bbb14* (CHP), *bbb18* (*guaA*, GMP synthase), *bbb19* (*ospC*), and *bbb22* (*pbuG1*, purine permease G1) (Figs. 3 and 4, Spreadsheet S2). In addition, mutations in other genes, including *bbb07* (a putative outer surface protein), *bbb17* (*guaB*, inosine-5-monophosphate dehydrogenase), and *bbb23* (*pbuG2*, purine permease G2) yielded reduced MFI values in most tissues. Thus 12 of the 26 cp26 genes examined (46%) are virulence determinant candidates based on this STM analysis; 5 of these genes had been identified as virulence determinants previously [15], [16]. Similarly, a high proportion of potential virulence determinants were also identified in the other plasmids examined to date (Fig. 7 in File S1, Spreadsheet S2).

### Functional Groups in Infectivity

Transposon mutations in genes associated with DNA recombination and repair, chemotaxis, motility, the PEP-PTS, other transport systems, plasmid maintenance, gene regulation, sRNA genes, carbohydrate, amino acid, and nucleic acid and other important metabolic pathways, predicted lipoproteins, conserved hypothetical proteins, hypothetical proteins (in the chromosome and some plasmids), protease, and complement regulator-acquiring surface proteins (CRASPs) of *B. burgdorferi* were selected for STM screening. A representative number of mutants in intergenic regions were also tested for infectivity to evaluate potential polar effects, small RNA genes, and regulatory regions. The results for some of these functional groups are depicted in the heat map format in Fig. 8 in File S1. Mutations in 10 of 14 chemotaxis genes resulted in an severe loss of infectivity (Fig. 8A in File S1). Mutants in *cheR-2* and *cheB-1* appeared to have reduced infectivity, whereas a mutant in *cheW-3* exhibited near wild-type infectivity. In *B. burgdorferi* B31, these genes have a total of 2, 2, and 3 paralogs, respectively. The results obtained for two methyl-accepting chemotaxis protein 5 (*mcp5*) mutants were quite different, so the findings for this gene were inconclusive.

Similarly, mutations in 4 of 7 genes associated with flagellar structure or assembly showed reduced infectivity, with only mutations in *fliD* and *flgB* yielding a high infectivity phenotype (Fig. 8B in File S1). Although most STM mutants did not have obvious *in vitro* growth defects or morphologic changes, mutations in 7 motility and chemotaxis genes (*fliH*, *fliI*, *flbA*, *flaA*, *cheA-2*, *cheB-*2, and cheR*-2*) exhibited slow growth and elongated, string-like or rod-shaped morphology. Additionally, 7 mutants (*flaA*, *flgI*, *fliG-1*, *fliW-1*, *cheA-2*, *cheR-2*, and *mcp-5*) had reduced motility, and string-like mutants (*flbA*, *fliH*, and *fliI*) were nearly non-motile, ‘trembling’ in a few sites of the cell. Overall, the results obtained were consistent with a requirement for chemotaxis and motility activities for mouse infection.

The results obtained with representative mutations in PEP-PTS genes are shown in Fig. 8C in File S1. *ptsG* encodes a glucose-specific IIBC component and appears to be required for mouse infection. In contrast, mutation of *ptsH-1* (*bb0448*, one of two genes encoding phosphocarrier protein homologs) had little apparent effect on infectivity. No mutants in *ptsH-2* (*bb0557*) or genes encoding other ‘core’ PEP-PTS components (*e.g. crr*, *ptsI,* and *ptsP*) were isolated. Mutations in the glucose and maltose-specific IIABC component homolog genes *malX-1* (*bb0116*) and *malX-2* (*bbb29*, located in cp26) yielded intermediate to low infectivity results. Mutants in the cp26-encoded chitobiose IIC and IIA components *chbC* and *chbA* were infectious, although the *chbA* mutant may have reduced infectivity. Two different mutants in the neighboring chitobiose IIB component *chbB* had low mouse infectivity in this system. Low infectivity was also obtained with a mutant in *fruA-1* (*bb0408*, fructose-specific IIABC component). Inconsistent results were obtained with two mutants in *fruA-2* (*bb0629*), precluding interpretation in the absence of additional information.

In terms of other transporter systems (Fig. 8D in File S1), mutation of the genes encoding lactose permease (*lctP*), Na+/H+ antiporter proteins (*nhaC-1* and *nhaC-2*), the ATP-binding protein of the methylgalactose transporter (*mglA*), and another ABC transporter ATP-binding protein (*bb0573*) appeared to result in loss of infectivity. Mutation at the very end of *proX* (*bb0144*, insertion ratio  = 0.99) resulted in a low infectivity phenotype, suggesting that the C-terminus is required for function; the downstream conserved hypothetical protein (CHP) gene *bb0143* is oriented in the opposite direction, ruling out polar effects. While *Borrelia* efflux system genes *besC* and *besA* appear to be required for full infectivity, mutation of the downstream gene *besB* had no apparent effect on infectivity. Intermediate infectivity results were generally obtained for mutations in the oligopeptide periplasmic binding protein genes *oppA-1* through *oppA-5* (Fig. 8D in File S1). No transposon insertions were observed in the 6 other genes for this ABC transport system; thus it is likely that the oligopeptide transport function is required for *in vitro* growth, but that the periplasmic binding proteins have the ability to partially complement one another, permitting *in vitro* culture of clones having a mutation in one *oppA* gene.

### Analysis of Genes Implicated in Plasmid Maintenance

Genes potentially required for plasmid replication or maintenance were investigated based on the mapping of transposon insertions in plasmid genes previously postulated to be involved in plasmid replication, partitioning, or retention [4], [5]. Genes representing five Paralogous Families (PFs) are present in both the linear and circular plasmids: PF32, PF49, PF50, PF57, and PF62 (Fig. 6). These PF genes are not consistently present in every plasmid, but all plasmids have either a PF57 gene or a PF62 gene. There are also many PF pseudogenes (gene fragments) in the plasmids; these are presumably nonfunctional and were not considered in this analysis. Transposon insertions were identified in 20 of the 72 intact PF genes, but in two cases (cp9 PF57 and lp25 PF49) the genes had insertions only within the last four percent of the reading frame and were likely to be functional (Fig. 6). An apparently functional copy of either PF57 or PF62 was retained in each plasmid for all transposon mutants recovered, consistent with the proposed requirement for one or the other of the corresponding protein products for the initiation of plasmid-specific replication [47]-[49]. Transposon insertions in PF32, PF49, and PF50 genes were obtained, but the pattern was not consistent in the different plasmids. The occurrence of multiple transposon insertions in the lp17 PF32 gene *bbd21* is in agreement with a prior study showing that *bbd21* is not required for lp17 replication and maintenance [47].

We were surprised to find that very low MFI signals were recovered from infected mice in nearly all of the mutants in these PF genes examined to date (Fig. 8E in File S1). The sole exception to this trend was in a mutant of the lp28-1 PF32 gene *bbf13*, which exhibited full infectivity. However, in this case, *bbf13* is a redundant copy of another intact lp28-1 encoded PF32 gene, *bbf24* (Fig. 6, Fig. 3 in File S1).

### Tick Inoculation Studies

To date, attempts to implement the STM procedure in tick transmission studies have not been successful due to insufficient recovery of organisms from ticks infected by capillary feeding with mixtures of *in vitro* cultured *B. burgdorferi*, as well as from mice that were exposed to these ticks. To determine whether results obtained by needle inoculation/STM analysis were comparable to those obtained with tick transmission, we inoculated *I. scapularis* nymphs with individual transposon mutant clones by capillary feeding and then fed the ticks on C3H/HeN mice, as previously described [50]. Results obtained with transposon mutants in genes involved in flagellar assembly and chemotaxis are shown in Table 4. For each of the clones tested, a high proportion of ticks contained viable *B. burgdorferi* after capillary feeding with suspensions of *in vitro* cultured organisms, as determined by culture of tick tissue and detection of spirochetes in tick smears by immunofluorescence (“unfed ticks”). With the exception of the methyl-accepting chemotaxis protein-5 (*mcp5*) mutant MG064, all of the mutants proliferated after the infected ticks were fed on naïve C3H/HeN mice. For MG064, none of 21 ticks were positive for *B. burgdorferi* by culture or immunofluorescence after the blood meal was taken. Cultures from multiple tissues (ear, heart, joint, bladder) and seroconversion for antibodies against the VlsE C6 peptide indicated that most of the motility and chemotaxis mutants had profound defects in infectivity, as compared to consistent culture and serologic positivity in the B31 5A18NP1 parental strain controls (Table 4). For comparison, the corresponding STM analysis results obtained by needle inoculation with these mutants (and one additional *mcp5* mutant) are provided on the right side of Table 4; the MG064 *mcp5* transposon mutant was not tested by STM analysis because it was created prior to the construction of the STM library [51] and therefore lacks a signature tag. In the STM studies, each of the motility and chemotaxis mutants in this group exhibited reduced infectivity (right side of Table 4); although the mutants in *flaA, cheR-2,* and *mcp5* genes had several sites with MFI values >100, the overall mean MFI values for these clones were low relative to the positive control (*bb0051* mutant T02P01A01, shown at the bottom of the table).

**Table 4:** Effects of transposon mutations in genes involved in flagellar assembly and chemotaxis on *I. scapularis* nymph transmission of *B. burgdorferi* clones to C3H/HeN mice.^a^

					Unfed Ticks		Fed Ticks		Mouse Infection by Tick Transmission		Mouse Infection by Needle Inoculation (STM Results)	
Gene Name	Gene No.	Clone	Insertion Ratio	Exp.(STMSet)	Positive Cultures	Mean No. of spirochetes per Field^c^ + SD	Positive Cultures	Mean No. of spirochetes per Field^c^ + SD	Positive Cultures/Total (Mice Positive/Total)	Mice with Positive Serology^d^/Total)	Positive Sites	Mean MFI Value
**Flagellar Assembly Genes**												
*fliH*	*bb0289*	T05TC243	0.29	4	10/10	0.10±0.06	15/22	1.72±4.31	0/12 (0/3)	0/3	10/60	72
*fliI*	*bb0288*	T10TC091	0.79	4	5/10	0.05±0.04	21/27	3.56±5.95	0/12 (0/3)	0/3	0/60	24
*flbA*	*bb0297*	T04TC041	0.65	5	9/10	0.13±0.20	12/14	1.09±1.50	0/12 (0/3)	0/3	1/60	30
*flaA*	*bb0668*	T06TC135	0.23	5	9/10	0.05±0.05	11/14	1.11±1.65	0/12 (0/3)	0/3	13/60	132
**Chemotaxis Genes**												
*cheA-1*	*bb0567*	T04TC006	0.46	6	10/10	0.08±0.06	27/27	2.97±4.80	0/12 (0/3)	0/3	0/60	25
*cheA-2*	*bb0669*	T10TC330	0.26	6	8/10	0.07±0.06	16/23	5.48±8.98	0/12 (0/3)	0/3	0/60	22
*cheR-2*	*bb0414*	T08TC168	0.37	6	7/10	0.07±0.05	23/23	7.78±8.60	0/12 (0/3)^f^	0/3	11/60	114
*mcp1*	*bb0578*	T09TC032	0.50	2	5/5	0.18±0.14	17/17	1.69±3.34	2/12 (2/3)	2/3	0/60	22
*mcp4*	*bb0680*	T07TC173	0.00	2	5/5	0.28±0.19	7/8	4.40±7.57	0/12 (0/3)	0/3	1/60	34
*mcp5*	*bb0681*	MG064^g^	0.79	1	5/10	0.40±0.28	0/21	0	0/12 (0/3)	0/3	ND^e^	ND
*mcp5*	*bb0681*	T08TC314	0.19	(42)	ND	ND	ND	ND	ND	ND	14/30	191
**Parental Strain Controls (Tick Studies Only)**												
–	–	B315A18NP1		1	8/10	1.06±0.33	26/26	24.43±24.12	12/12 (3/3)	3/3		
–	–	B315A18NP1		2	5/5	0.52±0.22	9/10	2.56±2.35	12/12 (3/3)	3/3		
–	–	B315A18NP1		3	10/10	0.75±0.61	25/25	9.86±6.59	12/12 (3/3)	3/3		
–	–	B315A18NP1		4	10/10	0.10±0.05	24/25	3.85±2.92	12/12 (3/3)	3/3		
–	–	B315A18NP1		5	6/10	0.02±0.03	11/12	2.69±2.13	12/12 (3/3)	3/3		
–	–	B315A18NP1		6	9/10	ND	16/16	ND	12/12 (3/3)	3/3		
**STM Positive Controls (STM Studies Only)**												
	*bb0051*	T02P01A01	0.79	(16)							55/60	2649
	*bb0051*	T02P01A01	0.79	(17)							53/60	2845
	*bb0051*	T02P01A01	0.79	(42)							26/30	1234

**Results obtained by needle inoculation and Luminex STM analysis are shown for comparison.^b^**

a Ticks were infected by capillary feeding with *in vitro* cultured *B. burgdorferi* and then fed on uninfected mice (see Materials and Methods). Separate groups of ticks were infected with each *B. burgdorferi* clone and used for tick inoculation of groups of 3 mice. The parental strain B315A18NP1 was used as a positive control in each of the 6 tick inoculation experiments shown.

b For comparison, needle inoculation STM results for the same clones are shown; these represent the cumulative results for all time points and tissues (see Spreadsheet S2). Results obtained for the STM positive control strain T02P01A01 in the corresponding STM experiments are provided at the bottom of the table.

c Number of spirochetes per field as detected by direct immunofluorescence with an anti-*B. burgdorferi* antiserum.

d Antibody to the VlsE C6 peptide.

e ND  =  Not Done.

f Culture positivity was due to the presence of a second clone with an unaltered *cheY-2* gene in the T05TC230 culture.

gMG064 is a transposon mutant from a non-STM library (D. J. Botkin and S. J. Norris, unpublished data). Chromosomal insertion site  = 721,311 bp.

## Discussion

### Library Characteristics and Essential Genes

In this study, we assembled a high density, sequence-defined signature-tagged transposon mutant library in an infectious *B. burgdorferi* strain. Sequence definition of the insertion points of 4,479 transposon mutants revealed that the insertions were distributed in all replicons of the *B. burgdorferi* 5A18NP1 genome, with an average of 2.68 unique insertion sites per kb DNA. The coverage of insertions in predicted genes was 34% in the linear chromosome, and ranged from 29% to 79% in the linear plasmids and 29% to 91% in circular plasmids (Table 2). The relatively low gene coverage in the chromosome was expected because of the high concentration of putative essential genes required for *in vitro* growth under standard conditions (in BSK II medium at 34 °C with 3% CO_2_). Clones with insertions that yield nonfunctional essential genes would not replicate during post-transformation selection, and thus would not be recovered. Similarly, disabling insertions in plasmid genes required for plasmid replication (and thus retention of the transposon-borne gentamicin resistance used in selection) would not be represented in the library. In genes with single insertions, over a third of the chromosomal insertion sites mapped to the final 10% of the gene (Fig. 2C in File S1), indicating that a high proportion of these late insertions were in essential genes but yielded functional products. The lower proportion of the insertions in plasmids were in the final 10% of genes, consistent with fewer plasmid genes being essential. Some plasmids had a higher number of insertions per kb of DNA and higher proportions of inactivated genes, most notably cp9, cp26, cp32-8, lp17, and lp36; others (*e.g.* cp32-1 and lp5) had lower insertion rates. The high A+T content, and hence the high density of TA *Himar1* insertion sites, is consistent throughout the genome. An exception is the high G+C content *vlsE* gene and *vls* silent cassettes; only a single insertion was observed in this region of lp28-1, and it is expected that additional properties of the DNA in this region may inhibit transposon insertion. lp5 likely has a low insertion rate because the high proportion of genes required for plasmid replication. Overall, the reason for varied insertion density in the plasmids is not well understood.

The ordered library is not saturating, but examination of genes as functional groups (Tables 3, 3 in File S1) provides insight into those functions that are essential for *in vitro* growth. As expected, DNA replication, transcription, and translation genes, as well those involved in energy generation and basic intermediary metabolism, were not disrupted. In addition, genes involved in peptidoglycan synthesis were unaffected, except for two predicted serine D-ala D-ala carboxypeptidase genes (*bb0582* and *bb0682*) and two genes with late insertions (*murD* and *murG*). In contrast, most genes encoding lipoproteins or other purported antigenic proteins (*e.g.* P35 and P37 paralogs), as well as those encoding chemotaxis, DNA recombination and repair, degradative enzymes, ‘transposon-like proteins’, annotated proteins of unknown function (which are essentially hypothetical proteins), and several transporter proteins, have disruptions (Table 2 in File S1). Thus the overall pattern of gene disruptions is consistent with expectations regarding the preservation of essential cellular functions. This analysis is of course restricted to gene products with predicted functions, which is further limited by the incomplete nature of the annotation.

For practical reasons, transposon mutant colonies were selected, cultured, and frozen without subjecting the cultures to a second subcloning. Some STM mutant isolates yielded unexpected infectivity results in the tick inoculation studies and were therefore assessed for possible cross-contamination with other mutants by PCR amplification using primers flanking the transposon insertion site followed by agarose gel electrophoresis. A few of these clones yielded two bands, consistent with a mixture of two *B. burgdorferi* clones with a wild type and transposon mutated regions. The results therefore had to be discarded and the mutants subjected to a second round of cloning. We thus recommend subcloning and insertion site PCR of transposon mutants (or other verification methods) prior to detailed analysis to avoid the possibility that the preparation contains more than one clone.

### Infectivity Assessment

We developed a novel Luminex-based approach for STM analysis of the mouse infectivity of the *B. burgdorferi* transposon mutants. Prior STM studies had typically utilized either hybridization to DNA arrays or individual PCR reactions to assess the presence of signature tags (and hence the corresponding STM clones) in a culture or tissue sample. The use of Luminex technology (Fig. 2) allows the assessment of each culture or tissue specimen in a single well of a 96-well plate, thus providing a high-throughput method. In most experiments in this study, we performed parallel analyses of DNA from cultures and DNA extracted directly from each tissue specimen, and found the results from these two methods to be comparable. Therefore, in later experiments only DNA directly prepared from tissue was used. Five tissues per mouse were examined in most STM sets (Fig. 3), representing those sites with pathologic changes in humans and mouse models (joint and heart) as well as other sites commonly used for culture assessment of mouse infection (ear and bladder). Skin from the inoculation site was included in later experiments to evaluate whether some clones may colonize locally but be defective in dissemination. Also, two time points (2 and 4 weeks) with three mice each were used to potentially identify mutants that are able to establish infection but are later eliminated by the immune system (as occurs for *vlsE*-deficient mutants). This protocol resulted in 60 data points per clone in each STM experiment. We chose to limit the number of different sequence tag libraries to 11 because of the challenges of generating *B. burgdorferi* mutants and the paucibacillary nature of infection, which may potentially result in difficulty of detection if larger clone sets are utilized.

High, intermediate and low infectivity phenotypes were observed (Fig. 4 in File S1). A mutant in *bbe22* on lp25, encoding the nicotinamidase PncA, was selected as a negative control, because this gene was shown previously to be required for mouse infection [9], [52]. MFI values for this clone were typically less than 100 (mean  = 30), similar to controls in which template DNA was not included in the initial PCR reaction. High infectivity clones had specimens with MFI values >1000, but a surprisingly high proportion of samples (8 to 17 percent) had low MFI values. For intermediate infectivity clones, such as T02P01A01 (a mutant in the chromosomal conserved hypothetical protein gene BB0051), many samples yielded MFI values <100, but the remainder had a broad range of values ranging up to 6000. These results indicate that both high and intermediate infectivity clones yield ‘negative’ samples, but the proportion is higher in intermediate infectivity clones. This finding in turn suggests that clones are not evenly distributed in tissues, but instead may form loosely arranged ‘colonies’ of progeny within tissues following dissemination.

This interpretation is further reinforced by studies in which single skin inoculation site specimens from each of 6 mice were subdivided into 9 neighboring pieces and then examined for the presence of infecting clones by DNA extraction and Luminex analysis (Fig. 6 in File S1). In this experiment, only T02P01A01 (*bb0051* mutant) and T07TC483 (*bb0545* inositol monophosphatase mutant) clones were infectious. For the tag 7 clone T07TC483, the 9 neighboring tissue specimens exhibited a broad range of MFI values in each mouse; in the three mice examined two weeks post inoculation, these values ranged from 22 to 780, 204 to 1357, and 255 to 1321, respectively. Similar results were obtained using data from the Tag 2 clone in this experiment, as well as a universal primer that produces fluorescent product for all clones in the population. Such sampling variability is not unexpected, in that culture positivity (which yields only a +/− result) is often also variable in mouse infectivity studies. In contrast, low infectivity clones consistently yielded low MFI values; for example, the *pncA* mutant T01P01A011 yielded a range of MFI values of 4 to 57 (mean ± SD of 34±17) in the three mice examined at two weeks post inoculation.

### Plasmid Genes and Infectivity

Prior studies had shown that, in *B. burgdorferi* B31, genes present in the linear plasmids lp25, lp28-1, lp36, lp54, and cp26 are required for full infectivity in mice [8]–[14]. Plasmid lp25 carries *pncA*, *bptA*, and *bbe31*; these genes encode the nicotinamidase PncA required for spirochete survival in mice [9], BptA, a protein essential for tick colonization and also implicated in mouse infection [13], [53], and BBE31, a protein that promotes migration of *B. burgdorferi* from the tick midgut to the hemolymph and salivary glands during feeding [54]. The *pncA* gene was disrupted in five independent mutants in the STM tranposon mutant library, and one of the *pncA* mutants (T01P01A11) was selected as a negative control in our STM screening system. The plasmid lp28-1 contains the *vls* antigenic variation system, which is required for immune evasion and long-term infection in mammalian hosts [22], [23]. Loss of plasmid lp36 is also associated with low infectivity in mammals, and Jewett et al. [14] established that infectivity could be largely restored by complementation with *adeC* (BBK17), encoding an adenine deaminase involved in adenine-hypoxanthine interconversion. The circular plasmid cp26 carries several essential genes required for *in vitro* growth, including the telomere resolvase *resT*, BBB29, BBB26, and BBB27 [15], [16]. The cp26-encoded surface lipoprotein OspC is also required for early colonization in the mammalian host [17]–[21]. Outer surface protein A (OspA) and OspB, encoded by lp54, bind to a tick receptor protein, TROSPA, and play an important role in spirochete colonization in the tick vector [24]–[28].

In the present study, we systematically examined mutants in the plasmids cp26, lp25, lp28-1, lp36, and lp54. Representative clones were selected for each gene disrupted, and plasmid content was evaluated to exclude mutants lacking plasmids known to be important in infectivity. Overall, our results reinforce prior findings and greatly extend the number of genes in these plasmids in which infectivity has been investigated. As emphasized previously, the findings we report here should be considered preliminary, and must be confirmed through studies involving individual mutants and complemented clones. Mutations in putative plasmid maintenance genes will be discussed in a separate section.

In cp26, mutants in genes encoding OspC, GuaA, GuaB, PbuG1, and PbuG2 all exhibited reduced infectivity, as reported previously [15]–[21]. Additionally, two mutants in *chbB*, encoding a PEP-PTS system IIB chitobiose transporter protein, exhibited low infectivity. Surprisingly, mutations in the other two PEP-PTS transport genes in this gene cluster, *chbC* and *chbA*, appeared to retain mouse infectivity (albeit to a reduced extent in the *chbA* mutant). Chitobiose is a dimer of N-acetyl galactosamine, and is a subunit of chitin, the major constituent of the arthropod exoskeleton. Therefore it is believed that the *chb* transport system is important in acquisition of N-acetyl galactosamine by *Borrelia* species in the tick host [55], [56]. Mutations in *bbb02* and *bbb14*, genes of undefined function that are well conserved in all *Borrelia* species, also engendered a reduced infectivity phenotype. Interestingly, a mutant in the essential telomere resolvase gene *resT* (clone T08TC298, insertion ratio 0.94) was infectious and had no obvious growth defect *in vitro*. These results indicate that this mutant may express a truncated yet functional ResT protein, and that this late insertion of transposon in *resT* gene did not greatly affect ResT function.

As expected, mutations in the lp25 genes *bptA* and *pncA* resulted in reduced infectivity (Fig. 7B in File S1); no transposon insertion in *bbe31* was obtained. Additional mutations in lp25 genes that substantially reduced infectivity included those in *bbe04.1*, *bbe09*, *bbe24*, and *bbe29.1*. *bbe09* encodes a predicted lipoprotein of unknown function of the P23 family with orthologs in other *Borrelia*. *bbe04.1* was identified initially as a P23 pseudogene, and is no longer annotated. *bbe24* represents a putative pseudogene (no longer annotated) that encodes a fragment of a PF49 putative plasmid partition protein; this open reading frame is intact and conserved in other *Borrelia* strains. *bbe29.1* is a another pseudogene in the *B. burgdorferi* B31 genome, and appears to encode a fragment of an adenine specific DNA methyltransferase. Because the B31 genome was one of the earliest microbial genomes completed [4], [5], it would be of interest to identify the ORFs present in lp25 and other plasmids after resequencing and reevaluate the genes affected by these transposon insertions.

In lp28-1, the only genetic locus other than the *vls* antigenic variation system that had been implicated in Lyme borreliosis pathogenesis is *bbf01*, encoding the 37 kDa arthritis-related protein Arp. In previous studies, antisera against Arp reduced arthritis and carditis histopathology in the mouse model, but did not affect the numbers of spirochetes present in mouse tissues [57]–[59]. In our analysis, mutation of *bbf01* did not greatly influence infection as measured by the Luminex PCR detection method. Candidates for other lp28-1 genes involved in infectivity include *bbf03*, *bbf05*, *bbf10*, and *bbf18* (Fig. 7C in File S1). *bbf03* encodes RepU, a member of protein family HMM PF02524. This family contains the KID amino acid repeat found in *Borrelia* spirochete RepA/Rep+ proteins. The function of these proteins is unknown, but RepA and related *Borrelia* proteins have been suggested to play an important genus-wide role in the biology of the *Borrelia*. *bbf05* is a truncated member of the *Borrelia* PF57 family, also known as protein family 02414. The protein encoded by *bbf10* is identical in amino acid sequence to a portion of the *B. burgdorferi* B31 lp36-encoded protein BBK41, which has been identified in the several other *B. burgdorferi* strains. *bbf25* encodes a fragment of a transposase-like protein.

lp36 has a total of 60 annotated genes (including one small RNA), and in this study we examined mutants in 40 of these predicted genes (Fig. 7D in File S1). In concordance with prior results by Jewett et al. [14], we found that mutation of the adenine deaminase gene *adeC* resulted in reduced mouse infection. In contrast, mutation of *bbk32*, encoding a well-characterized fibronectin-binding protein, had little apparent effect on infectivity; prior studies had yielded contradictory information regarding the requirement of BBK32 for infectivity [60]–[62]. STM results further indicated decreased infectivity for mutants in a region encompassing *bbk02.1* to *bbk04*. These predicted ORFs are overlapping, and in other Lyme *Borrelia* strains this region contains a larger gene encoding a predicted restriction modification protein. Mutations in other conserved hypothetical protein genes that appear to have reduced infectivity include those in *bbk05*, putative lipoprotein gene *bbk07*, *bbk13*, *bbk45*, and *bbk46* through *bbk50*. *bbk13* encodes a member of the SIMPL protein family, which is conserved in organisms ranging from bacteria to mammals and may play a regulatory role. Homologs of *bbk46* through *bbk50* are found in other Lyme borreliae, but not in relapsing fever organisms or other bacteria.

lp54 contains a high proportion of genes in which transposon disruption results in decreased infectivity (Fig. 7E in File S1). Most of these encode proteins without a predicted function that are found only in *Borrelia* species; several are evident only in Lyme disease *Borrelia*. In prior studies, mutation of *dbpA* reduced mouse infectivity by needle inoculation but not by tick-mediated infection [63]. In our analysis, *dbpA* mutants appeared to have decreased infectivity by needle inoculation, although the degree of attenuation was different between the two mutants tested. As expected, mutation of *ospB*, encoding a surface lipoprotein expressed predominantly in the tick environment, had little apparent effect on mouse infection. *bba03* encodes a predicted outer membrane lipoprotein, and its mutation has a marked effect on mouse infectivity. The gene encoding outer membrane protein Oms28 (*bba74*) [64]–[66] also appears to be required for full infectivity.

### Analysis of Functional Groups

We decided to systematically examine groups of mutants in important functional categories to provide insight into the importance of those gene groups in mammalian infection. The groups analyzed included those involved in DNA recombination and repair, chemotaxis, motility, the phosphotransferase system, other transport systems, plasmid maintenance, genes with potential roles in gene regulation, sRNA genes, and genes involved in carbohydrate, amino acid, and nucleic acid metabolic pathways. In addition, mutants in the genes of predicted lipoproteins, conserved hypothetical proteins, hypothetical proteins (in the chromosome and some plasmids), protease genes, and complement regulator-acquiring surface proteins (CRASPs) of *B. burgdorferi* were selected for STM screening. A representative number of mutants in intergenic regions were selected and examined to test for potential polar effects, small RNA genes, and regulatory regions.

Chemotaxis is an important virulence determinant in most pathogenic bacteria. In a recent study, Sze et al. [67] deleted *cheA-2*, encoding one of two chemotaxis histidine kinases, in an otherwise infectious clone of *B. burgdorferi* B31. *In vitro*, this mutation results in constant running motility, with no reversals or flexing. Sze et al. found that the *cheA-2* mutant was noninfectious by either needle inoculation or tick transmission, although the organism was able to survive within ticks. In our analysis, nearly all of the 21 mutants in 14 chemotaxis genes had severely reduced mouse infectivity (Fig. 8A in File S1), indicating that the chemotaxis pathway as a whole is critical to infectivity. *B. burgdorferi* contains multiple paralogs of some of the chemotaxis protein genes (2 *cheA*, 2 *cheB,* 2 *cheR*, 3 *cheW*, and 2 *cheY*), and the encoded proteins may provide roles specific to certain stages of the enzootic cycle. An example is provided by the *cheW* genes, which encode proteins involved in coupling CheA to the chemoreceptor array [68], [69]. In the STM analysis, *cheW-3* is apparently not required for mouse infection by needle inoculation, whereas mutation of *cheW-2* results in greatly reduced mouse infection (Fig. 8A in File S1). Interestingly, the *cheY-2* mutant we tested appears to be non-infectious, whereas Motaleb et al. [70] found that only mutants of *cheY-3* (and not of *cheY-1* or *cheY-2*) had a defect in reversal and flexing. Overall, our results indicate that nearly all of the *B. burgdorferi* chemotaxis proteins are required for mouse infectivity.

Not surprisingly, the genes involved in flagellar structure and assembly that we examined also appear to be required for mouse infection (Fig. 8B in File S1). Many of these mutants also had reduced motility, division defects (resulting in elongated organisms), and structural changes in the flagellar motor (unpublished data). The growth defect of each mutant may be related to the *in vivo* infectivity phenotype. These findings will be the subject of a separate article.

In other bacteria, the PEP-PTS is involved in carbohydrate transport and phosphorylation, and also plays an important regulatory role in terms of carbon catabolite repression through adenylate cyclase and cAMP levels [71]. The *B. burgdorferi* PEP-PTS system is unusual, in that maltose- and chitobiose-specific PTS components are encoded on a plasmid, cp26. We obtained low infectivity results with mutants in the genes encoding the glucose-glucoside specific IIBC component PtsG, the chitobiose-specific IIBC component ChbB, and the fructose-mannitol specific IIB component FruA-1 (Fig. 8C in File S1). Intermediate loss of infectivity was indicated for mutants in the maltose-specific IIBC components MalX-1 and MalX-2, encoded on the chromosome and cp26, respectively. The other cp26-encoded chitobiose-specific components, ChbC (permease) and ChbA (phosphotransferase protein), do not appear to be required for mouse infection by needle inoculation; indeed, the Chb system overall is thought to play important roles in the utilization of chitobiose in the tick environment [55], [56]. *B. burgdorferi* encodes an adenylate cyclase homolog CyaB (BB0723), but no catabolite repression protein (Crp) gene has been identified. Mutation of *cyaB* results in an intermediate infectivity phenotype by STM analysis (Spreadsheet S2). Additional studies are needed to gain a better understanding of the role of the PEP-PTS in *B. burgdorferi* pathogenesis.

With regard to other transport systems (Fig. 8D in File S1), consistently negative infectivity results were obtained for mutants in genes encoding the lactose permease LctP, Na^+^/H^+^ antiporters NhaC-1 and NhaC-2, the glycine/betaine/L-proline ABC transporter periplasmic binding protein ProX, and the methylgalactoside ABC transporter ATP-binding protein MglA. Intermediate results were obtained with many other transport related genes, including those encoding the oligopeptide ABC transporter periplasmic binding proteins OppA-1 through OppA-5. Thus it is likely that many transport systems are required for mammalian infection.

### Analysis of Genes Potentially Involved in Plasmid Replication and Maintenance

Genes involved in plasmid replication and maintenance were investigated based on the mapping of transposon insertions in plasmid genes previously predicted [5] and experimentally investigated [47]–[49], [72], [73] to have this function. Genes representing five paralogous families are present in both the linear and circular plasmids: PF32, PF49, PF50, PF57, and PF62 (Fig. 6). PF57 and PF62 show limited homology and every plasmid has one of the two [5]. An apparently functional copy of either PF57 or PF62 was retained in each plasmid for all transposon mutants recovered (Fig. 6). Our inability to recover transposon insertions in either PF57 or PF62 (unless both were present) is consistent with the proposed requirement for one or the other of the corresponding protein products for the initiation of plasmid-specific replication [47], [49].

A complete set of replication/maintenance proteins is believed to comprise either PF57 or 62 along with the triad of PF32, 49 and 50. Of the 19 plasmids present in the B31 5A18NP1 clone, 15 carried a complete set of four replication/maintenance proteins. The four plasmids that did not (cp9, lp5, lp17 and lp21) were the four smallest plasmids that probably lost PF members during the deletion process that generated them. It is possible that cross-complementation by PF proteins encoded by related plasmids occurs. There are also many PF pseudogenes (gene fragments) in the plasmids; these were presumably generated during the frequent plasmid rearrangements observed in *B. burgdorferi* [74]; these fragments are believed to be nonfunctional and were not considered in this analysis.

Several additional points of interest from the data set are worthy of mention. The first is that the family of intact cp32 plasmids is exceedingly intolerant of insertions in the replication/maintenance proteins. Only three single insertion mutants were recovered (one in PF49 on cp32-1 and one each in PF50 in cp32-4 and cp32-6) out of more than 560 transposon insertion mutants on these plasmids. Four of the intact cp32 plasmids did not have any insertions in their replication/maintenance regions. In contrast, all the linear plasmids (with the exception of lp28-2) had at least one transposon insertion recovered in their replication/maintenance genes. A dramatic contrast between the intact cp32 family and the linear plasmids was the complete absence of any insertions into PF32 on the circular replicons, while multiple insertions were observed in this gene on the linear plasmids. The role of PF32 members currently remains unknown. They encode a Walker box ATPase [48] that physically interacts with the replication initiator protein, at least on lp17, and they appear to be expendable on many of the linear plasmids, but not on intact cp32 members. This stark contrast may be due to a varying requirement based upon the topological state of the DNA (supercoiled versus linear). Alternatively, because of the ongoing sequence exchange on the linear plasmids [5], [74], [75], the linear replicons may have evolved the ability for interplasmidic complementation of the PF32 function.

Tilly et al. [42] recently demonstrated that the cp26 genes *bbb10, bbb11*, and *bbb13* (encoding PF62, PF50, and PF49 paralogs, respectively) together are sufficient for maintenance of a partition-defective MiniF plasmid in a heterologous *E. coli* plasmid system; however, the PF32 gene *bbb12* was dispensable both in this system and for cp26 maintenance in *B. burgdorferi*. They were unable to isolate *B. burgdorferi bbb10* mutants, a result consistent with the requirement of this gene for maintenance of the essential plasmid cp26. In our study, we identified 9 independent transposon mutants in *bbb12* and 7 mutants in *bbb13*, but no mutants in *bbb10* or *bbb11* (Fig. 6; Spreadsheet S1). Thus the results for *bbb10*, *bbb11*, and *bbb12* are consistent with the Tilly et al. study [42]. The requirement for *bbb13* in the MiniF analysis but not in *B. burgdorferi* itself may be explained by interplasmidic complementation of PF49 in *B. burgdorferi* but not in the heterologous *E. coli* maintenance system. Further studies would be needed to prove or negate this hypothesis.

The multiple insertions in both PF32 and PF49 on cp26 contrast with the lack of disruptions in the corresponding genes in the cp32 plasmids. Although circular, cp26 is not a member of the cp32 family and has been proposed to have been generated by circularization of a linear plasmid [74], [76]. Its transposon insertion properties are, therefore, more consistent with its putative origin than with its current topological state. Further exploration of this conundrum may help explain the different results observed between the circular and linear plasmids in this study. Finally, the pattern of intolerance to transposon mutagenesis in the cp32 family versus the linear plasmids is consistent with the proposal that the linear plasmids evolved by linearization of the cp32 plasmids followed by evolutionary drift [74], and perhaps this process resulted in the observed differences in the transposon insertion tolerance in putative plasmid replication and maintenance genes.

We were surprised to find that nearly all of the putative plasmid replication/maintenance genes tested to date appear to be required for infectivity (Fig. 8E in File S1). Background MFI levels were obtained for mutants with transposon insertions in the PF32 members for plasmids cp26, lp21, and lp25; clones with transposon insertions in the PF49 genes of cp26, lp25, and lp54 also yielded low MFI values and were apparently noninfectious. A mutant in the lp17 PF32 gene *bbd21* had a reduced number of mouse tissues that had MFI values over 100 (Fig. 8E in File S1). As indicated previously, lp28-1 has a second locus of putative plasmid replication/maintenance genes that has PF32 and PF50 paralogs *bbf13* and *bbf14* (Fig. 3 in File S1). *bbf13* had multiple transposon insertions, and one of these clones (T08P01G01) was tested for infectivity. This clone yielded high MFI values in all tissue specimens tested (Fig. 8E in File S1), indicating that this apparently redundant copy of a PF32 gene is not required for infectivity. Of the PF50, PF57, and PF62 gene mutants isolated (Fig. 6), only the clone with a mutation in the lp28-1 PF50 gene *bbf25* (T04TC094) has been examined thus far. This clone was apparently noninfectious, indicating that the ‘redundant’ PF50 gene *bbf14* was unable to compensate for the loss of BBF25 function. Clones with intergenic mutations between *bbf12* and *bbf13* (T09TC271) and *bbf14* and *bbf14.1* (T06TC036) were infectious.

Without further information, these results must be interpreted cautiously. The pattern of transposon insertions in these putative plasmid replication/maintenance genes and corresponding infectivity results may indicate that this set of gene products fulfill important infectivity-related functions beyond plasmid maintenance. However, it is also possible that the insertions in these genes, although permissive of replication of the corresponding plasmid *in vitro*, may sufficiently impair plasmid maintenance during mouse infection to result in plasmid loss. The loss of the plasmid would thus result in the absence of MFI signal, which in our Luminex assay is dependent upon the presence of the transposon and its corresponding STM tag. Possible explanations for this loss of MFI signal in mice infected with PF mutants are currently under investigation.

### Comparison to Tick Inoculation

To assess whether the results obtained by needle inoculation with STM analysis were comparable to those obtained by the natural mode of transmission, we assessed 4 motility and 7 chemotaxis mutants for infectivity in ticks, and for their effective transmission via ticks to mice (Table 4). *I. scapularis* nymphs infected by capillary feeding of *B. burgdorferi* cultures were utilized, and provide an effective model of the natural infection cycle [50], [77]. Survival of mutant spirochetes in ticks prior to mouse feeding was comparable to that of the parental *B. burgdorferi* clone (B31 5A18 NP1), as assessed by numbers of ticks that yielded positive spirochete cultures *in vitro* and immunofluorescent detection of spirochetes in tick smears. All of the mutant clones (except the *mcp5* mutant MG064) also proliferated normally within the tick in response to feeding on mice. Therefore, spirochetal survival in flat and blood-fed ticks does not appear to be affected by the mutations, with the exception of mutation in one of three *mcp* genes tested; it is possible that Mcp5 plays an important role in signaling and *B. burgdorferi* survival in the tick environment. Infectivity in mice via ticks was evaluated both directly by *in vitro* culture of organs from mice (n  = 3 per mutant) that had been exposed to infected ticks, and indirectly by assessing the anti-VlsE-C6 antibody levels prior to tick feeding and at the time of necropsy. All four of the mutants in flagellar assembly genes and most of the chemotaxis mutants exhibited a lack of mouse infection, consistent with the low infectivity results obtained by needle inoculation and STM analysis. In contrast, mice exposed to ticks infected with wild-type spirochetes had anti-C6 antibody levels that were at least 10-fold higher than baseline values. Thus, both the STM analysis and tick inoculation studies indicate that motility is necessary for mammalian infection, consistent with other recent studies [39], [78], [79]. The STM needle inoculation results and the tick inoculation data were less concordant for the chemotaxis mutants (Table 4). The *cheR-2* mutant was noninfectious in the tick inoculation study, but exceeded the MFI value of 100 used for the negative cutoff in 11 of 60 tissue specimens in the STM study. However, only the culture method of STM analysis yielded MFI values over 100; it is possible that only a few organisms survived in the tissues but were detectable due to proliferation in culture. Two *mcp5* mutants with different insertion sites were tested separately in the tick inoculation and STM studies, so direct comparison may not be valid. In this case, the *mcp5* mutant tested by tick inoculation (MG064) was consistently noninfectious in mice, whereas the STM results with *mcp5* mutant T08TC314 had 14 of 30 tissue samples with MFI values exceeding 100. Despite these inconsistencies, it is clear that a fully functional chemotaxis system is required for mammalian infection. We plan to further examine the roles of motility and chemotaxis in the infectious in future studies.

### Conclusion

Based on the findings to date, the availability of an ordered, signature-tagged transposon mutant library in an infectious *B. burgdorferi* background will be of value in assessing the genes required for mammalian infection. The results indicate that a high proportion of genes are needed for mouse infection, including many that have no predicted functions. Thus it is likely that the continued analysis of the STM library will reveal novel pathogenic mechanisms, in keeping with the limited understanding of how invasive, non-toxigenic, persistent pathogens cause disease. The evaluation of multiple tissue sites at two different time points using Luminex technology provides a particularly robust data set for evaluation. We are also evaluating approaches that will enable STM analysis of *B. burgdorferi* throughout the enzootic cycle. However, as in any STM analysis, there are several reasons that the results must be considered preliminary indications of the roles of the virulence determinant candidates identified. First, infection with individual clones and complementation of mutants will be necessary to verify the importance of each gene in infection. Second, it is possible that polar effects on downstream genes are responsible for observed decreases in infectivity, although we have seen examples where transposon disruption of upstream genes had no apparent effect on downstream gene expression [50]. Third, the potential occurrence of cross-contamination within even well-isolated colonies may result in false positive results; thus, all STM mutants should be recloned prior to more thorough studies. Finally, it is possible that cross-complementation occurs between clones in an STM set, permitting infection by clones that are otherwise defective in activities required for tissue colonization and growth. However, Lyme disease *Borrelia* do not appear to secrete proteins or other macromolecules, and the possible involvement of quorum sensing molecules [80]–[86] or other small molecular weight compounds in infection is uncertain. Despite these limitations, we believe that the expanded, unbiased evaluation of *B. burgdorferi* genes will lead to a better understanding of the pathogenesis of Lyme borreliosis.

## Materials and Methods

### Ethics Statement

All procedures involving mice conducted at the University of Texas Health Science Center at Houston and the Tulane National Primate Research Center were reviewed and approved by the institutions’ Animal Welfare Committee and the Animal Welfare Assurance Committee, respectively. This study was conducted in accordance with all applicable federal, state, and institutional guidelines regulating research with animals, including the PHS Policy on Humane Care and Use of Laboratory Animals, The Guide for the Care and Use of Laboratory Animals, and the US Government Principles for the Utilization and Care of Vertebrate Animals Used in Testing, Research, and Training.

### Bacterial Strains and Growth Media

The strains used in this study were *B. burgdorferi* B31 clone 5A18NP1and *E.coli* TOP10. The infectious, moderately transformable *B. burgdorferi* B31 clone 5A18NP1 was used for generation of all STM mutants. 5A18NP1 is a genetically engineered clone in which plasmids lp28-4 and lp56 are missing and *bbe02*, encoding a putative restriction-modification enzyme, has been disrupted [30]. All strains used in this study had undergone no more than two subcultures since clone isolation prior to infectivity studies. *B. burgdorferi* were grown at 34 °C in 3% CO_2_ in Barbour-Stoenner-Kelly II (BSK-II) medium supplemented with appropriate antibiotics as described previously [87]. *E.coli* TOP10, a DH5*α*-derived strain obtained from Invitrogen Corporation (Carlsbad, CA), was used for the preparation of plasmids for electroporation into *B. burgdorferi*.

### Construction of Signature-tagged Suicide *Himar1* Delivery Vectors for Transposon Mutagenesis of Infectious *Borrelia burgdorferi*

The *Himar1-*based transposon vector pGKT [40] was graciously provided by Dr. P. E. Stewart (Rocky Mountain Laboratories, National Institutes of Health, Hamilton, MN). pGKT is a modified version of pMarGent [38]. pGKT contains a gentamicin resistance cassette gene with the *B. burgdorferi flgB* promoter (*flgB_Pr_:aacC1*) and a *colE1* origin of replication within the transposable element, as well as the highly active *Himar1 C9* transposase [34] and a constitutively expressed kanamycin resistance cassette with a *B. burgdorferi* promoter (*flaB_Pr_::aph1*) in the ‘backbone’ of the vector outside the transposable element (Fig. 1 in File S1). Thus gentamicin resistance in *B. burgdorferi* is conferred by transposition of the transposable element, whereas kanamycin resistance and transposase activity are lost once transposition has occurred. Dual selection with kanamycin and gentamicin selects for intact pGKT plasmid, and was found to be important in generating STM-tagged constructs. The 11 STM tags used in this study were based on previously published STM tags [88]; each contains a unique 7 bp sequence tag (Table 4 in File S1). Signature-tagged versions of pGKT were constructed by inserting paired oligonucleotides containing STM tags into a 45 bp region between the ColE1 origin and inverted terminal repeat 2 (ITR2) in the transposable element. The Tag 1 oligonucleotides (Table 4 in File S1) were first ligated into pGKT that had been treated with *Bsg*I and *Bsu*36I, producing pGKT-STM1. This modification also introduced *Bam*HI and *Kpn*I sites that were cleaved to provide an insertion site for the Tag 2 oligonucleotide pairs. *Xho*I and *Sph*I sites were added to the pGKT-STM2 insert sequence to provide additional flexibility for future constructs. pGKT-STM2 was then used as the source of plasmid DNA for creation of pGKT-STM3 to pGKT-STM11 through insertion of the corresponding oligonucleotide pairs. The sequence of the entire pGKT plasmid was confirmed, and the modified regions containing the STM tags were amplified by PCR and sequenced for each pGKT-STM construct. This transposon vector set could easily be expanded to include a larger number of STM tags [88], [89].

### Generation of Signature Tagged Transposon Mutants

Random transposon mutagenesis of the infectious *B. burgdorferi* B31 clone 5A18NP1 was performed. Briefly, electrocompetent *B. burgdorferi* organisms were freshly prepared and transformed by electroporation with 5 µg of each plasmid (pGKT-STM1 through pGKT-STM11) using a modification [50] of previously described methods [38], [90]. The cultures were allowed to recover by overnight incubation in BSK-II medium without antibiotics, and transposon mutants were selected by subsurface plating on 0.7% solid BSK-II medium with 200 µg/ml of kanamycin and 40 µg/ml of gentamicin [91]. Colonies were selected and cultured in liquid BSK-II medium with the same antibiotics until mid-log phase prior to addition of 15% (v/v) glycerol and storage at -70°C. The transposon insertion site was determined using either inverse PCR or *E.coli* rescue of circularized *Hind*III fragments [38], but *E.coli* rescue was used routinely because of its higher efficiency. The exact transposon insertion site of each clone in the library was determined by dideoxynucleotide sequencing of the recovered plasmid using the clone sequencing primer (Table 4 in File S1). High-throughput identification of the replicon, insertion site, gene or intergenic region (IR) affected, transposon orientation, insertion ratio (no. nucleotides from gene start/total gene length) was accomplished using batch local BLAST analysis (Bioedit; http://bioedit.software.informer.com/) followed by semi-automated analysis with a lab-developed Excel/Visual Basic program. Genome coordinates and annotation for *B. burgdorferi* B31-MI as reported by Fraser et al. [4] were used, with the inclusion of more recent gene designations. The sequenced region included the STM tag, and the tag sequence was confirmed for each clone. The plasmid content of each clone was determined using a high-throughput Luminex-based method, as described by Norris et al. [45]. The *in vitro* growth phenotype of each STM mutant relative to the parent clone was determined subjectively under dark-field microscopy examination (scored as 1–4+) when the mutants were cultured for STM mouse inoculation. Properties of the transposon mutants are shown in Table 1 and Spreadsheet S1.

### Mouse Infection Studies

Pooled signature tagged transposon insertion mutants were screened for infectivity by needle inoculation in 4-week-old C3H/HeNHsd female mice. STM transposon mutants T01P01A11 and T02P01A01, with insertions in *bbe22* (*pncA*) and *bb0051* (conserved hypothetical integral membrane protein), respectively, were utilized as negative and positive controls. Eleven STM mutants (with tags 1 to 11) were cultured directly from frozen stocks in BSKII medium with kanamycin and gentamicin to mid-log phase. The concentration of organisms was determined by darkfield microscopy, and pools containing 1×10^6^/ml of each of the 11 clones were prepared. Six mice were inoculated with 0.1 ml (1×10^5^ organisms for each mutant) subcutaneously at the base of the tail as described previously [91]. Groups of 3 mice were sacrificed at days 14 and 28 post inoculation, and skin, ear, tibiotarsal joint, heart, and urinary bladder were collected. The tissue specimens were cultured in 6 ml BSK II medium containing kanamycin and gentamicin. Genomic DNA was also extracted directly from ∼2 mg tissue from the same site using the Qiagen DNeasy Blood & Tissue kit (QIAGEN, Valencia, CA, USA) and was resuspended in a final volume of 50 µl water.

Inoculation of *I. scapularis* nymphs by capillary feeding followed by mouse inoculation via tick feeding were carried out at the Tulane National Primate Center as described previously [50], [77]. Briefly, *B. burgdorferi* strains were cultured from frozen stocks in BSK II medium containing kanamycin (200 µg/ml) and gentamicin (50 µg/ml; Gibco) as appropriate to aid in the retention of lp25 and transposon-containing plasmids, respectively. Capillary feeding with cultures containing 3–5×10^7^ organisms per ml was utilized to inoculate *I. scapularis* ticks, and the ticks were rested for 21 to 25 days prior to a) assessment of *B. burgdorferi* infection by culture and direct immunofluorescence and b) feeding of 10–12 ticks each on three 8–10 week-old female C3H/HeN mice. Ticks were allowed to feed for repletion and collected for analysis by culture and immunofluorescence. Mice were bled and euthanized 4 weeks after tick feeding was completed. Serum was analyzed for anti-VlsE-C6 peptide reactivity by ELISA, and heart, bladder, ear and tibiotarsal joint tissue was placed in BSK-II medium for up to 8 weeks to assess the presence of *B. burgdorferi.*

### Semi-quantitative Detection of STM Mutants and Determination of Plasmid Content using Luminex Technology

STM screening was performed using a novel, high-throughput Luminex FlexMap™ procedure for semi-quantitative detection of STM mutants (Fig. 2). In this study, two types of samples were utilized for each tissue: *B. burgdorferi* cultured from the tissue specimen, and DNA extracted directly from the mouse tissue. For the cultures, 200 µl of culture (∼1×10^7^ total organisms/ml) was centrifuged, and the sedimented *B. burgdorferi* were resuspended in 20 µl water and heated to 100°C for 10 min; 2 µl of this preparation was used as the source of template DNA. For the DNA preparation, the DNA from a ∼2 mg tissue specimen was resuspended in 50 µl, and 2 µl of the DNA sample was utilized as template. A PCR reaction was performed to amplify 313 bp of the transposon sequence encompassing the STM tag-containing region, using the forward and reverse STM-PCR primers (Table 4 in File S1). The PCR conditions were 95°C for 15 min, followed by 30 cycles of 94°C (30 sec), 60°C (90 sec), and 72°C (60 sec) and 10 min extension time at 72°C. Five µl of the resultant PCR reactions were treated with 2 µl of a 1∶4 dilution of Exo/SAP (exonuclease I and shrimp alkaline phosphatase, U. S. Biologicals) to remove excess nucleotides and primers. The resulting preparations were heated to 80°C for 10 min to inactivate the Exo/SAP, and then subjected to 30 cycles of asymmetric primer extension (ASPE) in the presence of 5 µM each dATP, dTTP, dGTP, and biotin-dCTP using 500 nM of each primer specific for the STM tags (Table 4 in File S1). The 5′ ends of the ASPE primers contain an xTAG universal tag sequence and the 3′ end of the ASPE primers contain the STM tag. The resulting preparation was hybridized to the complementary anti-tag sequence coupled to a particular xMAP bead set and biotin labeled DNA was detected with PhycoLink Streptavidin-R-Phycoerythrin (SAPE). A ‘universal’ ASPE primer that hybridizes with a backbone region of the transposon and thus provides signal for the presence of any transposon-containing organisms was included in each reaction as a control (Table 4 in File S1). Detection and analysis was carried out using the Luminex 200TM System (Luminex Corporation, Austin, TX). Results were recorded as the median fluorescence intensity (MFI) for each sample. A detailed protocol of this procedure is available from the authors upon request.

## Supporting Information

File S1 **File S1 contains supporting information** **Tables 1** **–** **4** **and** **Figures 1** **–8.**
(PDF)

Spreadsheet S1 **Transposon insertion sites in 4,479 clones derived by electroporation of** ***B. burgdorferi*** **5A18NP1 with the** ***Himar1*** **suicide vector pGKT.**
(XLS)

Spreadsheet S2 **Mouse infectivity results obtained for 434** ***B. burgdorferi*** **STM mutants, using Luminex STM analysis.**
(XLS)
