# Supplementary material for: Analysis of an Ordered, Comprehensive STM Mutant Library in Infectious Borrelia burgdorferi: Insights into the Genes Required for Mouse Infectivity
Source: PLoS One. 2012 Oct 25;7(10):e47532. doi: 10.1371/journal.pone.0047532 (PMC3485029; doi:10.1371/journal.pone.0047532)

Supporting Information File S1

Analysis of an ordered, comprehensive STM mutant library in infectious *Borrelia burgdorferi*: insights into the genes required for mouse infectivity

Tao Lin, Lihui Gao, Chuhua Zhang, Evelyn Odeh, Mary B. Jacobs, Loïc Coutte, George Chaconas, Mario T. Philipp, and Steven J. Norris

DIRECTORY	**Pages**
**Table 1.** Transposon insertions obtained in *B. burgdorferi* genes with predicted functions.	2-24
**Table 2.** Genes with a single insertion in the last 10% of the reading frame.	25-26
**Table 3.** Summary of mouse infectivity results obtained for STM clones with insertions in cp26.	27-28
**Table 4.** Oligonucleotides used in this study.	29
**Figure 1.** Construction of signature-tagged suicide himar1 transposon vectors pGKTSTM1-pGKTSTM11 for transposon mutagenesis of infectious *Borrelia burgdorferi*.	30
**Figure 2.** Distribution of transposon insertions in *B. burgdorferi* genes.	31
**Figure 3.** Transposon insertion sites for each of the plasmids present in *B. burgdorferi* B31 5A18NP1.	32-52
**Figure 4.** Distribution of individual MFI values obtained with high, intermediate, and low infectivity STM transposon mutant clones.	53
**Figure 5.** MFI values obtained by Luminex-based STM analysis are reproducible.	54
**Figure 6.** Sampling error may contribute to the variability of Luminex-based MFI values obtained in mouse infectivity analyses.	54
**Figure 7.** Heat map representations of the mouse infectivity of transposon mutants in the genes of *B. burgdorferi* plasmids.	55-60
**Figure 8.** Heat map representations of the mouse infectivity of transposon mutants in genes by functional group.	61-66
**Spreadsheet S1.** Transposon insertion sites in 4,479 clones derived by electroporation of *B. burgdorferi* 5A18NP1 with the *Himar1* suicide vector pGKT. (Excel format)	Separate Excel File
**Spreadsheet S2.** Mouse infectivity results obtained for 434 *B. burgdorferi* STM mutants, using Luminex STM analysis. (Excel format)	Separate Excel File

Table 1. Transposon insertions obtained in *B. burgdorferi* genes with predicted functions.[a] Genes are arranged into broad (green) and narrow (blue) functional groups.

Predicted function	Replicon	Gene	Description	No. of genes	Gene(s) disrupted[b]	Disrupted only in last 10% of gene (Insertion ratio)	Proportion of genes disrupted[b]
**Amino acid biosynthesis**				**1**	**0**	**0**	**0.00**
**Serine family**	Chr	**BB601**	serine OH methyltransferase (glyA)				
**Biosynthesis of cofactors, prosthetic groups, and carriers**				**8**	**2**	**3**	**0.25**
**Folic acid**				**1**	**0**	**0**	**0.00**
	Chr	**BB026**	methylenetetrahydrofolate dehydrogenase (folD)				
**Heme and porphyrin**				**2**	**2**	**0**	**1.00**
	Chr	**BB197**	protoporphyrinogen oxidase		**Yes**		
	Chr	**BB656**	oxygen-independent coproporphyrinogen III oxidase		**Yes**		
**Menaquinone and ubiquinone**				**1**	**0**	**0**	**0.00**
	Chr	**BB314**	octaprenyl-diP synthase (ispB)				
**Pantothenate**				**1**	**0**	**1**	**0.00**
	Chr	**BB812**	pantothenate metabolism flavoprotein (dfp)			**Yes (1.00)**	
**Pyridoxine**				**1**	**0**	**0**	**0.00**
	Chr	**BB768**	pyridoxal kinase (pdxK)				
**Thiamine**				**1**	**0**	**1**	**0.00**
	Chr	**BB621**	4-methyl-5 (β-hydroxyethyl)-thiozole monophosphate biosynthesis protein (thiJ)			**Yes (0.92)**	
**Pyridine nucleotides**				**1**	**0**	**1**	**0.00**
	Chr	**BB522**	NH_3-dep NAD+ synthase			**Yes (0.99)**	
**Cell envelope**				**116**	**51**	**2**	**0.44**
**Membrane proteins, lipoproteins, and porins**				**44**	**28**	**0**	**0.64**
	Chr	**BB10**	surface-located membrane protein 1 (lmp1)				
	Chr	**BB108**	basic membrane protein				
	Chr	**BB158**	S2 protein		**Yes**		
	Chr	**BB167**	outer membrane protein (tpn50)		**Yes**		
	Chr	**BB319**	exported protein (tpn38b)		**Yes**		
	Chr	**BB347**	fibronectin/fibrinogen-binding protein		**Yes**		
	Chr	**BB365**	lipoprotein LA7		**Yes**		
	Chr	**BB382**	basic membrane protein B (bmpB)				

Predicted function	Replicon	Gene	Description	No. of genes	Gene(s) disrupted[b]	Disrupted only in last 10% of gene (Insertion ratio)	Proportion of genes disrupted[b]
	Chr	**BB383**	basic membrane protein A (bmpA)				
	Chr	**BB384**	basic membrane protein C (bmpC)		**Yes**		
	Chr	**BB385**	basic membrane protein D (bmpD)				
	Chr	**BB442**	inner membrane protein				
	Chr	**BB603**	membrane-associated protein p66				
	Chr	**BB735**	rare lipoprotein A (rlpA)		**Yes**		
	Chr	**BB753**	membrane spanning protein				
	Chr	**BB795**	outer membrane protein				
	cp09	**BBC06**	exported protein A (eppA)		**Yes**		
	cp26	**BBB07**	outer surface protein		**Yes**		
	cp26	**BBB19**	outer surface protein C (ospC)		**Yes**		
	lp25	**BBE09**	protein p23		**Yes**		
	lp28-1	**BBF01**	arthritis-related protein (arp)		**Yes**		
	lp28-1	**BBF22**	protein p23		**Yes**		
	lp28-1	**BBF32**	vls recombination cassette Vls3-16		**Yes**		
	lp28-3	**BBH20**	outer membrane porin (oms28)		**Yes**		
	lp28-3	**BBH21**	outer membrane porin (oms28)				
	lp36	**BBK52**	protein p23				
	lp36	**BBK53**	outer membrane protein				
	lp38	**BBJ09**	outer surface protein D (ospD)		**Yes**		
	lp38	**BBJ50**	outer membrane protein				
	lp38	**BBJ51**	vlsE paralog, authentic frameshift		**Yes**		
	lp38	**BBJ52**	vlsE paralog, authentic frameshift				
	lp54	**BBA03**	outer membrane protein		**Yes**		
	lp54	**BBA04**	S2 protein		**Yes**		
	lp54	**BBA05**	S1 protein		**Yes**		
	lp54	**BBA15**	outer surface protein A (ospA)				
	lp54	**BBA16**	outer surface protein B (ospB)		**Yes**		
	lp54	**BBA24**	decorin binding protein A (dbpA)		**Yes**		
	lp54	**BBA25**	decorin binding proteinB (dbpB)		**Yes**		
	lp54	**BBA36**	lipoprotein		**Yes**		

Predicted function	Replicon	Gene	Description	No. of genes	Gene(s) disrupted[b]	Disrupted only in last 10% of gene (Insertion ratio)	Proportion of genes disrupted[b]
	lp54	**BBA52**	outer membrane protein				
	lp54	**BBA59**	lipoprotein		**Yes**		
	lp54	**BBA60**	surface lipoprotein P27		**Yes**		
	lp54	**BBA62**	lipoprotein		**Yes**		
	lp54	**BBA74**	outer membrane porin (oms28)		**Yes**		
**Murein sacculus and peptidoglycan**				**20**	**2**	**2**	**0.10**
	Chr	**BB100**	glutamate racemase (murI)				
	Chr	**BB136**	penicillin-binding protein (pbp-1)				
	Chr	**BB160**	alanine racemase (alr)				
	Chr	**BB200**	D-alanine-D-alanine ligase (ddlA)				
	Chr	**BB201**	UDP-N-Acmuramoylalanyl-D-glutamate—2,6-diaminopimelate ligase (murE)				
	Chr	**BB303**	phospho-N-Acmuramoyl-pentapeptidetransferase (mraY)				
	Chr	**BB304**	UDP-N-Acmuramoylalanyl-D-glutamyl-2,6-diamino-pimelate-D-alanyl-D-alanine ligase (murF)				
	Chr	**BB472**	UDP-NAG 1-carboxy-vinyl transferase (murA)				
	Chr	**BB582**	CPDase		**Yes**		
	Chr	**BB585**	UDP-N-Acmuramoylalanine-D-glutamate ligase (murD)			**Yes (0.99)**	
	Chr	**BB598**	UDP-N-Acmuramate Dhase (murB)				
	Chr	**BB605**	serine-type D-Ala-D-Ala CPDase (dacA)		**Yes**		
	Chr	**BB625**	N-Acmuramoyl-L-alanine amidase				
	Chr	**BB715**	rod shape-determining protein (mreB-1)				
	Chr	**BB716**	rod shape-determining protein (mreC)				
	Chr	**BB718**	penicillin-binding protein (pbp-2)				
	Chr	**BB719**	rod shape-determining protein (mreB-2)				
	Chr	**BB732**	penicillin-binding protein (pbp-3)				
	Chr	**BB767**	UDP-N-Acglucosamine-N-Acmuramyl-(pentapeptide) pyrophosphoryl-undecaprenol NAG transferase (murG)			**Yes (0.97)**	
	Chr	**BB817**	UDP-N-Acmuramate-alanine ligase (murC)				

Predicted function	Replicon	Gene	Description	No. of genes	Gene(s) disrupted[b]	Disrupted only in last 10% of gene (Insertion ratio)	Proportion of genes disrupted[b]
**Surface polysaccharides, lipopolysaccharides and antigens**				**16**	**13**	**0**	**0.81**
	Chr	**BB572**	glycosyl transferase (lgtD)				
	Chr	**BB744**	antigen, p83/100		**Yes**		
	lp25	**BBE31**	antigen, P35				
	lp28-3	**BBH32**	antigen, P35		**Yes**		
	lp28-4	**BBI36**	antigen, P35				
	lp36	**BBK15**	antigen, P35, putative		**Yes**		
	lp36	**BBK32**	immunogenic protein P35		**Yes**		
	lp36	**BBK37**	immunogenic protein P37		**Yes**		
	lp36	**BBK45**	immunogenic protein P37		**Yes**		
	lp36	**BBK46**	immunogenic protein P37		**Yes**		
	lp36	**BBK48**	immunogenic protein P37		**Yes**		
	lp36	**BBK50**	immunogenic protein P37		**Yes**		
	lp38	**BBJ41**	antigen P35, putative		**Yes**		
	lp54	**BBA64**	antigen P35		**Yes**		
	lp54	**BBA66**	antigen P35		**Yes**		
	lp54	**BBA73**	antigen P35, putative		**Yes**		
**Flagella**				**36**	**8**	**0**	**0.22**
	Chr	**BB147**	flagellar filament 41 kDa core protein (flaB)				
	Chr	**BB149**	flgr hook-associated protein 2 (fliD2)				
	Chr	**BB180**	flgr protein				
	Chr	**BB181**	flgr hook-associated protein (flgK)				
	Chr	**BB182**	flgr hook-associated protein 3 (flgL)				
	Chr	**BB221**	flgr motor switch protein (fliG-1)		**Yes**		
	Chr	**BB270**	flgr-associated GTP-binding protein (flhF)				
	Chr	**BB271**	flgr biosynthesis protein (flhA)				
	Chr	**BB272**	flgr biosynthesis protein (flhB)				
	Chr	**BB273**	flgr biosynthesis protein (fliR)				
	Chr	**BB274**	flgr biosynthesis protein (fliQ)				
	Chr	**BB275**	flgr biosynthesis protein (fliP)				

Predicted function	Replicon	Gene	Description	No. of genes	Gene(s) disrupted[b]	Disrupted only in last 10% of gene (Insertion ratio)	Proportion of genes disrupted[b]
	Chr	**BB276**	flgr biosynthesis protein (fliZ)		**Yes**		
	Chr	**BB277**	flgr motor switch protein (fliN)				
	Chr	**BB278**	flgr motor switch protein (fliM)				
	Chr	**BB279**	flgr protein (fliL)				
	Chr	**BB280**	flgr motor rotation protein B (motB)				
	Chr	**BB281**	flgr motor rotation protein A (motA)				
	Chr	**BB282**	flgr protein (flbD)				
	Chr	**BB283**	flgr hook protein (flgE)				
	Chr	**BB284**	flgr hook assembly protein (flgD)				
	Chr	**BB285**	flgr protein (flbC)				
	Chr	**BB286**	flgr protein (flbB)				
	Chr	**BB287**	flgr protein (flbA)		**Yes**		
	Chr	**BB288**	flgr-specific ATPase (fliI)		**Yes**		
	Chr	**BB289**	flgr assembly protein (fliH)		**Yes**		
	Chr	**BB290**	flgr motor switch protein (fliG-2)				
	Chr	**BB291**	flgr basal-body rod protein (fliF)				
	Chr	**BB292**	flgr hook-basal body complex protein (fliE)				
	Chr	**BB293**	flgr basal-body rod protein (flgC)				
	Chr	**BB294**	flgr basal-body rod protein (flgB)		**Yes**		
	Chr	**BB550**	flgr protein (flaJ)				
	Chr	**BB668**	flgr filament outer layer protein (flaA)		**Yes**		
	Chr	**BB772**	flgr P-ring protein (flgI)		**Yes**		
	Chr	**BB774**	flgr basal-body rod protein (flgG)				
	Chr	**BB775**	flgr hook-basal body complex protein (flhO)				
**Cellular processes**				**66**	**27**	**2**	**0.41**
**General**				**2**	**0**	**0**	**0.00**
	Chr	**BB660**	GTP-binding protein (era)				
	Chr	**BB781**	GTP-binding protein (obg)				
**Chemotaxis**				**19**	**14**	**0**	**0.74**
	Chr	**BB040**	chemotaxis protein methyltransferase (cheR-1)				
	Chr	**BB312**	purine-B chemotaxis protein (cheW-1)				

Predicted function	Replicon	Gene	Description	No. of genes	Gene(s) disrupted[b]	Disrupted only in last 10% of gene (Insertion ratio)	Proportion of genes disrupted[b]
	Chr	**BB414**	chemotaxis protein methyltransferase (cheR-2)		**Yes**		
	Chr	**BB415**	protein-glutamate methylesterase (cheB-1)		**Yes**		
	Chr	**BB551**	chemotaxis response regulator (cheY-1)				
	Chr	**BB565**	purine-B chemotaxis protein (cheW-2)		**Yes**		
	Chr	**BB567**	chemotaxis histidine kinase (cheA-1)		**Yes**		
	Chr	**BB568**	protein-glutamate methylesterase (cheB-2)		**Yes**		
	Chr	**BB570**	chemotaxis response regulator (cheY-2)		**Yes**		
	Chr	**BB578**	MC protein (mcp-1)		**Yes**		
	Chr	**BB596**	MC protein (mcp-2)				
	Chr	**BB597**	MC protein (mcp-3)		**Yes**		
	Chr	**BB606**	chemoreceptor glutamine deamidase (cheD)		**Yes**		
	Chr	**BB669**	chemotaxis histidine kinase (cheA-2)		**Yes**		
	Chr	**BB670**	purine-B chemotaxis protein (cheW-3)		**Yes**		
	Chr	**BB671**	chemotaxis operon protein (cheX)		**Yes**		
	Chr	**BB672**	chemotaxis response regulator (cheY-3)				
	Chr	**BB680**	MC protein (mcp-4)		**Yes**		
	Chr	**BB681**	MC protein (mcp-5)		**Yes**		
**Cell division**				**13**	**2**	**1**	**0.15**
	Chr	**BB058**	cell division control protein 27				
	Chr	**BB195**	cell division control protein				
	Chr	**BB361**	cell division inhibitor		**Yes**		
	Chr	**BB299**	cell division protein (ftsZ)				
	Chr	**BB789**	cell division protein (ftsH)			**Yes (0.96)**	
	Chr	**BB076**	cell division protein				
	Chr	**BB257**	cell division protein				
	Chr	**BB300**	cell division protein (ftsA)				
	Chr	**BB302**	cell division protein (ftsW)				
	Chr	**BB301**	cell division protein (divIB)				
	Chr	**BB313**	cell division protein (ftsJ)				
	Chr	**BB434**	stage 0 sporulation protein J (spo0J)		**Yes**		
	lp28-2	**BBG08**	stage 0 sporulation protein J (spoOJ)				

Predicted function	Replicon	Gene	Description	No. of genes	Gene(s) disrupted[b]	Disrupted only in last 10% of gene (Insertion ratio)	Proportion of genes disrupted[b]
**Cell killing**				**5**	**3**	**0**	**0.60**
	Chr	**BB059**	hemolysin (tlyC)				
	Chr	**BB117**	hemolysin III (yplQ)		**Yes**		
	Chr	**BB143**	alpha-hemolysin (hlyA)				
	Chr	**BB202**	hemolysin		**Yes**		
	Chr	**BB506**	hemolysin (tlyA)		**Yes**		
**Chaperones**				**11**	**5**	**1**	**0.45**
	Chr	**BB264**	heat shock protein 70 (dnaK-1)		**Yes**		
	Chr	**BB295**	heat shock protein (hslU)		**Yes**		
	Chr	**BB296**	heat shock protein (hslV)		**Yes**		
	Chr	**BB517**	heat shock protein (dnaJ-1)				
	Chr	**BB518**	heat shock protein 70 (dnaK-2)				
	Chr	**BB519**	grpE protein (grpE)				
	Chr	**BB560**	heat shock protein 90 (htpG)		**Yes**		
	Chr	**BB602**	chaperonin			**Yes (1.00)**	
	Chr	**BB649**	heat shock protein (groEL)				
	Chr	**BB655**	heat shock protein (dnaJ-2)		**Yes**		
	Chr	**BB741**	chaperonin (groES)				
**Detoxification**				**3**	**1**	**0**	**0.33**
	Chr	**BB153**	superoxide dismutase (sodA)		**Yes**		
	Chr	**BB179**	thiophene and furan oxidation protein (thdF)				
	Chr	**BB690**	neutrophil activating protein (napA)				
**Protein and peptide secretion**				**11**	**1**	**0**	**0.09**
	Chr	**BB030**	signal peptidase I (lepB-1)				
	Chr	**BB031**	signal peptidase I (lepB-2)				
	Chr	**BB154**	preprotein translocase sub (secA)				
	Chr	**BB263**	signal peptidase I (lepB-3)		**Yes**		
	Chr	**BB362**	prolipoprotein diacylglyceryl transferase (lgt)				
	Chr	**BB395**	preprotein translocase sub (secE)				
	Chr	**BB498**	preprotein translocase sub (secY)				
	Chr	**BB610**	trigger factor (tig)				

Predicted function	Replicon	Gene	Description	No. of genes	Gene(s) disrupted[b]	Disrupted only in last 10% of gene (Insertion ratio)	Proportion of genes disrupted[b]
	Chr	**BB652**	protein-export membrane protein (secD)				
	Chr	**BB653**	protein-export membrane protein (secF)				
	Chr	**BB694**	signal recognition particleprotein (ffh)				
**Transformation**				**2**	**1**	**0**	**0.50**
	Chr	**BB591**	competence locus E		**Yes**		
	Chr	**BB798**	competence protein F				
**Central intermediary metabolism**				**11**	**5**	**0**	**0.45**
**General**				**3**	**2**	**0**	**0.67**
	Chr	**BB241**	glycerol kinase (glpK)		**Yes**		
	Chr	**BB243**	glycerol-3-P dehydrogenase, anaerobic (glpA)		**Yes**		
	Chr	**BB376**	SAM synthase (metK)				
**Amino sugars**				**2**	**0**	**0**	**0.00**
	Chr	**BB151**	N-acetylglucosamine-6-P-deAcase (nagA)				
	Chr	**BB152**	glucosamine-6-P-isomerase (nagB)				
**Degradation of polysaccharides**				**2**	**2**	**0**	**1.00**
	Chr	**BB002**	b-N-acetylhexosaminidase		**Yes**		
	Chr	**BB620**	b-glucosidase		**Yes**		
**Phosphorus compounds**				**1**	**0**	**0**	**0.00**
	Chr	**BB533**	phnP protein (phnP)				
**Polysaccharides (cytoplasmic)**				**3**	**1**	**0**	**0.33**
	Chr	**BB004**	phosphoglucomutase (femD)				
	Chr	**BB166**	4-a—glucanotransferase (malQ)		**Yes**		
	Chr	**BB835**	phosphomannomutase (cpsG)				
**Energy metabolism**				**26**	**2**	**1**	**0.08**
**Anaerobic**				**1**	**0**	**0**	**0.00**
	Chr	**BB728**	NADH oxidase, water-forming (nox)				
**Amino acid and amines**				**2**	**1**	**0**	**0.50**
	Chr	**BB841**	arginine deiminase (arcA)		**Yes**		
	Chr	**BB842**	ornithine carbamoyl transferase (arcB)				
**Anaerobic**				**2**	**0**	**0**	**0.00**
	Chr	**BB016**	glpE protein (glpE)				

Predicted function	Replicon	Gene	Description	No. of genes	Gene(s) disrupted[b]	Disrupted only in last 10% of gene (Insertion ratio)	Proportion of genes disrupted[b]
	Chr	**BB087**	L-lactate dehydrogenase (ldh)				
**ATP-proton motive force interconversion**				**6**	**0**	**0**	**0.00**
	Chr	**BB090**	V-type ATPase, sub K (atpK)				
	Chr	**BB091**	V-type ATPase, sub I (atpI)				
	Chr	**BB092**	V-type ATPase, sub D (atpD)				
	Chr	**BB093**	V-type ATPase, sub B (atpB)				
	Chr	**BB094**	V-type ATPase, sub A (atpA)				
	Chr	**BB096**	V-type ATPase, sub E (atpE)				
**Electron transport**				**2**	**0**	**0**	**0.00**
	Chr	**BB061**	thioredoxin (trxA)				
	Chr	**BB515**	thioredoxin reductase (trxB)				
**Fermentation**				**2**	**0**	**0**	**0.00**
	Chr	**BB589**	phosphate acetyltransferase (pta)				
	Chr	**BB622**	acetate kinase (ackA)				
**Glycolysis**				**11**	**1**	**1**	**0.09**
	Chr	**BB020**	pyroP-fructose 6-P1-PP transferaseb sub (pfpB)				
	Chr	**BB055**	triosePisomerase				
	Chr	**BB056**	phosphoglycerate kinase (pgk) {Bb}				
	Chr	**BB057**	glyceraldehyde 3-P dehydrogenase (gap)				
	Chr	**BB337**	enolase (eno)				
	Chr	**BB348**	pyruvate kinase (pyk)				
	Chr	**BB445**	fructose-bisPaldolase (fba)				
	Chr	**BB630**	1-phosphofructo kinase (fruK)		**Yes**		
	Chr	**BB658**	phosphoglycerate mutase (gpmA)				
	Chr	**BB727**	pyroP-fructose 6-P1-PP transferase (pfk)				
	Chr	**BB730**	glucose-6-P-isomerase (pgi)			**Yes (0.97)**	
**Pentose phosphate pathway**				**4**	**0**	**0**	**0.00**
	Chr	**BB222**	glucose-6-P1-dehydrogenase				
	Chr	**BB561**	phosphogluconate dehydrogenase (gnd)				
	Chr	**BB636**	glucose-6-P1-dehydrogenase (zwf)				

Predicted function	Replicon	Gene	Description	No. of genes	Gene(s) disrupted[b]	Disrupted only in last 10% of gene (Insertion ratio)	Proportion of genes disrupted[b]
	Chr	**BB657**	ribose 5-Pisomerase (rpi)				
**Sugars**				**5**	**2**	**0**	**0.40**
	Chr	**BB207**	UTP-glucose-1-Puridylyl transferase				
	Chr	**BB407**	mannose-6-isomerase (manA)				
	Chr	**BB444**	nucleotide sugar epimerase				
	Chr	**BB545**	xylulokinase (xylB)		**Yes**		
	Chr	**BB676**	phosphoglycolate P phosphatase (gph)		**Yes**		
**Fatty acid and phospholipid metabolism (General)**				**15**	**1**	**0**	**0.07**
	Chr	**BB037**	1-acyl-sn-glycerol-3-PAc transferase (plsC)				
	Chr	**BB109**	Ac-CoAC-Ac transferase (fadA)				
	Chr	**BB119**	phosphatidate cytidylylase (cdsA), AFS				
	Chr	**BB137**	long-chain-fatty-acid CoAligase				
	Chr	**BB249**	phosphatidyl transferase				
	Chr	**BB327**	glycerol-3-PO-acyl transferase				
	Chr	**BB368**	glycerol-3-P dehydrogenase, NAD (P)+ (gpsA)		**Yes**		
	Chr	**BB593**	long-chain-fatty-acid CoAligase				
	Chr	**BB683**	3-OH-3-methylglutaryl-CoA synthase				
	Chr	**BB685**	3-OH-3-methylglutaryl-CoA reductase (mvaA)				
	Chr	**BB686**	mevalonate pyroPDCase				
	Chr	**BB687**	phosphomevalonate kinase				
	Chr	**BB688**	melvalonate kinase				
	Chr	**BB704**	acyl carrier protein				
	Chr	**BB721**	CDP-diacylglycerol-glycerol-3-P 3-phosphatidyl transferase				
**Purines, pyrimidines, nucleosides, nucleotides**				**19**	**7**	**0**	**0.37**
**Nucleotide and nucleoside interconversion**				**6**	**1**	**0**	**0.17**
	Chr	**BB128**	cytidylate kinase (cmk-1)				
	Chr	**BB417**	adenylate kinase (adk)				
	Chr	**BB463**	nucleoside-dikinase (ndk)		**Yes**		
	Chr	**BB571**	uridylate kinase (smbA)				

Predicted function	Replicon	Gene	Description	No. of genes	Gene(s) disrupted[b]	Disrupted only in last 10% of gene (Insertion ratio)	Proportion of genes disrupted[b]
	Chr	**BB793**	thymidylate kinase (tmk)				
	Chr	**BB819**	cytidylate kinase (cmk-2)				
**Purine ribonucleotide biosynthesis**				**3**	**2**	**0**	**0.67**
	Chr	**BB544**	phosphoribosyl pyrophosphatase (prs)				
	cp26	**BBB17**	IMP dehydrogenase (guaB)		**Yes**		
	cp26	**BBB18**	GMP synthase (guaA)		**Yes**		
**Pyrimidine ribonucleotide biosynthesis**				**1**	**0**	**0**	**0.00**
	Chr	**BB575**	CTP synthase (pyrG)				
**Salvage of nucleosides and nucleotides**				**9**	**4**	**0**	**0.44**
	Chr	**BB015**	uridine kinase (udk)				
	Chr	**BB239**	deoxyguanosine/deoxyadenosine kinase (I) sub 2 (dck)				
	Chr	**BB375**	pfs protein (pfs-1)				
	Chr	**BB588**	pfs protein (pfs-2)		**Yes**		
	Chr	**BB618**	cytidine deaminase (cdd)		**Yes**		
	Chr	**BB777**	adenine phosphoribosyl transferase (apt)				
	Chr	**BB791**	thymidine kinase (tdk)				
	lp28-3	**BBH33**	adenine deaminase (adeC), paralog of BBK17		**Yes**		
	lp36	**BBK17**	adenine deaminase (adeC), paralog of BBH33		**Yes**		
**Regulatory functions (General)**				**15**	**6**	**0**	**0.40**
	Chr	**BB042**	P transport system regulatory protein (phoU)		**Yes**		
	Chr	**BB176**	methanol dehydrogenase regulator (moxR)				
	Chr	**BB184**	carbon storage regulator (csrA)				
	Chr	**BB198**	guanosine-3’,5’-bis (diP) 3’-pyrophosphohydrolase (spoT)		**Yes**		
	Chr	**BB379**	protein kinase C1 inhibitor (pkcI)				
	Chr	**BB416**	pheromone shutdown protein (traB)				
	Chr	**BB419**	response regulatory protein (rrp-1)		**Yes**		
	Chr	**BB420**	sensory transduction histidine kinase (hk-1)		**Yes**		
	Chr	**BB647**	ferric uptake regulation protein (fur)				

Predicted function	Replicon	Gene	Description	No. of genes	Gene(s) disrupted[b]	Disrupted only in last 10% of gene (Insertion ratio)	Proportion of genes disrupted[b]
	Chr	**BB693**	xylose operon regulatory protein (xylR-1)				
	Chr	**BB737**	histidine phospho kinase/phosphatase putative				
	Chr	**BB763**	response regulatory protein (rrp-2)				
	Chr	**BB764**	sensory transduction histidine kinase (hk-2)				
	Chr	**BB831**	xylose operon regulatory protein (xylR-2)		**Yes**		
	lp54	**BBA07**	chpAI protein		**Yes**		
**DNA metabolism**				**46**	**28**	**1**	**0.61**
**Degradation of DNA**				**2**	**2**	**0**	**1.00**
	Chr	**BB411**	endonuclease precursor (nucA)		**Yes**		
	Chr	**BB745**	endonuclease III (nth)		**Yes**		
**DNA replication**				**18**	**5**	**1**	**0.28**
	Chr	**BB014**	primosomal protein N (priA)		**Yes**		
	Chr	**BB035**	DNA topoisomerase IV (parC)				
	Chr	**BB036**	DNA topoisomerase IV (parE)				
	Chr	**BB111**	replicative DNA helicase (dnaB)			**Yes (1.00)**	
	Chr	**BB177**	glucose-inhibited div protein B (gidB)		**Yes**		
	Chr	**BB178**	glucose-inhibited div protein A (gidA)				
	Chr	**BB435**	DNA gyrase, sub A (gyrA)				
	Chr	**BB436**	DNA gyrase, sub B (gyrB)				
	Chr	**BB437**	chromosomal replication initiation protein (dnaA)				
	Chr	**BB438**	DNA polymerase III, subunit beta (dnaN)				
	Chr	**BB461**	DNA polymerase III, sub gamma/tau (dnaX)				
	Chr	**BB534**	exodeoxyribonuclease III (exoA)				
	Chr	**BB552**	DNA ligase (lig)				
	Chr	**BB579**	DNA polymerase III, sub alpha (dnaE)				
	Chr	**BB710**	DNA primase (DNA G)				
	Chr	**BB827**	ATP-dep helicase (hrpA)		**Yes**		
	Chr	**BB828**	DNA topoisomerase I (topA)		**Yes**		
	lp28-2	**BBG32**	replicative DNA helicase		**Yes**		
**DNA recombination and repair**				**21**	**16**	**0**	**0.76**
	Chr	**BB022**	Holliday junction DNA helicase (ruvB)		**Yes**		

Predicted function	Replicon	Gene	Description	No. of genes	Gene(s) disrupted[b]	Disrupted only in last 10% of gene (Insertion ratio)	Proportion of genes disrupted[b]
	Chr	**BB023**	Holliday junction DNA helicase (ruvA)		**Yes**		
	Chr	**BB053**	uracil DNA glycosylase (ung)		**Yes**		
	Chr	**BB098**	DNA mismatch repair protein		**Yes**		
	Chr	**BB114**	ss DNA-binding protein (ssb)				
	Chr	**BB131**	recA protein (recA)				
	Chr	**BB211**	DNA mismatch repair protein (mutL)		**Yes**		
	Chr	**BB254**	ss-DNA-specific exonuclease (recJ)		**Yes**		
	Chr	**BB344**	DNA helicase (uvrD)		**Yes**		
	Chr	**BB457**	excinuclease ABC, sub C (uvrC)		**Yes**		
	Chr	**BB581**	DNA recombinase (recG)		**Yes**		
	Chr	**BB607**	rep helicase, ss DNA-dep ATPase (rep)		**Yes**		
	Chr	**BB623**	transcription-repair coupling factor (mfd)		**Yes**		
	Chr	**BB632**	exodeoxyribonuclease V, a chain (recD)		**Yes**		
	Chr	**BB633**	exodeoxyribonuclease V, b chain (recB)				
	Chr	**BB634**	exodeoxyribonuclease V g chain (recC)				
	Chr	**BB797**	DNA mismatch repair protein (mutS)		**Yes**		
	Chr	**BB829**	exonuclease SbcD (sbcD)		**Yes**		
	Chr	**BB830**	exonuclease SbcC (sbcC)		**Yes**		
	Chr	**BB836**	excinuclease ABC, sub B (uvrB)		**Yes**		
	Chr	**BB837**	excinuclease ABC, sub A (uvrA)				
**DNA restriction and modification**				**5**	**5**	**0**	**1.00**
	Chr	**BB421**	DNA 3-methyladenine glycosylase		**Yes**		
	Chr	**BB422**	DNA 3-methyladenine glycosylase (mag)		**Yes**		
	lp25	**BBE02**	putative restriction-modification enzyme				
	lp25	**BBE29**	adenine specific DNA methyltransferase		**Yes**		
	lp28-3	**BBH09**	putative restriction-modification enzyme		**Yes**		
**Transcription**				**19**	**4**	**0**	**0.21**
**General**				**1**	**0**	**0**	**0.00**
	Chr	**BB052**	spoU protein (spoU)				
**Degradation of RNA**				**4**	**1**	**0**	**0.25**
	Chr	**BB046**	ribonuclease H (rnhB)		**Yes**		

Predicted function	Replicon	Gene	Description	No. of genes	Gene(s) disrupted[b]	Disrupted only in last 10% of gene (Insertion ratio)	Proportion of genes disrupted[b]
	Chr	**BB441**	ribonuclease Pprotein component (rnpA)				
	Chr	**BB705**	ribonuclease III (rnc)				
	Chr	**BB805**	polyribonucleotide nucleotidyl transferase (pnpA)				
**DNA-dependent RNA polymerase**				**6**	**2**	**0**	**0.33**
	Chr	**BB388**	DNA-directed RNA polymerase (rpoC)				
	Chr	**BB389**	DNA-directed RNA polymerase (rpoB)				
	Chr	**BB450**	RNA polymerase sigma-54 factor (ntrA)		**Yes**		
	Chr	**BB502**	DNA-directed RNA polymerase (rpoA)				
	Chr	**BB712**	RNA polymerase sigma-70 factor (rpoD)				
	Chr	**BB771**	RNA polymerase sigma factor (rpoS)		**Yes**		
**Transcription factors**				**6**	**0**	**0**	**0.00**
	Chr	**BB107**	N utilization substance protein B (nusB)				
	Chr	**BB132**	transcription elongation factor (greA)				
	Chr	**BB230**	transcription termination factor Rho (rho)				
	Chr	**BB355**	transcription factor				
	Chr	**BB394**	transcription antitermination factor (nusG)				
	Chr	**BB800**	N-utilization substance protein A (nusA)				
**RNA processing**				**2**	**1**	**0**	**0.50**
	Chr	**BB516**	RNA methyltransferase, TrmH family, group 3		**Yes**		
	Chr	**BB706**	polynucleotide adenylyl transferase (papS)				
**Translation**				**124**	**12**	**1**	**0.10**
**General**				**2**	**1**	**0**	**0.50**
	Chr	**BB590**	dimethyladenosine transferase (ksgA)		**Yes**		
	Chr	**BB802**	ribosome-B factor A (rbfA)				
**Amino acyl tRNA synthetases**				**20**	**0**	**0**	**0.00**
	Chr	**BB005**	tryptophanyl-tRNAsynthase (trsA)				
	Chr	**BB101**	asparaginyl-tRNAsynthase (asnS)				
	Chr	**BB135**	histidyl-tRNAsynthase (hisS)				
	Chr	**BB220**	alanyl-tRNAsynthase (alaS)				
	Chr	**BB226**	seryl-tRNAsynthase (serS)				
	Chr	**BB251**	leucyl-tRNAsynthase (leuS)				

Predicted function	Replicon	Gene	Description	No. of genes	Gene(s) disrupted[b]	Disrupted only in last 10% of gene (Insertion ratio)	Proportion of genes disrupted[b]
	Chr	**BB370**	tyrosyl-tRNAsynthase (tyrS)				
	Chr	**BB371**	glycyl-tRNAsynthase (glyS)				
	Chr	**BB372**	glutamyl-tRNAsynthase (gltX)				
	Chr	**BB402**	prolyl-tRNAsynthase (proS)				
	Chr	**BB446**	aspartyl-tRNAsynthase (aspS)				
	Chr	**BB513**	phenylalanyl-tRNAsynthase, a sub (pheS)				
	Chr	**BB514**	phenylalanyl-tRNAsynthase, b sub (pheT)				
	Chr	**BB587**	methionyl-tRNAsynthase (metG)				
	Chr	**BB594**	arginyl-tRNAsynthase (argS)				
	Chr	**BB599**	cysteinyl-tRNAsynthase (cysS)				
	Chr	**BB659**	lysyl-tRNAsynthase				
	Chr	**BB720**	threonyl-tRNAsynthase (thrZ)				
	Chr	**BB738**	valyl-tRNAsynthase (valS)				
	Chr	**BB833**	isoleucyl-tRNAsynthase (ileS)				
**Degradation of proteins, peptides, and glycopeptides**				**21**	**8**	**1**	**0.38**
	Chr	**BB067**	peptidase				
	Chr	**BB069**	aminopeptidase II				
	Chr	**BB104**	periplasmic serine protease DO (htrA)				
	Chr	**BB118**	RIP metalloprotease (rseP)		**Yes**		
	Chr	**BB203**	Lambda CII stability-governing protein (hflK)		**Yes**		
	Chr	**BB204**	Lambda CII stability-governing protein (hflC)		**Yes**		
	Chr	**BB248**	oligoendopeptidase F (pepF)				
	Chr	**BB253**	ATP-dep protease LA (lon-1)				
	Chr	**BB359**	carboxyl-terminal protease (ctp)			**Yes (1.00)**	
	Chr	**BB366**	aminopeptidase I (yscI)				
	Chr	**BB369**	ATP-dep Clp protease, sub A (clpA)				
	Chr	**BB430**	proline dipeptidase (pepQ)		**Yes**		
	Chr	**BB536**	zinc protease		**Yes**		
	Chr	**BB608**	aminoacyl-histidine dipeptidase (pepD)		**Yes**		
	Chr	**BB611**	ATP-dep Clp protease proteolytic component (clpP-1)				

Predicted function	Replicon	Gene	Description	No. of genes	Gene(s) disrupted[b]	Disrupted only in last 10% of gene (Insertion ratio)	Proportion of genes disrupted[b]
	Chr	**BB612**	ATP-dep Clp protease, sub X (clpX)				
	Chr	**BB613**	ATP-dep protease LA (lon-2)		**Yes**		
	Chr	**BB627**	vacuolar X-prolyl dipeptidyl aminopeptidase I (pepX)		**Yes**		
	Chr	**BB757**	ATP-dep Clp protease proteolytic component (clpP-2)				
	Chr	**BB769**	sialoglycoprotease (gcp)				
	Chr	**BB834**	ATP-dep Clp protease, sub C (clpC)				
**Nucleoproteins**				**1**	**0**	**0**	**0.00**
	Chr	**BB232**	hbbU protein				
**Protein modification**				**3**	**0**	**0**	**0.00**
	Chr	**BB065**	polypeptide deformylase (def)				
	Chr	**BB105**	methionine aminopeptidase (map)				
	Chr	**BB648**	serine/threonine kinase				
**Ribosomal proteins: synthesis and modification**				**53**	**0**	**0**	**0.00**
	Chr	**BB112**	ribosomal protein L9 (rplI)				
	Chr	**BB113**	ribosomal protein S18 (rpsR)				
	Chr	**BB115**	ribosomal protein S6 (rpsF)				
	Chr	**BB123**	ribosomal protein S2 (rpsB)				
	Chr	**BB127**	ribosomal protein S1 (rpsA)				
	Chr	**BB188**	ribosomal protein L20 (rplT)				
	Chr	**BB189**	ribosomal protein L35 (rpmI)				
	Chr	**BB229**	ribosomal protein L31 (rpmE)				
	Chr	**BB233**	ribosomal protein S20 (rpsT)				
	Chr	**BB256**	ribosomal protein S21 (rpsU)				
	Chr	**BB338**	ribosomal protein S9 (rpsI)				
	Chr	**BB339**	ribosomal protein L13 (rplM)				
	Chr	**BB350**	ribosomal protein L28 (rpmB)				
	Chr	**BB386**	ribosomal protein S7 (rpsG)				
	Chr	**BB387**	ribosomal protein S12 (rpsL)				
	Chr	**BB390**	ribosomal protein L7/L12 (rplL)				

Predicted function	Replicon	Gene	Description	No. of genes	Gene(s) disrupted[b]	Disrupted only in last 10% of gene (Insertion ratio)	Proportion of genes disrupted[b]
	Chr	**BB391**	ribosomal protein L10 (rplJ)				
	Chr	**BB392**	ribosomal protein L1 (rplA)				
	Chr	**BB393**	ribosomal protein L11 (rplK)				
	Chr	**BB396**	ribosomal protein L33 (rpmG)				
	Chr	**BB440**	ribosomal protein L34 (rpmH)				
	Chr	**BB477**	ribosomal protein S10 (rpsJ)				
	Chr	**BB478**	ribosomal protein L3				
	Chr	**BB479**	ribosomal protein L4 (rplD)				
	Chr	**BB480**	ribosomal protein L23 (rplW)				
	Chr	**BB481**	ribosomal protein L2 (rplB)				
	Chr	**BB482**	ribosomal protein S19 (rpsS)				
	Chr	**BB483**	ribosomal protein L22 (rplV)				
	Chr	**BB484**	ribosomal protein S3 (rpsC)				
	Chr	**BB485**	ribosomal protein L16 (rplP)				
	Chr	**BB486**	ribosomal protein L29 (rpmC)				
	Chr	**BB487**	ribosomal protein S17 (rpsQ)				
	Chr	**BB488**	ribosomal protein L14 (rplN)				
	Chr	**BB489**	ribosomal protein L24 (rplX)				
	Chr	**BB490**	ribosomal protein L5 (rplE)				
	Chr	**BB491**	ribosomal protein S14 (rpsN)				
	Chr	**BB492**	ribosomal protein S8 (rpsH)				
	Chr	**BB493**	ribosomal protein L6 (rplF)				
	Chr	**BB494**	ribosomal protein L18 (rplR)				
	Chr	**BB495**	ribosomal protein S5 (rpsE)				
	Chr	**BB496**	ribosomal protein L30 (rpmD)				
	Chr	**BB497**	ribosomal protein L15 (rplO)				
	Chr	**BB499**	ribosomal protein L36 (rpmJ)				
	Chr	**BB500**	ribosomal protein S13 (rpsM)				
	Chr	**BB501**	ribosomal protein S11 (rpsK)				
	Chr	**BB503**	ribosomal protein L17 (rplQ)				
	Chr	**BB615**	ribosomal protein S4 (rpsD)				

Predicted function	Replicon	Gene	Description	No. of genes	Gene(s) disrupted[b]	Disrupted only in last 10% of gene (Insertion ratio)	Proportion of genes disrupted[b]
	Chr	**BB695**	ribosomal protein S16 (rpsP)				
	Chr	**BB699**	ribosomal protein L19 (rplS)				
	Chr	**BB703**	ribosomal protein L32 (rpmF)				
	Chr	**BB778**	ribosomal protein L21 (rplU)				
	Chr	**BB780**	ribosomal protein L27 (rpmA)				
	Chr	**BB804**	ribosomal protein S15 (rpsO)				
**tRNA modification**				**12**	**2**	**0**	**0.17**
	Chr	**BB012**	pseudouridylate synthase I (hisT)				
	Chr	**BB021**	SAM: tRNAribosyl transferase-isomerase		**Yes**		
	Chr	**BB064**	methionyl-tRNAformyl transferase (fmt)				
	Chr	**BB084**	aminotransferase (nifS)				
	Chr	**BB341**	glu-tRNAamido transferase, sub B (gatB)				
	Chr	**BB342**	glu-tRNAamido transferase, sub A (gatA)				
	Chr	**BB343**	glu-tRNAamido transferase, sub C (gatC)				
	Chr	**BB698**	tRNA (guanine-N1)-M transferase (trmD)				
	Chr	**BB787**	peptidyl-tRNAhydrolase (pth)				
	Chr	**BB803**	tRNApseudouridine 55 synthase (truB)		**Yes**		
	Chr	**BB809**	tRNA-guanine transglycosylase (tgt)				
	Chr	**BB821**	2-methylthio-N6-isopentyladenosine tRNA modification enzyme (miaA)				
**Translation factors**				**12**	**1**	**0**	**0.08**
	Chr	**BB074**	peptide chain release factor 2 (prfB)				
	Chr	**BB088**	GTP-B membrane protein (lepA)		**Yes**		
	Chr	**BB121**	ribosome releasing factor (frr)				
	Chr	**BB122**	translation elongation factor TS (tsf)				
	Chr	**BB169**	translation initiation factor 1 (infA)				
	Chr	**BB190**	translation initiation factor 3 (infC)				
	Chr	**BB196**	peptide chain release factor 1 (prfA)				
	Chr	**BB214**	translation elongation factor P (efp)				
	Chr	**BB476**	translation elongation factor TU (tuf)				
	Chr	**BB540**	translation elongation factor G (fus-1)				

Predicted function	Replicon	Gene	Description	No. of genes	Gene(s) disrupted[b]	Disrupted only in last 10% of gene (Insertion ratio)	Proportion of genes disrupted[b]
	Chr	**BB691**	translation elongation factor G (fus-2)				
	Chr	**BB801**	translation initiation factor 2 (infB)				
**Transport and binding proteins**				**61**	**28**	**4**	**0.59**
**General**				**8**	**3**	**1**	**0.38**
	Chr	**BB080**	ABC transporter, ATP-binding protein				
	Chr	**BB269**	ATP-binding protein (ylxH-1)				
	Chr	**BB466**	ABC transporter, ATP-binding protein				
	Chr	**BB573**	ABC transporter, ATP-binding protein		**Yes**		
	Chr	**BB726**	ATP-binding protein (ylxH-2)		**Yes**		
	Chr	**BB742**	ABC transporter, ATP-binding protein			**Yes (0.95)**	
	Chr	**BB754**	ABC transporter, ATP-binding protein				
	lp38	**BBJ26**	ABC transporter, ATP-binding protein		**Yes**		
**Amino acids, peptide, and amines**				**20**	**7**	**1**	**0.35**
	Chr	**BB144**	Glycine/betaine/L-proline binding proteinABC transporter, binding protein (proX)			**Yes (0.99)**	
	Chr	**BB145**	Glycine/betaine/L-proline binding proteinABC transporter, permease protein (proW)		**Yes**		
	Chr	**BB146**	Glycine/betaine/L-proline binding proteinABC transporter,ATP-binding protein (proV)				
	Chr	**BB328**	oligopeptide ABC transporter, periplasmic binding protein (oppA-1)		**Yes**		
	Chr	**BB329**	oligopeptide ABC transporter, periplasmic binding protein (oppA-2)		**Yes**		
	Chr	**BB330**	oligopeptide ABC transporter, periplasmic binding protein (oppA-3)		**Yes**		
	Chr	**BB332**	oligopeptide ABC transporter, permease protein (oppB-1)				
	Chr	**BB333**	oligopeptide ABC transporter, permease protein (oppC-1)				
	Chr	**BB334**	oligopeptide ABC transporter, ATP-binding protein (oppD)				
	Chr	**BB335**	oligopeptide ABC transporter,ATP-binding protein (oppF)				
	Chr	**BB401**	glutamate transporter, putative				
	Chr	**BB639**	spermidine/putrescine ABC transporter, periplasmic binding protein (potD)				

Predicted function	Replicon	Gene	Description	No. of genes	Gene(s) disrupted[b]	Disrupted only in last 10% of gene (Insertion ratio)	Proportion of genes disrupted[b]
	Chr	**BB640**	spermidine/putrescine ABC transporter, permease protein (potC)				
	Chr	**BB641**	spermidine/putrescine ABC transporter, permease protein (potB)				
	Chr	**BB642**	spermidine/putrescine ABC transporter, ATP-binding protein (potA)				
	Chr	**BB729**	glutamate transporter (gltP)		**Yes**		
	Chr	**BB746**	oligopeptide ABC transporter, permease protein (oppC-2)				
	Chr	**BB747**	oligopeptide ABC transporter, permease protein (oppB-2)				
	cp26	**BBB16**	oligopeptide ABC transporter, periplasmic binding protein (oppA-4)		**Yes**		
	lp54	**BBA34**	oligopeptide ABC transporter, periplasmic binding protein (oppA-5)		**Yes**		
**Anions**				**4**	**0**	**0**	**0.00**
	Chr	**BB215**	phosphate ABC transporter, periplasmic P-binding protein (pstS)				
	Chr	**BB216**	phosphate ABC transporter, permease protein (pstC)				
	Chr	**BB217**	phosphate ABC transporter, permease protein (pstA)				
	Chr	**BB218**	phosphate ABC transporter, ATP-binding protein (pstB)				
**Carbohydrates, organic alcohols, and acids**				**19**	**12**	**1**	**0.63**
	Chr	**BB116**	PTS system, mal/glu-specific IIABC (malX)		**Yes**		
	Chr	**BB240**	glycerol uptake facilitator (glpF)		**Yes**		
	Chr	**BB318**	methylgalactosideABC transporter, ATP-binding protein (mglA)		**Yes**		
	Chr	**BB408**	PTS system, fru-specific IIABC (fruA-1)		**Yes**		
	Chr	**BB448**	phosphocarrier protein HPr (ptsH-1)		**Yes**		
	Chr	**BB557**	phosphocarrier protein HPr (ptsH-2)				
	Chr	**BB558**	phosphoenolpyruvate-protein Ppase (ptsI)				
	Chr	**BB559**	PTS system, glu-specific IIA (crr)				
	Chr	**BB604**	L-lactate D605 permease (lctP)		**Yes**		
	Chr	**BB629**	PTS system, fru-specific IIABC (fruA-2)		**Yes**		

Predicted function	Replicon	Gene	Description	No. of genes	Gene(s) disrupted[b]	Disrupted only in last 10% of gene (Insertion ratio)	Proportion of genes disrupted[b]
	Chr	**BB645**	PTS system, glu-specific IIBC (ptsG)		**Yes**		
	Chr	**BB677**	ribose-galactose ABC transporter,ATP-binding protein (mglA)				
	Chr	**BB678**	ribose-galactose ABC transporter, permease protein (rbsC-1)				
	Chr	**BB679**	ribose-galactose ABC transporter, permease protein (rbsC-2)				
	Chr	**BB814**	pantothenate permease (panF)			**Yes (0.99)**	
	cp26	**BBB04**	PTS system IIC chitibiose transporter protein chbC		**Yes**		
	cp26	**BBB05**	PTS system IIA chitibiose transporter protein chbA		**Yes**		
	cp26	**BBB06**	PTS system IIB chitibiose transporter protein chbB		**Yes**		
	cp26	**BBB29**	PTS system, glu-specific IIBC		**Yes**		
**Cations**				**6**	**3**	**1**	**0.50**
	Chr	**BB164**	Na+/Ca+ exchange protein			**Yes (0.90)**	
	Chr	**BB380**	Mg2+ transport protein (mgtE)				
	Chr	**BB447**	Na+/H+ antiporter (napA)		**Yes**		
	Chr	**BB637**	Na+/H+ antiporter (nhaC-1)		**Yes**		
	Chr	**BB638**	Na+/H+ antiporter (nhaC-2)		**Yes**		
	Chr	**BB724**	K+ transport protein (ntpJ)				
**Other**				**4**	**3**	**0**	**0.75**
	Chr	**BB140**	Borrelia efflux system protein B (besB)		**Yes**		
	Chr	**BB141**	Borrelia efflux system protein A (besA)		**Yes**		
	Chr	**BB142**	Borrelia efflux system protein C (besC)		**Yes**		
	Chr	**BB451**	chromate transport protein				
**Other categories**				**18**	**9**	**0**	**0.50**
**Adaptations and atypical conditions**				**4**	**0**	**0**	**0.00**
	Chr	**BB237**	acid-inducible protein (act206)				
	Chr	**BB785**	stage V sporulation protein G				
	Chr	**BB786**	general stress protein (ctc)				
	Chr	**BB810**	virulence factor mviN protein (mviN)				
**Colicin-related functions**				**2**	**0**	**0**	**0.00**
	Chr	**BB546**	outer membrane integrity (tolA)				

Predicted function	Replicon	Gene	Description	No. of genes	Gene(s) disrupted[b]	Disrupted only in last 10% of gene (Insertion ratio)	Proportion of genes disrupted[b]
	Chr	**BB766**	colicin V production protein				
**Drug and analog sensitivity**				**4**	**1**	**0**	**0.25**
	Chr	**BB258**	bacitracin resistance protein (bacA)				
	Chr	**BB586**	femA protein (femA)				
	lp25	**BBE22**	pyrazinamidase/nicotinamidase (pncA)		**Yes**		
	lp28-4	**BBI26**	multidrug-efflux transporter				
**Transposon-related functions**				**8**	**8**	**0**	**1.00**
	lp17	**BBD20**	transposon-like protein, authentic frameshift		**Yes**		
	lp17	**BBD23**	transposon-like protein, authentic frameshift		**Yes**		
	lp28-1	**BBF18**	transposon-like protein		**Yes**		
	lp28-1	**BBF19**	transposon-like protein, authentic frameshift		**Yes**		
	lp28-2	**BBG05**	transposon-like protein		**Yes**		
	lp28-3	**BBH40**	transposon-like protein,		**Yes**		
	lp36	**BBK25**	transposon-like protein, authentic frameshift		**Yes**		
	lp38	**BBJ05**	transposon-like protein, pseudogene		**Yes**		
**Annotated, but unknown function**				**19**	**8**	**0**	**0.42**
	Chr	**BB033**	small protein (smpB)				
	Chr	**BB045**	P115 protein		**Yes**		
	Chr	**BB168**	dnaK suppressor, putative				
	Chr	**BB219**	gufA protein				
	Chr	**BB250**	dedA protein (dedA)				
	Chr	**BB297**	smg protein		**Yes**		
	Chr	**BB336**	P26		**Yes**		
	Chr	**BB363**	periplasmic protein		**Yes**		
	Chr	**BB443**	spoIIIJ-associated protein (jag)				
	Chr	**BB454**	lipopolysaccharide biosynthesis-related protein				
	Chr	**BB508**	GTP-binding protein				
	Chr	**BB524**	inositol monophosphate phosphatase		**Yes**		
	Chr	**BB528**	aldose reductase, putative		**Yes**		
	Chr	**BB684**	carotenoid biosynthesis protein, putative				
	Chr	**BB702**	lipopolysaccharide biosynthesis-related protein				

Predicted function	Replicon	Gene	Description	No. of genes	Gene(s) disrupted[b]	Disrupted only in last 10% of gene (Insertion ratio)	Proportion of genes disrupted[b]
	cp09	**BBC09**	rev protein (rev)		**Yes**		
	cp09	**BBC10**	rev protein (rev)		**Yes**		
	lp28-4	**BBI06**	pfs protein (pfs)				
	lp54	**BBA76**	thy1 protein (thy1)				
**Total**				564	190	14	**0.34**

a Functional groups and assigned genes adapted from Fraser et al. (4).

b Excluding genes disrupted only in the last 10% of the open reading frame

Table 2. Genes with a single insertion in the last 10% of the reading frame.										
**Replicon**	**Gene**	**Name of clone**	**Insertion Site**	**Tn Or.**	**Inserts per kb DNA**	**Locus size (bp)**	**Gene Or.**	**Insertion Ratio**	**Description**	**Common Name**
Chrom	BB0085	T11TC194	81399	Reverse	2.35	426	Forward	0.98	hypothetical protein	
Chrom	BB0089	T09TC444	85935	Reverse	1.03	975	Reverse	0.94	hypothetical protein	
Chrom	BB0111	T06TC366	109676	Reverse	0.73	1368	Reverse	1.00	replicative DNA helicase	dnaB
Chrom	BB0144	T06TC417	144595	Reverse	1.15	873	Reverse	0.99	glycine betaine, L-proline ABC transporter, glycine/betaine/L-proline-binding protein	proX
Chrom	BB0164	T11P02D04	164997	Reverse	0.99	1014	Reverse	0.91	Na+/Ca+ exchange protein, putative	
Chrom	BB0245	T08TC689	250672	Reverse	1.80	555	Reverse	0.97	hypothetical protein	
Chrom	BB0256	T11TC311	267273	Forward	4.76	210	Forward	0.99	ribosomal protein S21	rpsU
Chrom	BB0352	T03TC295	362288	Reverse	0.88	1134	Forward	0.97	hypothetical protein	
Chrom	BB0359	T06TC006	366965	Forward	0.70	1428	Reverse	1.00	carboxyl-terminal protease	ctp
Chrom	BB0374	T08P01E10	385422	Forward	0.88	1140	Forward	1.00	hypothetical protein	
Chrom	BB0433	T04TC385	450979	Reverse	10.10	99	Reverse	0.93	hypothetical protein	
Chrom	BB0522	T04TC403	533599	Forward	1.49	669	Forward	0.99	NH(3)-dependent NAD+ synthetase	
Chrom	BB0527	T07TC091	538284	Forward	1.27	789	Forward	0.97	conserved hypothetical protein	
Chrom	BB0542	T05TC330	553009	Forward	1.73	579	Forward	0.98	hypothetical protein	
Chrom	BB0545	T03TC312	556559	Forward	0.73	1365	Forward	0.98	xylulokinase	xylB
Chrom	BB0585	T08TC446	603714	Reverse	0.74	1356	Forward	0.99	UDP-N-acetylmuramoylalanine--D-glutamate ligase	murD
Chrom	BB0602	T05TC458	627036	Reverse	1.33	753	Forward	1.00	DnaJ domain containing protein	
Chrom	BB0621	T05TC615	649064	Reverse	1.80	555	Forward	0.92	4-methyl-5(b-hydroxyethyl)-thiazole monophosphate biosynthesis protein	thiJ
Chrom	BB0631	T08P01B02	662623	Reverse	3.17	315	Reverse	0.94	hypothetical protein	
Chrom	BB0663	T08TC335	702261	Reverse	2.22	450	Forward	0.97	hypothetical protein	
Chrom	BB0689	T06TC547	730665	Reverse	2.14	468	Reverse	0.93	hypothetical protein	
Chrom	BB0730	T09TC447	769586	Reverse	0.63	1599	Reverse	0.98	glucose-6-phosphate isomerase	pgi
Chrom	BB0733	T11P01A09	775396		1.20	831	Forward	0.92	hypothetical protein	
Chrom	BB0742	T05TC553	784934	Reverse	0.59	1698	Forward	0.95	ABC transporter, ATP-binding protein	
Chrom	BB0767	T04TC203	808494	Reverse	0.92	1092	Reverse	0.97	UDP-N-acetylglucosamine--N-acetylmuramyl-(pentapeptide) pyrophosphoryl-undecaprenol N-acetylglucosamine transferase	murG
Chrom	BB0789	T11TC038	827548	Forward	0.52	1920	Forward	0.96	cell division protein	ftsH
Chrom	BB0812	T09TC008	858940	Reverse	0.85	1173	Reverse	1.00	pantothenate metabolism flavoprotein	dfp
Chrom	BB0814	T06TC081	861759	Reverse	0.75	1335	Forward	0.99	pantothenate permease	panF
Chrom	BB0823	T04TC350	867778	Reverse	2.69	372	Reverse	0.91	hypothetical protein	
cp26	BBB03	T08TC298	922	Reverse	0.74	1350	Reverse	0.94	hypothetical protein	
cp32-1, cp32-3 or cp32-8	BBP11	T10P01C12	7729	Reverse	0.90	1113	Forward	0.92	hypothetical protein; identical sequence, position in all three plasmids	
cp32-3	BBS21	T09TC133	14312	Forward	1.88	531	Forward	1.00	conserved hypothetical protein	
cp32-3	BBS27	T08TC557	16878	Forward	3.14	318	Forward	0.92	hypothetical protein	
cp32-4	BBR09	T11TC449	6147	Forward	2.56	390	Forward	0.95	conserved hypothetical protein	
cp32-4	BBR40	T09TC232	25967	Reverse	9.52	105	Forward	0.98	erpH protein	erpH
cp32-6	BBM09	T11TC021	6144	Reverse	2.56	390	Forward	0.95	conserved hypothetical protein	
cp32-7	BBO13	T05TC227	8696	Forward	2.19	456	Forward	0.93	hypothetical protein	
cp32-7	BBO41	T11TC493	28012	Reverse	8.77	114	Reverse	0.93	hypothetical protein	
cp32-9	BBN05	T11P02B02	3940	Reverse	1.64	611	Forward	0.98	hypothetical protein, paralogous family 148, authentic frameshift	
lp17	BBD15	T08TC225	9614	Forward	2.36	423	Reverse	0.95	hypothetical protein, paralogous family 85	

Table 2, ctd. Genes with a single insertion in last 10% of reading frame.										
**Replicon**	**Gene**	**Name of clone**	**Insertion Site**	**Tn Or.**	**Inserts per kb DNA**	**Locus size (bp)**	**Gene Or.**	**Insertion Ratio**	**Description**	**Common Name**
lp21	BBU03	T09TC289	1239	Reverse	8.33	120	Reverse	0.99	hypothetical protein	
lp25	BBE04.1	T04TC095	5726	Forward	2.77	361	Forward	0.97	protein p23, pseudogene	
lp25	BBE18	T04TC328	11521	Reverse	1.72	582	Reverse	0.96	conserved hypothetical protein	
lp28-1	BBF07	T11TC414	3411	Reverse	8.33	120	Reverse	0.99	hypothetical protein	
lp28-1	BBF27	T10TC112	15769	Forward	5.85	171	Reverse	0.92	hypothetical protein	
lp28-2	BBG33	T10TC240	28777	Reverse	1.25	801	Forward	0.93	conserved hypothetical protein	
lp28-3	BBH09.1	T11TC342	7816	Reverse	3.55	282	Reverse	0.98	conserved hypothetical protein, pseudogene	
lp36	BBK42	T04TC031	26586	Reverse	4.57	219	Reverse	1.00	hypothetical protein	
lp36	BBK42.1	T10TC040	27077	Forward	6.02	166	Forward	0.98	conserved hypothetical protein	
lp38	BBJ51	T03TC222	37384	Forward	0.87	1144	Reverse	1.00	vlsE paralog, authentic frameshift	
lp54	BBA35	T10TC201	23282	Reverse	9.01	111	Reverse	0.99	hypothetical protein	

Table 3. Summary of mouse infectivity results obtained for *B. burgdorferi* STM clones with insertions in cp26 genes.

								No. Tissues Positive/Total Examined[a]											
								2 Weeks Post Inoculation						4 Weeks Post Inoculation					
Clone Name	Plasmid Missing	Replicon	Gene	Insertion Ratio	Description	Common Name	STM Set	Bladder	Ear	Heart	Joint	Inoc. Site	All Sites	Bladder	Ear	Heart	Joint	Inoc. Site	All Sites
T09TC018	None	cp26	BBB01	0.11	Acylphosphatase		36	3/3	3/3	3/3	3/3	3/3	15/15	2/3	3/3	3/3	2/3	3/3	13/15
3TC076	lp5, lp21	cp26	BBB02	0.44	Conserved hypothetical protein		36	0/3	0/3	0/3	1/3	2/3	3/15	0/3	0/3	0/3	0/3	0/3	0/15
T08TC298	cp9, lp5	cp26	BBB03	0.94	Telomere resolvase	resT	37	3/3	1/3	1/3	3/3	3/3	11/15	2/3	1/3	0/3	3/3	3/3	9/15
T09TC043	lp5	cp26	BBB04	0.20	PTS system IIC chitibiose transporter protein	chbC	42	2/3	2/3	3/3	3/3	3/3	13/15	3/3	3/3	1/3	3/3	3/3	13/15
T08P01C04	lp21, lp5	cp26	BBB04	0.23	PTS system IIC chitibiose transporter protein	chbC	9	3/3	3/3	3/3	3/3	ND	12/12	3/3	2/3	0/3	3/3	ND	8/12
T05TC295	cp9, lp5	cp26	BBB05	0.42	PTS system IIA chitibiose transporter protein	chbA	37	3/3	3/3	1/3	3/3	3/3	13/15	0/3	3/3	0/3	3/3	1/3	7/15
T08TC580	lp5	cp26	BBB06	0.13	PTS system IIB chitibiose transporter protein	chbB	39	0/3	0/3	1/3	0/3	1/3	2/15	0/3	1/3	1/3	0/3	0/3	2/15
T02P01C06	lp5	cp26	BBB06	0.86	PTS system IIB chitibiose transporter protein	chbB	2	0/3	0/3	0/3	0/3	ND	0/12	ND	ND	ND	ND	ND	ND
T07TC077	lp5	cp26	BBB07	0.11	Putative alpha3-beta1 integrin-binding outer surface protein		37	0/3	0/3	1/3	1/3	3/3	5/15	0/3	1/3	1/3	0/3	0/3	2/15
T11TC010	lp5	cp26	BBB08	0.10	Lipoprotein, putative		24	3/3	3/3	2/3	3/3	3/3	14/15	1/3	2/3	1/3	3/3	3/3	10/15
T11TC010	lp5	cp26	BBB08	0.10	Lipoprotein, putative		36	3/3	2/3	1/3	3/3	3/3	12/15	0/3	1/3	1/3	2/3	2/3	6/15
T08TC506	None	cp26	BBB09	0.09	Lipoprotein, putative		26	0/3	0/3	0/3	0/3	0/3	0/15	0/3	0/3	0/3	0/3	0/3	0/15
T03TC124	None	cp26	BBB09	0.28	Lipoprotein, putative		37	0/3	0/3	0/3	0/3	1/3	1/15	0/3	0/3	0/3	1/3	0/3	1/15
T03TC311	lp5	cp26	BBB12	0.27	Conserved hypothetical protein, protein family 32	PF32	35	0/3	0/3	0/3	0/3	0/3	0/15	0/3	0/3	0/3	0/3	0/3	0/15
T10TC205	lp5	cp26	BBB13	0.46	Putative plasmid partition protein, protein family 49	PF49	37	0/3	0/3	0/3	0/3	0/3	0/15	0/3	0/3	0/3	0/3	0/3	0/15
T06TC154	lp5	cp26	BBB14	0.43	Conserved hypothetical protein		33	0/3	0/3	0/3	0/3	0/3	0/15	0/3	0/3	0/3	0/3	1/3	1/15
T07TC044	None	cp26	BBB14	0.11	Conserved hypothetical protein		25	0/3	1/3	1/3	0/3	0/3	2/15	0/3	0/3	0/3	0/3	0/3	0/15
T07TC044	None	cp26	BBB14	0.11	Conserved hypothetical protein		36	1/3	0/3	0/3	1/3	1/3	3/15	0/3	0/3	0/3	0/3	0/3	0/15
T11TC530	None	cp26	BBB15	0.06	hypothetical protein		37	3/3	2/3	3/3	3/3	3/3	14/15	2/3	3/3	3/3	3/3	2/3	13/15
T05P1D02	None	cp26	BBB16	0.06	Oligopeptide ABC transporter, periplasmic binding protein	oppAIV	7	1/3	1/3	2/3	2/3	0/3	6/15	2/3	3/3	2/3	3/3	NA	10/12
T10P01A05	None	cp26	BBB16	0.17	Oligopeptide ABC transporter, periplasmic binding protein	oppAIV	2	0/3	0/3	0/3	0/3	0/3	0/15	ND	ND	ND	ND	ND	ND
T06TC037	None	cp26	BBB16	0.45	Oligopeptide ABC transporter, periplasmic binding protein	oppAIV	27	3/3	0/3	2/3	3/3	2/3	10/15	0/3	0/3	0/3	3/3	3/3	6/15
T06TC037	None	cp26	BBB16	0.17	Oligopeptide ABC transporter, periplasmic binding protein	oppAIV	36	3/3	0/3	0/3	3/3	3/3	9/15	3/3	2/3	2/3	3/3	3/3	13/15
T06TC118	cp32-3, lp5	cp26	BBB17	0.41	Inosine-5-monophosphate dehydrogenase	guaB	37	0/3	0/3	1/3	1/3	2/3	4/15	0/3	1/3	1/3	1/3	1/3	4/15
T04TC173	None	cp26	BBB18	0.18	GMP synthase	guaA	37	0/3	0/3	1/3	0/3	0/3	1/15	0/3	0/3	0/3	0/3	0/3	0/15
T08TC072	lp5	cp26	BBB19	0.07	Outer surface protein C	ospC	19	0/3	0/3	1/3	0/3	0/3	1/15	0/3	1/3	0/3	0/3	0/3	1/15
T08TC175	None	cp26	BBB19	0.51	Outer surface protein C	ospC	36	0/3	0/3	0/3	0/3	0/3	0/15	0/3	0/3	0/3	0/3	0/3	0/15
T07TC104	lp5	cp26	BBB20	0.41	Conserved hypothetical protein	CHP	35	2/3	2/3	3/3	3/3	3/3	13/15	2/3	3/3	1/3	2/3	3/3	11/15

Table 3, ctd. Summary of mouse infectivity results obtained for *B. burgdorferi* STM clones with insertions in cp26 genes.

								No. Tissues Positive/Total Examined[a]											
								2 Weeks Post Inoculation						4 Weeks Post Inoculation					
**Clone Name**	**Plasmid Missing**	**Replicon**	**Gene**	**Insertion Ratio**	**Description**	**Common Name**	**STM Set**	**Bladder**	**Ear**	**Heart**	**Joint**	**Inoc. Site**	**All Sites**	**Bladder**	**Ear**	**Heart**	**Joint**	**Inoc. Site**	**All Sites**
T08TC648	lp5	cp26	BBB21	0.76	Conserved hypothetical protein	CHP	50	3/3	1/3	3/3	3/3	3/3	13/15	3/3	3/3	2/3	3/3	3/3	14/15
T07TC086	cp9, lp5	cp26	BBB22	0.15	Purine permease G1	pbuG1	34	0/3	0/3	0/3	0/3	0/3	0/15	1/3	3/3	1/3	1/3	3/3	9/15
T06TC015	lp5	cp26	BBB22	0.44	Purine permease G1	pbuG1	32	1/3	0/3	0/3	1/3	1/3	3/15	0/3	0/3	0/3	0/3	1/3	1/15
T03TC156	lp5, cp9	cp26	BBB23	0.19	Purine permease G2	pbuG2	34	0/3	0/3	0/3	0/3	0/3	0/15	0/3	1/3	1/3	1/3	0/3	3/15
T05TC179	lp21, cp32-3	cp26	BBB24	0.15	Conserved hypothetical protein	CHP	36	2/3	3/3	2/3	3/3	3/3	13/15	2/3	3/3	3/3	3/3	3/3	14/15
T03P01A07	None	cp26	BBB25	0.5	Conserved hypothetical protein	CHP	3	2/2	0/2	0/2	1/2	ND	3/12	ND	ND	ND	ND	ND	ND
T10TC048	cp9, lp5, lp28-2	cp26	BBB25	0.67	Conserved hypothetical protein	CHP	36	1/3	1/3	1/3	3/3	3/3	9/15	1/3	0/3	0/3	3/3	1/3	5/15
T09TC218	lp5	cp26	BBB27	0.1	Lipoprotein, putative	LP	37	1/3	1/3	2/3	3/3	3/3	10/15	0/3	0/3	0/3	1/3	2/3	3/15
T09TC218	lp5	cp26	BBB27	0.1	Lipoprotein, putative	LP	19	3/3	0/3	2/3	2/3	3/3	10/15	1/3	1/3	2/3	3/3	3/3	10/15
T06TC312	cp9, lp5	cp26	BBB28	0.27	Conserved hypothetical protein (Borrelia only); putative ankyrin repeat protein	CHP	34	2/3	0/3	0/3	2/3	3/3	7/15	1/3	2/3	2/3	2/3	3/3	10/15
T04TC008	None	cp26	BBB29	0.17	PTS system, IIBC component	malX	7	0/3	0/3	0/3	1/3	ND	1/12	0/3	1/3	1/3	1/3	NA	3/12
T04TC008	None	cp26	BBB29	0.17	PTS system, IIBC component	malX	36	0/3	2/3	3/3	3/3	3/3	11/15	0/3	1/3	3/3	2/3	2/3	8/15

a Results obtained by direct extraction of DNA from tissue are shown. Tissue samples were considered positive if MFI > 100.

Table 4. Oligonucleotides used in this study. Underlined regions indicate 7-bp signature tags. See Materials and Methods for details regarding STM plasmid construction and Luminex detection methods.

Name	Nucleotide Sequences
Tag 1 Construct Oligos	5′-TCGGATCCCTGAACGTTTAAGCGCGGTACGGTACC-3′<br>3′-CGAGCCTAGGGACTTGCAAATTCGCGCCATGCCATGGAAT-5′
Tag 2 Construct Oligos	5′-TCGATCCCAGGCTATTTAAGCGCGGTACCTCGAGGCATGCGGTAC-3′<br>3′-CGAGCCTAGGTCCGATAAATTCGCGCCATGGAGCTCCGTACGC-5′
Tag 3 Construct Oligos	5′-GATCCCGAGACTTTTAAGCGCGGTACCTCGAGGCATG-3′<br>3′-GGCTCTGAAAATTCGCGCCATGGAGCTCC-5′
Tag 4 Construct Oligos	5′-GATCCCCACGTTATTAAGCGCGGTACCTCGAGGCATG-3′<br>3′-GGGTGCAATAATTCGCGCCATGGAGCTCC-5′
Tag 5 Construct Oligos	5′-GATCCCTACCAGTTTAAGCGCGGTACCTCGAGGCATG-3′<br>3′-GGATGGTCAAATTCGCGCCATGGAGCTCC-5′
Tag 6 Construct Oligos	5′-GATCCCAACATGCTTAAGCGCGGTACCTCGAGGCATG-3′<br>3′-GGTTGTACGAATTCGCGCCATGGAGCTCC-5′
Tag 7 Construct Oligos	5′-GATCCCGGTTACATTAAGCGCGGTACCTCGAGGCATG-3′<br>3′-GGCCAATGTAATTCGCGCCATGGAGCTCC-5′
Tag 8 Construct Oligos	5′-GATCCCCGAGATTTTAAGCGCGGTACCTCGAGGCATG-3′<br>3′-GGGCTCTAAAATTCGCGCCATGGAGCTCC-5′
Tag 9 Construct Oligos	5′-GATCCCTTGCCTATTAAGCGCGGTACCTCGAGGCATG-3′<br>3′-GGAACGGATAATTCGCGCCATGGAGCTCC-5′
Tag 10 Construct Oligos	5′-GATCCCACGATTGTTAAGCGCGGTACCTCGAGGCATG-3′<br>3′-GGTGCTAACAATTCGCGCCATGGAGCTCC-5′
Tag 11 Construct Oligos	5′-GATCCCGTCTTGATTAAGCGCGGTACCTCGAGGCATG-3′<br>3′-GGCAGAACTAATTCGCGCCATGGAGCTCC-5′
Clone sequencing primer	5′-CAGCAACGCGGCCTTTTTACG–3′
Forward Primer – STM PCR amplification step	5′-TACAGCGTGAGCTATGAGAAAGCG-3′
Reverse Primer – STM PCR amplification step	5′-CCTAAGGTACCGCATGCCT-3′
**Primers for tag-specific labeling step[a]**	
Tag1 (tLUA_64)	5′-CTACATATTCAAATTACTACTTACA/CCGCGCTTAAACGTTCA-3′
Tag2 (tLUA_24)	5′-TCAATTACCTTTTCAATACAATACA/CCGCGCTTAAATAGCCT-3′
Tag3 (tLUA_36)	5′-CAATTCATTTCATTCACAATCAATA/CCGCGCTTAAAAGTCTC-3′
Tag4 (tLUA_82)	5′-TACATACACTAATAACATACTCATA/CCGCGCTTAATAACGTG-3′
Tag5 (tLUA_12)	5′-TACACTTTCTTTCTTTCTTTCTTTA/CCGCGCTTAAACTGGTA-3′
Tag6 (tLUA_30)	5′-TTACCTTTATACCTTTCTTTTTACA/CCGCGCTTAAGCATGTT-3′
Tag7 (tLUA_68)	5′-TCATAATCTCAACAATCTTTCTTTA/CCGCGCTTAATGTAACC-3′
Tag8 (tLUA_55)	5′-TATATACACTTCTCAATAACTAACA/CCGCGCTTAAAATCTCG-3′
Tag9 (tLUA_52)	5′-TCAATCATCTTTATACTTCACAATA/CCGCGCTTAATAGGCAA-3′
Tag10 (tLUA_2)	5′-CTTTATCAATACATACTACAATCAA/CCGCGCTTAACAATCGT-3′
Tag11 (tLUA_80)	5′-CTAACTAACAATAATCTAACTAACA/CCGCGCTTAATCAAGAC-3′
Universal (tLUA_14)	5′-CTACTATACATCTTACTATACTTTC/GGTCTCCTAAGGTACCG-3′

a The Luminex FlexMAP™ microsphere specificity is indicated in parentheses. The forward slash represents the demarcation point between the Luminex FlexMap™ oligonucleotide-specific sequence and the STM tag-specific primer sequence.

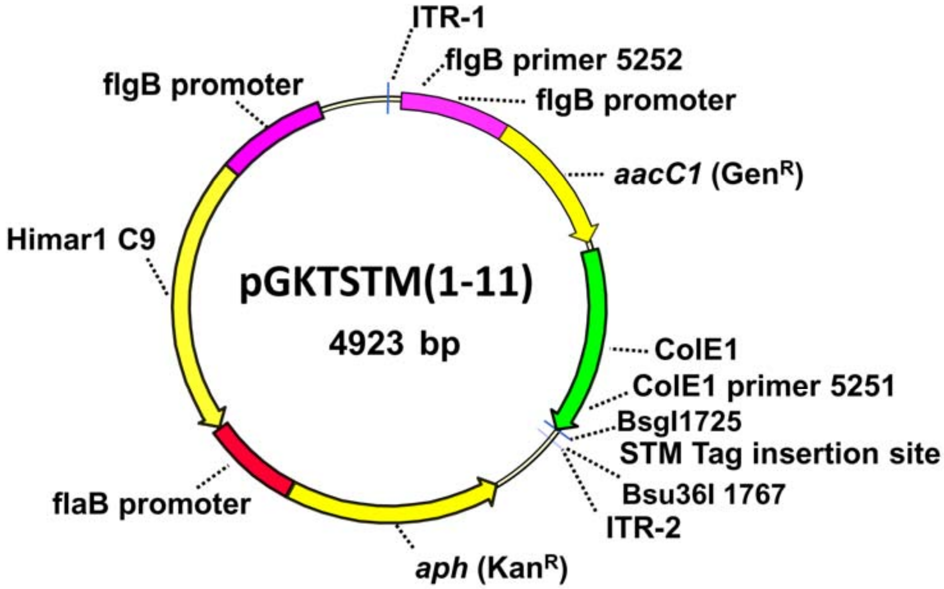

Fig. 1. Construction of signature-tagged suicide himar1 transposon vectors pGKTSTM1-pGKTSTM11 for transposon mutagenesis of infectious *Borrelia burgdorferi*. The signature-tagged suicide vectors pGKTSTM1-pGKTSTM11 vectors were constructed by inserting STM tags into a region between the ColE1 origin and Inverted Terminal Repeat 2 (ITR2) in Himar1 transposon vector pGKT (2).

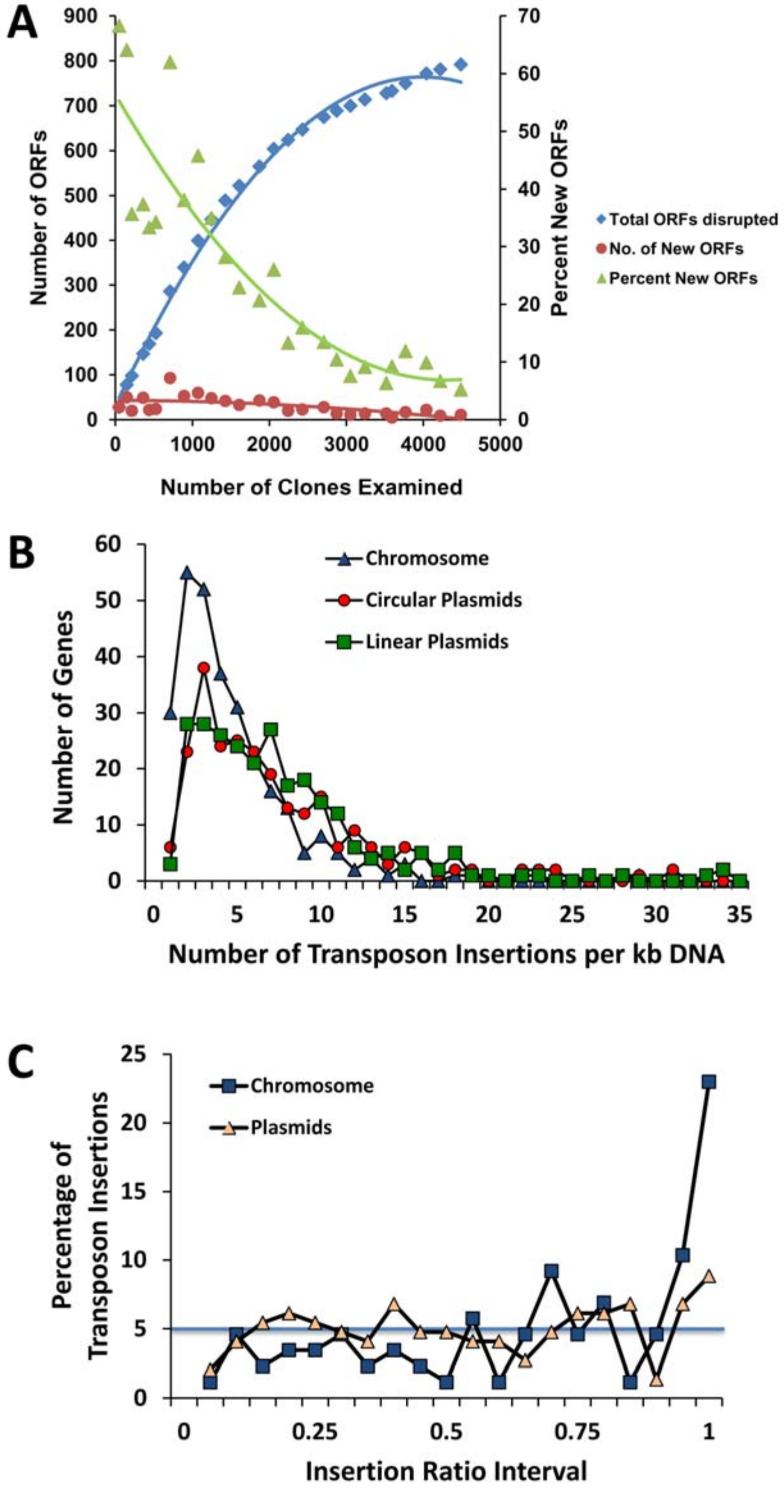

Fig. 2. Characteristics of transposon insertions in *B. burgdorferi* genes. (A) The number of genes with transposon insertions approaches saturation. Each data point represents a separate sequencing run for insertion site determination, which typically included 1-3 transformations. Total ORFs disrupted is the cumulative number of ORFs with insertions (final N = 790). Number of new ORFs is the number of ORFs in each sequencing run in which the ORF was not disrupted previously. Percent new ORFs represents the percentage of clones in a sequencing run that were in a newly disrupted ORF. Trendlines were generated in Microsoft Excel using the polynomial function. (B) Number of transposon insertions per kb DNA for genes in the chromosome, circular plasmids, and linear plasmids. Results for the 790 predicted genes with insertions are shown. (C) Preferential localization of transposon insertions in the last 10% of the open reading frame for the 233 genes that had only one insertion. Results are shown as the percentage of insertions in the insertion ratio intervals 0-0.05, 0.05-0.10, etc.

Fig. 3. Maps indicating the locations of STM transposon insertion sites in the plasmids of *B. burgdorferi* 5A18NP1. The genes are indicated as arrows; ORFs with dashed borders and an asterisk after the gene designation are pseudogenes. The key at the bottom of the figure indicates the color codes for four paralogous gene families (PFs) that have been postulated to be involved in plasmid replication or partitioning. The triangles on the line underlying the genes represent transposon insertion sites. Regions lacking transposon insertions (and thus containing genes potentially required for *B. burgdorferi* survival or plasmid retention) are marked as orange rectangles below the transposon insertion sites. The final panel is a histogram of the distribution of insertion sites at 500 bp intervals in the chromosome.

lp5 (Plasmid T)
(5,228 bp)

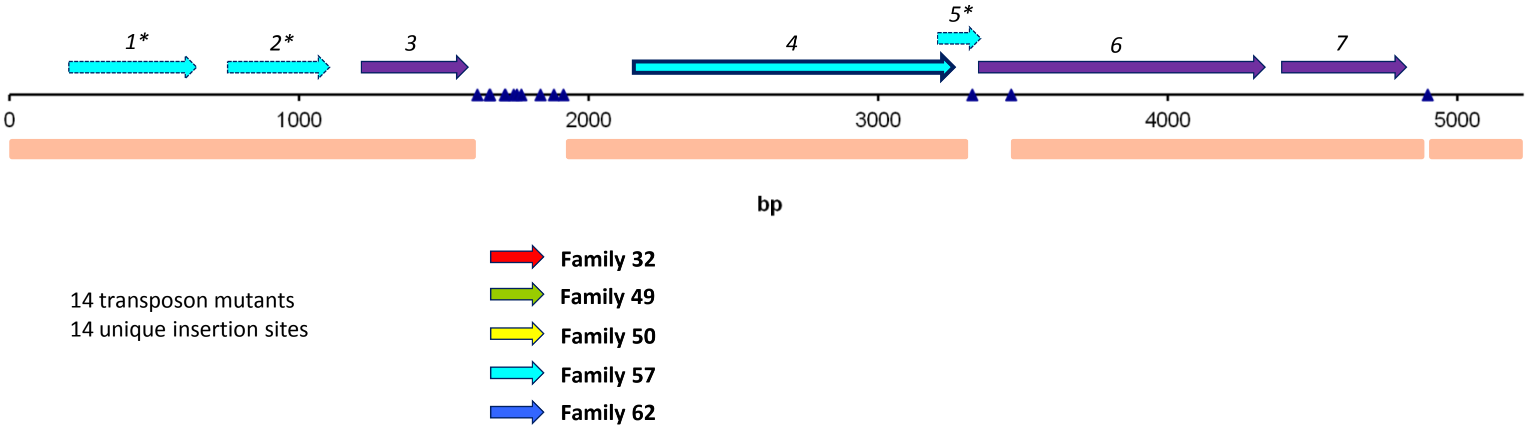

lp17 (Plasmid D)
(16,823 bp)

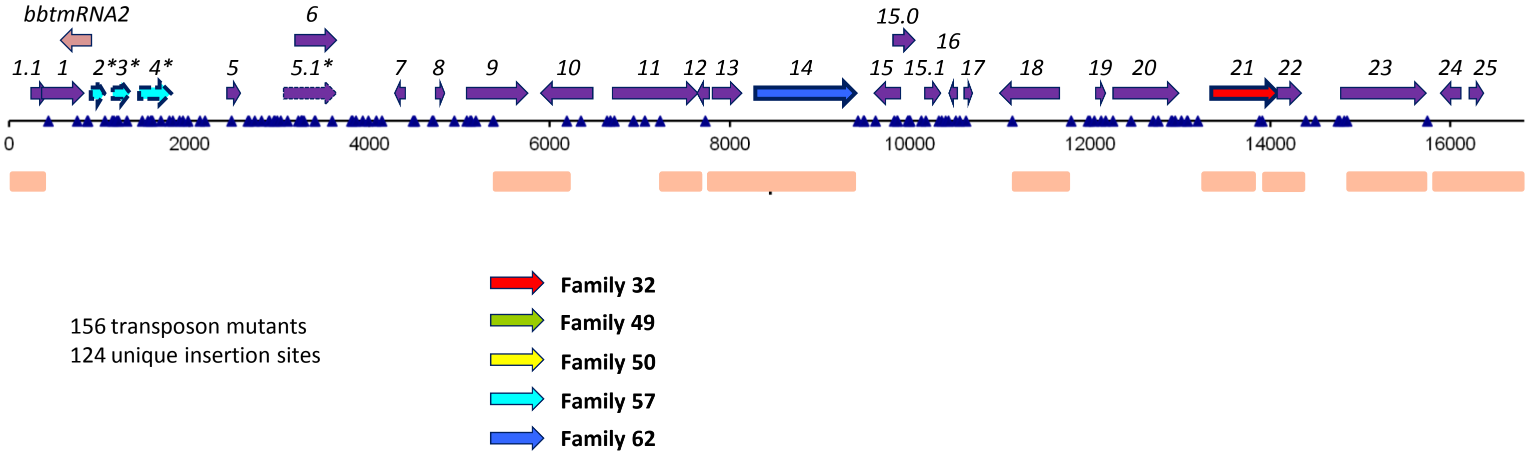

lp21 (Plasmid U)
(18,753 bp)

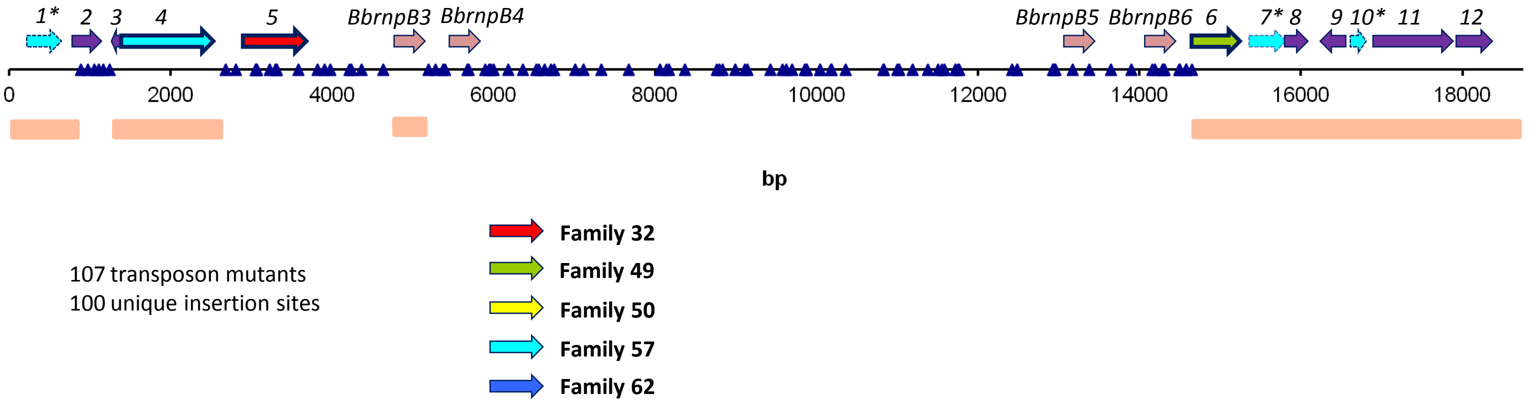

107 transposon mutants
100 unique insertion sites

lp25 (Plasmid E)
(24,177 bp)

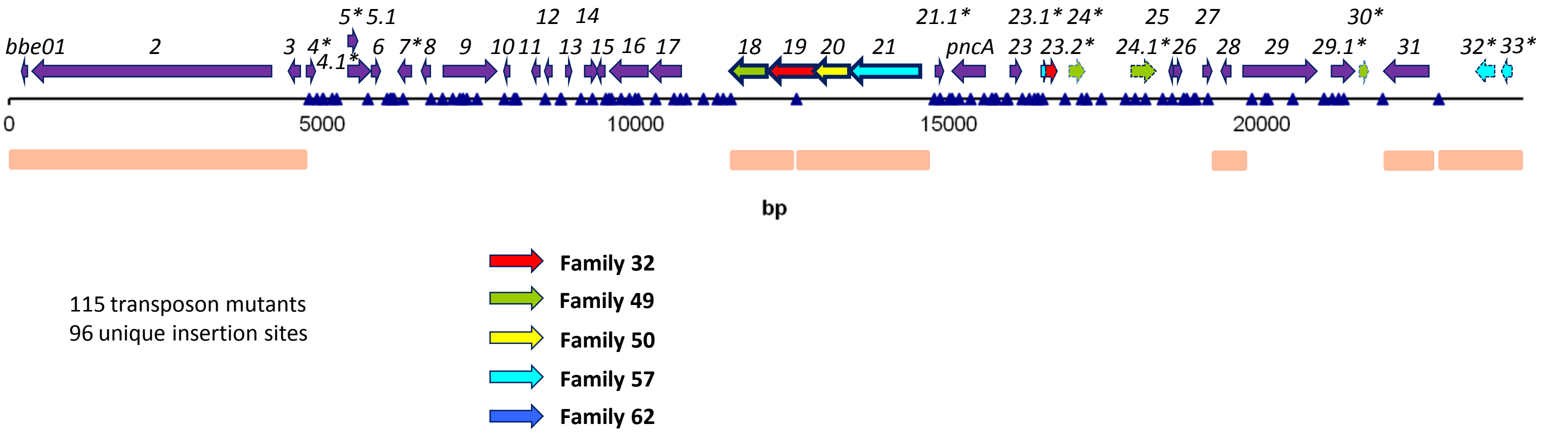

lp28-1 (Plasmid F)
(28,155 bp)

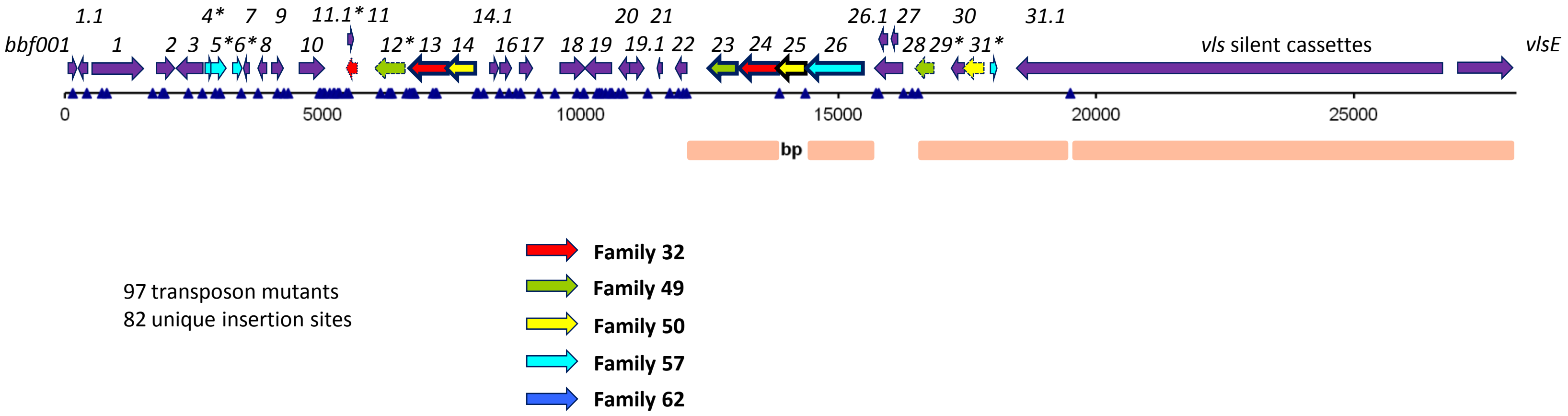

97 transposon mutants
82 unique insertion sites

lp28-2 (Plasmid G)
(29,766 bp)

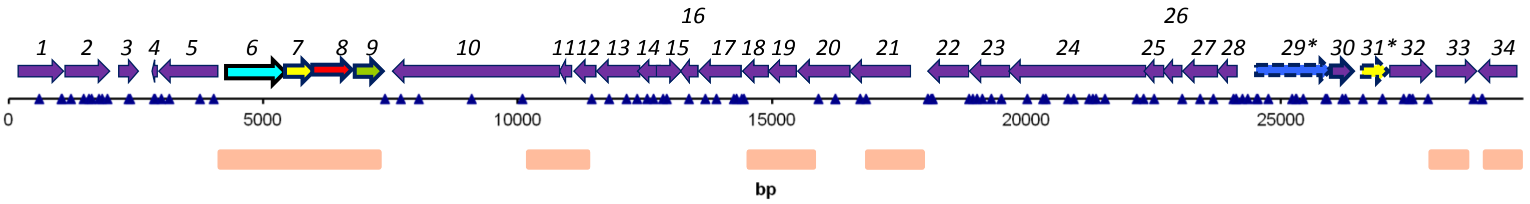

104 transposon mutants
96 unique insertion sites

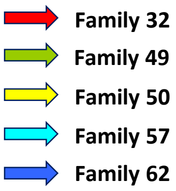

lp28-3 (Plasmid H)
(28,601 bp)

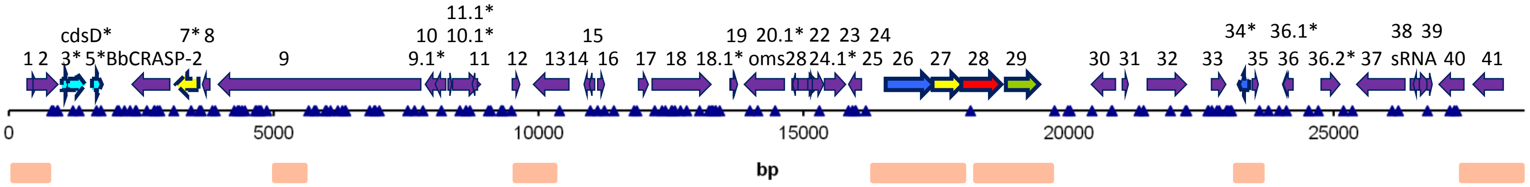

160 transposon mutants
134 unique insertion sites

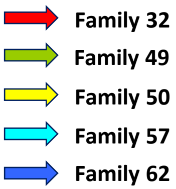

lp36 (Plasmid K)
(29,766 bp)

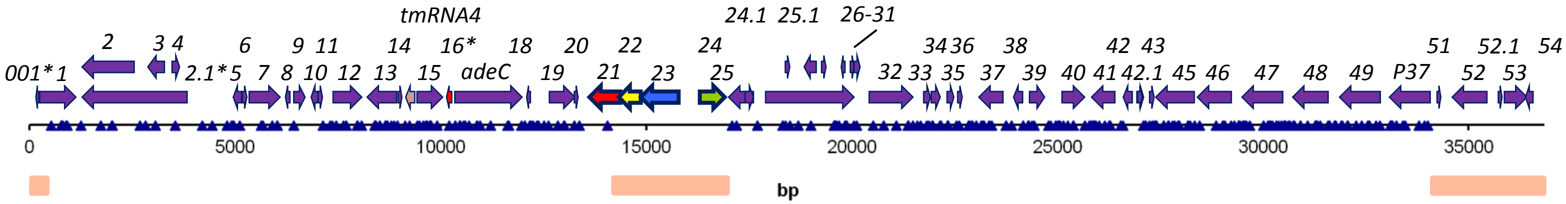

285 transposon mutants
266 unique insertion sites

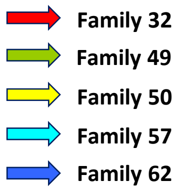

lp38 (Plasmid J)
(38,829 bp)

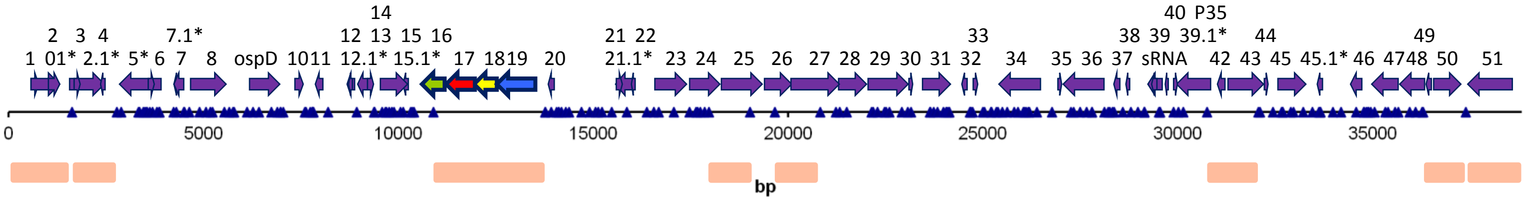

214 transposon mutants
185 unique insertion sites

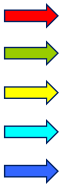

lp54 (Plasmid A)
(53,561 bp)

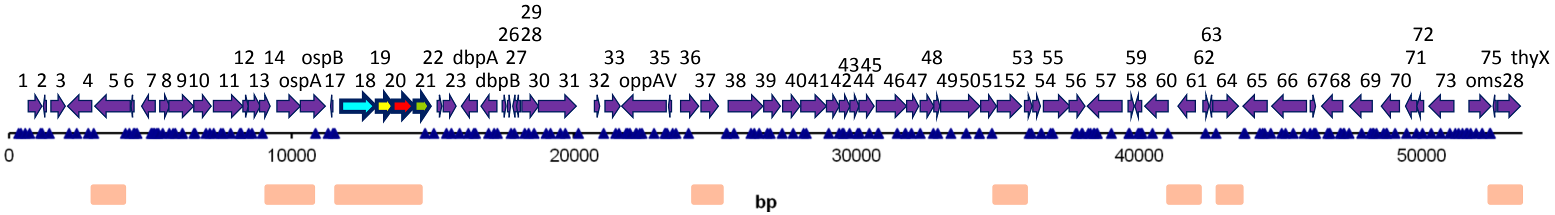

223 transposon mutants
195 unique insertion sites

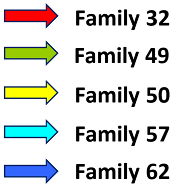

cp9 (Plasmid C)
(9,386 bp)

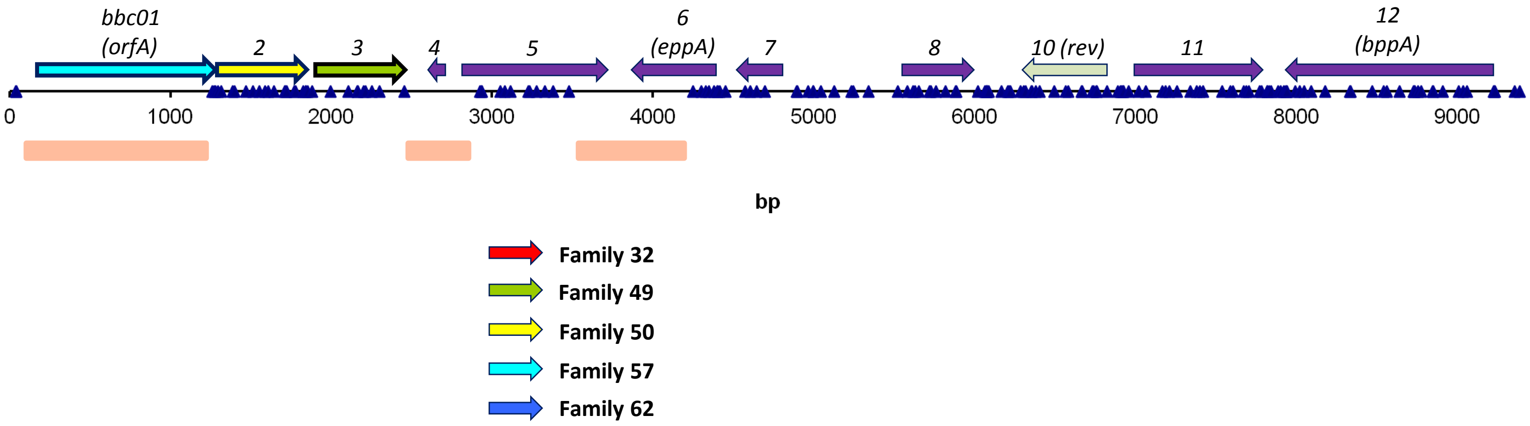

225 transposon mutants
169 unique sites

cp26 (Plasmid B)
(26,498 bp)

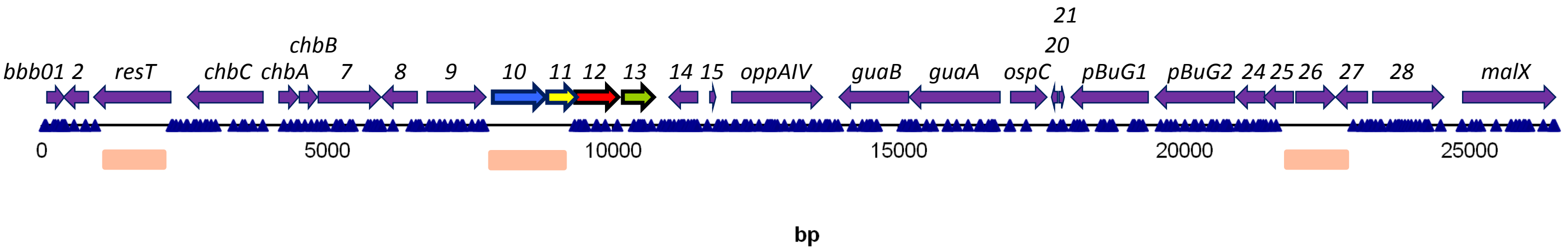

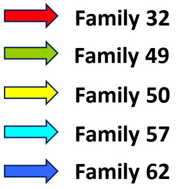

372 transposon mutants
302 unique insertion sites

cp32-1 (Plasmid P)
(30,750 bp)

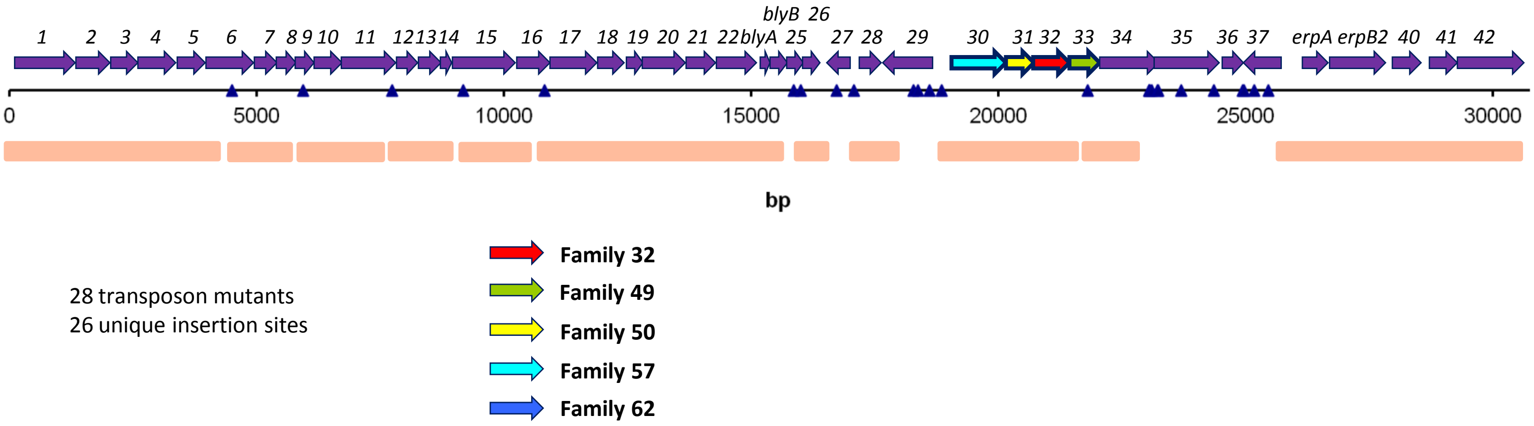

28 transposon mutants
26 unique insertion sites

cp32-3 (Plasmid S)
(30,233 bp)

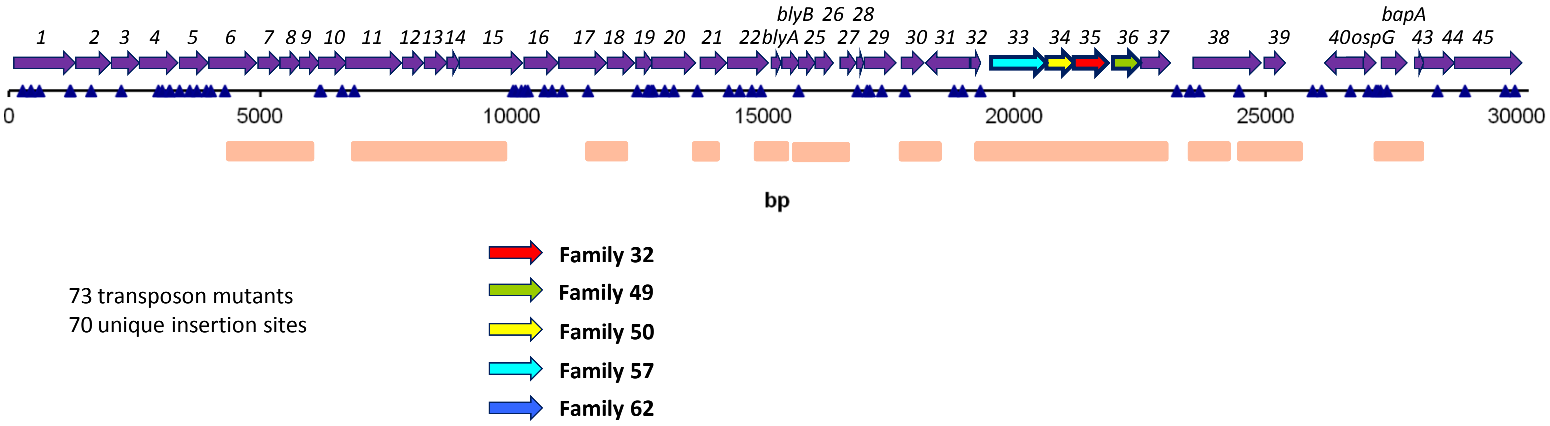

73 transposon mutants
70 unique insertion sites

cp32-4 (Plasmid R)
(30,299 bp)

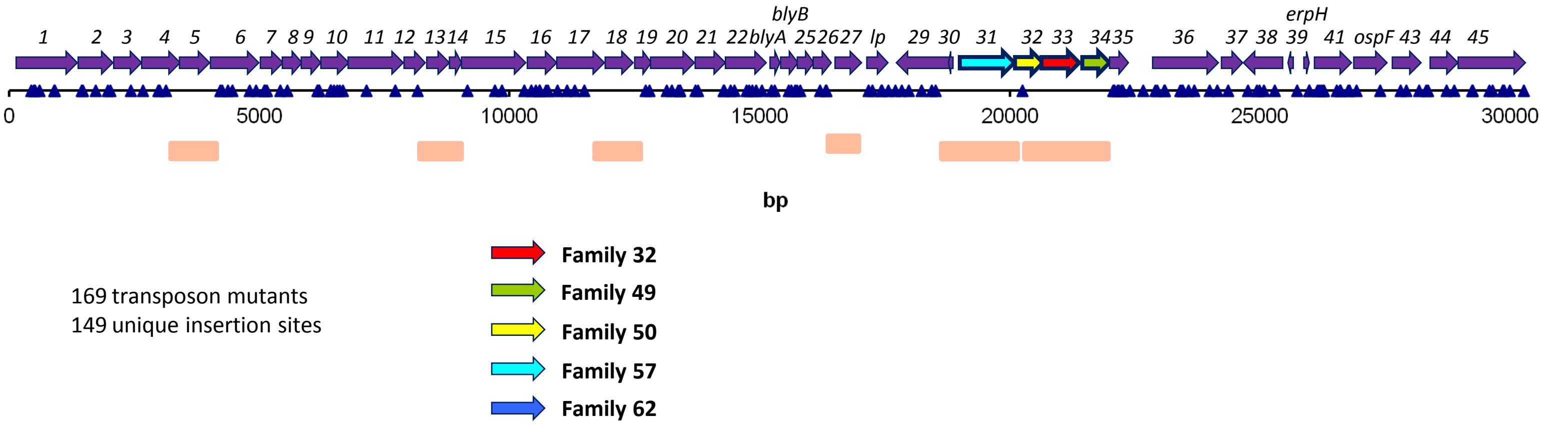

169 transposon mutants
149 unique insertion sites

cp32-6 (Plasmid M)
(29,838 bp)

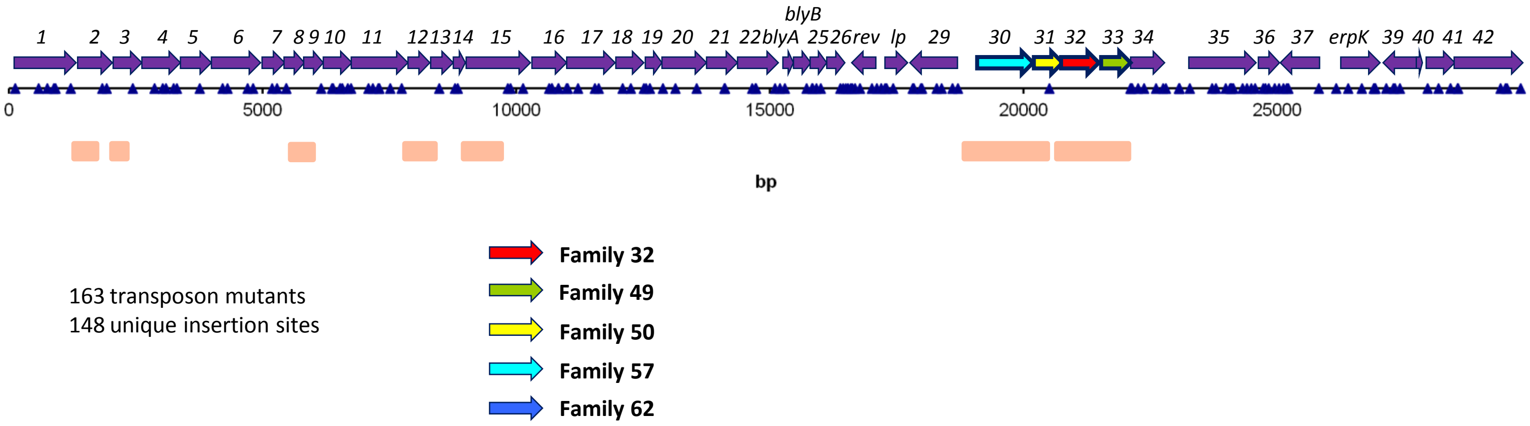

163 transposon mutants
148 unique insertion sites

cp32-7 (Plasmid O)
(30,800 bp)

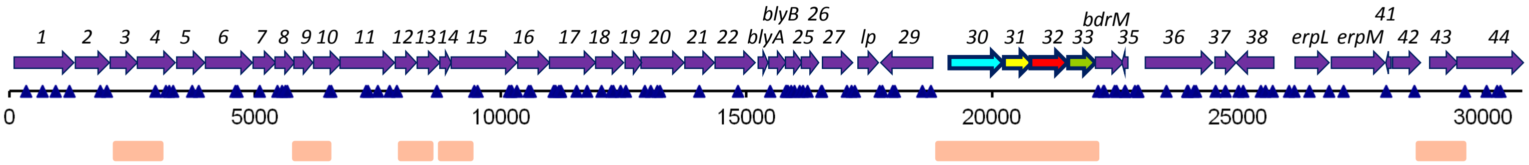

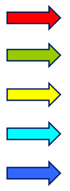

116 transposon mutants
110 unique insertion sites

cp32-8 (Plasmid L)
(30,885 bp)

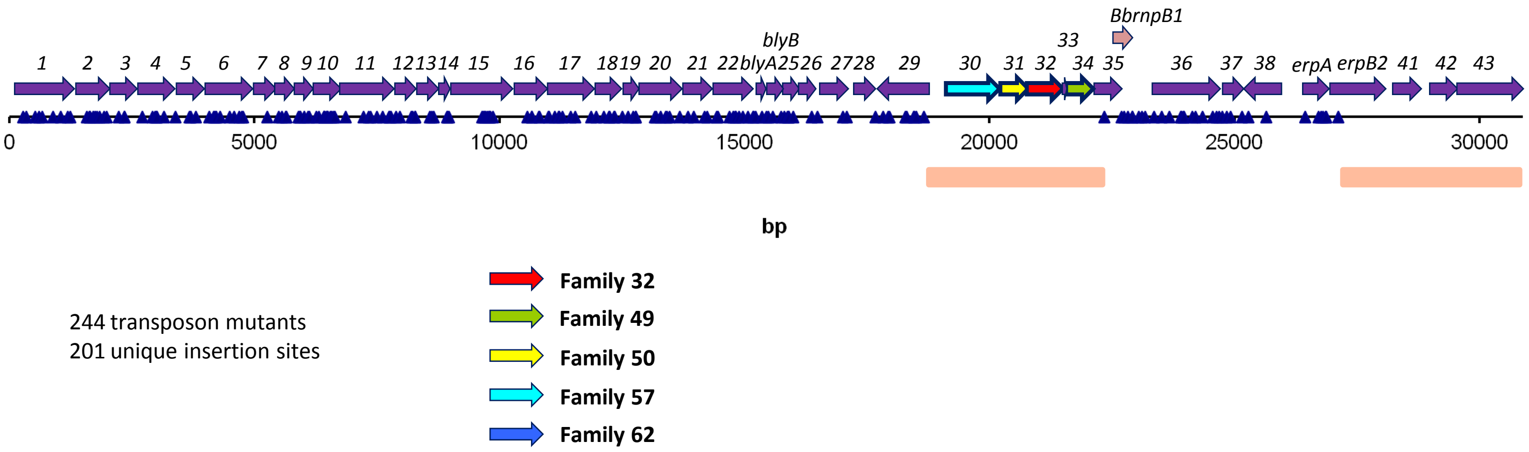

244 transposon mutants
201 unique insertion sites

cp32-9 (Plasmid N)
(30,651 bp)

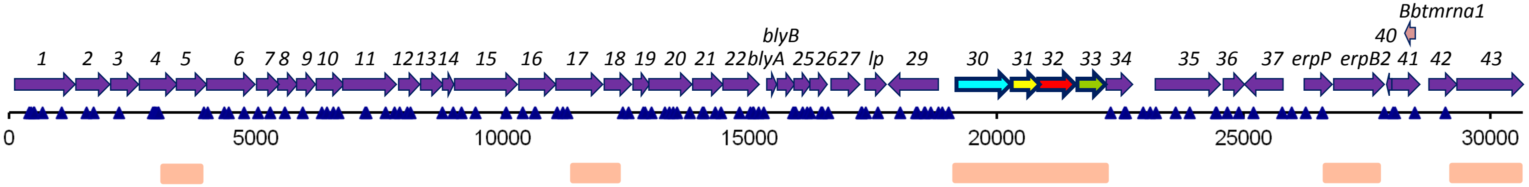

bp

128 transposon mutants
114 unique insertion sites

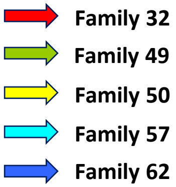

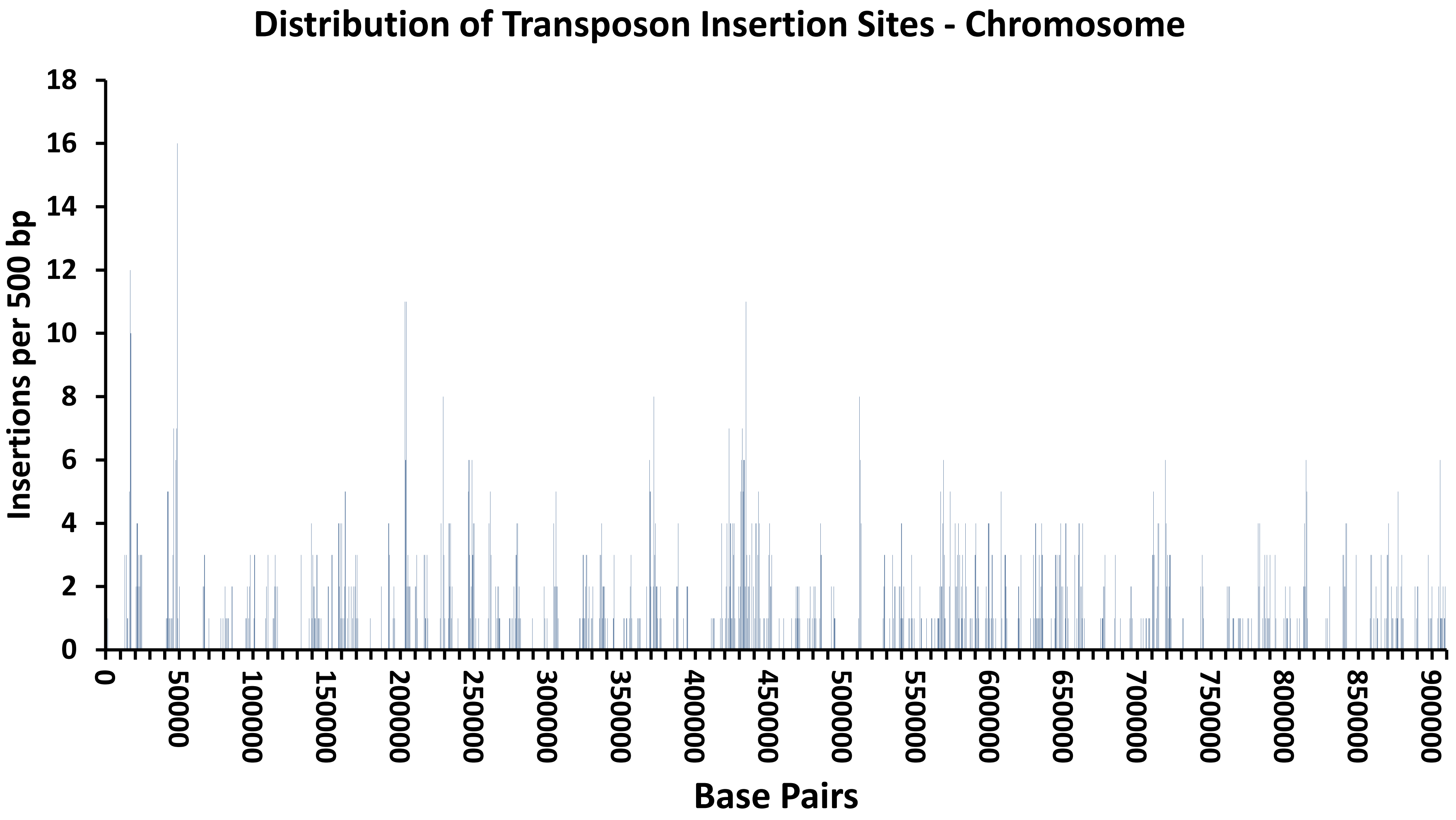
Distribution of Transposon Insertion Sites - Chromosome
Insertions per 500 bp
0
2
4
6
8
10
12
14
16
18
0
50000
100000
150000
200000
250000
300000
350000
400000
450000
500000
550000
600000
650000
700000
750000
800000
850000
900000
Base Pairs

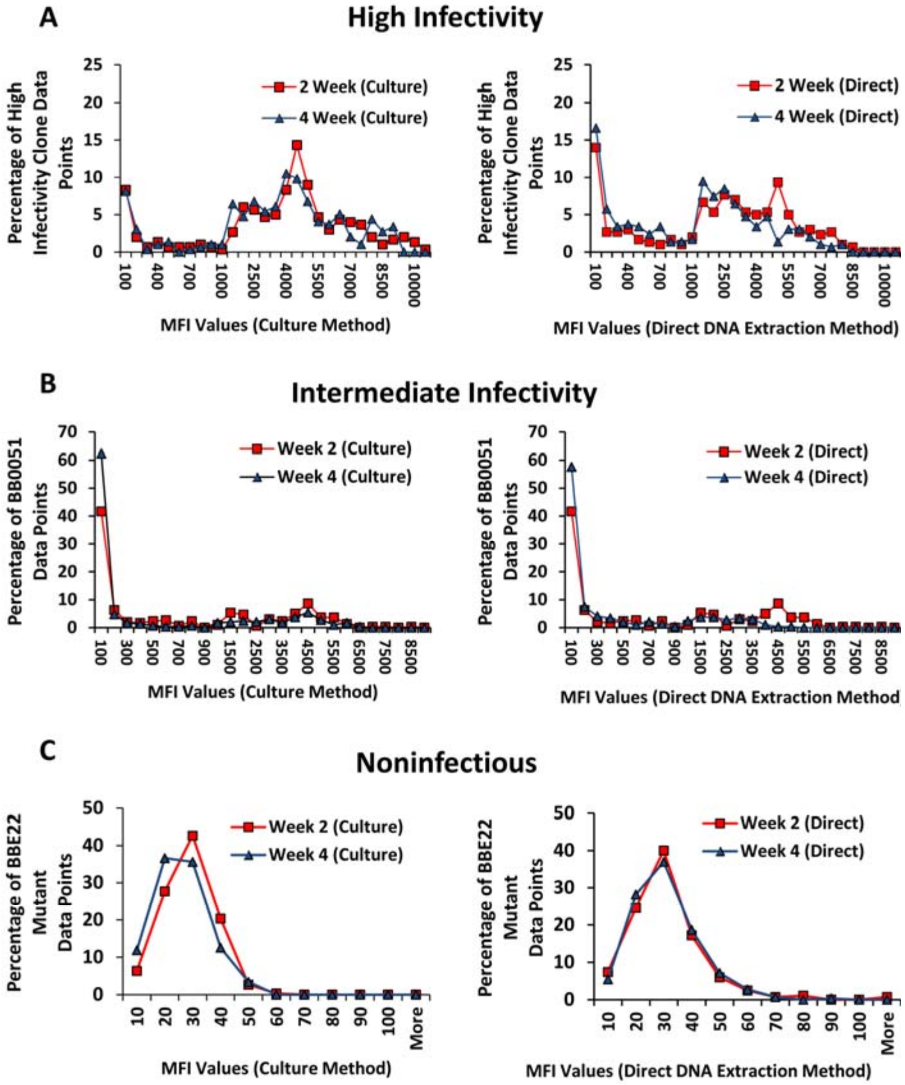

Fig. 4. Distribution of individual MFI values obtained with high, intermediate, and low infectivity STM transposon mutant clones. Results are shown for (a) the 20 clones that had the highest mean MFI values, (b) the intermediate infectivity BB0051 mutant T02P01A01, and (c) the low infectivity bbe02 (pncA) mutant T01P01A11. T01P01A11 and T02P01A01 are the negative and positive controls used in STM experiments. The data are subdivided into culture and direct DNA extraction methods, and into 2 week and 4 week samples. The minimum number of individual data points for each histogram is 286.

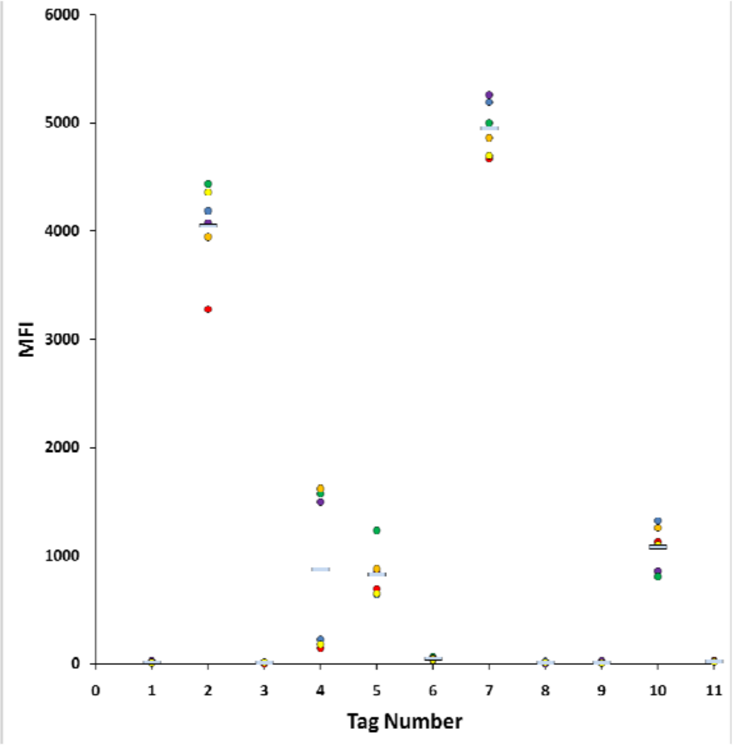

Fig. 5. MFI values obtained by Luminex-based STM analysis are reproducible. The results of six replicate determinations using Mouse 1 2 week direct tissue DNA extraction sample from Set 51 are shown. The X axis represents the 11 clones used to inoculate the animals. Consistent results were obtained in the 6 replicates.

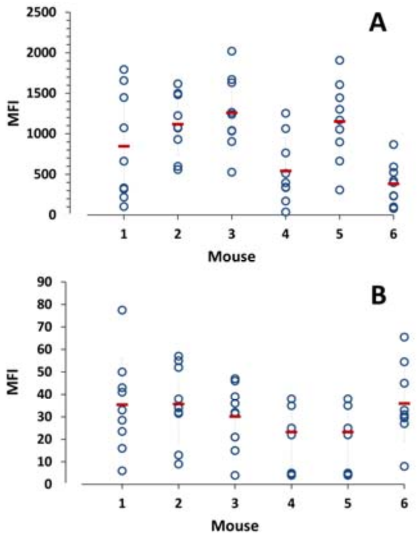

Fig. 6. Sampling error may contribute to the variability of Luminex-based MFI values obtained in mouse infectivity analyses. The STM Set 51 skin inoculation site tissue from 6 mice were used; mice 1-3 were infected for 2 weeks, whereas mice 4-6 were infected for 4 weeks prior to analysis. For each mouse, the skin inoculation site was subdivided into 9 equally-sized pieces, and each piece was processed separately by the direct DNA extraction method and evaluated for clone content by Luminex STM analysis. The results presented are for (a) the relatively high infectivity clone T07TC483 and (b) the low infectivity pncA mutant T01P01A01 (note difference in Y-axis scale). The MFI values obtained for each tissue specimen are shown; the red line indicates the mean value.

Figure 7. Heat map representations of the mouse infectivity of transposon mutants in the genes of *B. burgdorferi* plasmids. The plasmids shown are (A) cp26, (B) lp25, (C) lp28-1, (D) lp36, and (E) lp54. For each clone, results from Luminex STM analysis (see Fig. 4, Table S3) were grouped by week post inoculation (2 weeks or 4 weeks) and by DNA preparation method (Culture = use of organisms cultured from tissue; Tissue = use of DNA extracted directly from tissue). The data in each group were scored according to the percentage of samples with MFI values above the negative threshold (100) or to the mean MFI value; each result was then color coded as indicated in the key. Each colored box in the figure corresponds to 12 to 15 data points (typically 5 tissues from 3 mice). The composite results obtained in all experiments for the positive (*bb0051*) and negative (*pncA*) control mutants are provided at the right side of each figure. HP, hypothetical protein; CHP, conserved hypothetical protein; LP, lipoprotein; OMP, outer membrane protein; OSP, outer surface protein. Keys that indicate the clones and STM experiments utilized in each figure panel are provided at http://www.uth.tmc.edu/pathology/borrelia/.

A

cp26

		Gene (Insertion ratio) Gene Name																																								
Sample Type	Week PI	BBB01 (0.11) *acyp*	BBB02 (0.44) CHP	BBB03 (0.94) *resT*	BBB04 (0.20) *chbC*	BBB04 (0.23) *chbC*	BBB05 (0.42) *chbA*	BBB06 (0.13) *chbB*	BBB06 (0.86) *chbB*	BBB07 (0.11) *osp*	BBB08 (0.10) LP	BBB08 (0.10) LP	BBB09 (0.09) LP	BBB09 (0.28) LP	BBB12 (0.27) PF32	BBB13 (0.46) PF49	BBB14 (0.11) CHP	BBB14 (0.11) CHP	BBB14 (0.43) CHP	BBB15 (0.06) CHP	BBB16 (0.06) *oppA-4*	BBB16 (0.17) *oppA-4*	BBB16 (0.17) *oppA-4*	BBB17 (0.41) *guaB*	BBB18 (0.18) *guaA*	BBB19 (0.07) *ospC*	BBB19 (0.51) *ospC*	BBB20 (0.41) CHP	BBB21 (0.76) CHP	BBB22 (0.15) *pbuG1*	BBB22 (0.44) *pbuG1*	BBB23 (0.19) *pbuG2*	BBB24 (0.15) CHP	BBB25 (0.50) CHP	BBB25 (0.67) CHP	BBB27 (0.10) LP	BBB27 (0.10) LP	BBB28 (0.27) CHP	BBB29 (0.17) *malX*	BBB29 (0.17) *malX*	BB0051 CHP (0.79) (+)	BBE22 (0.44) *pncA* (-)
		Percent Sites Positive																																								
T	2																																									
T	4								ND												ND													ND								
C	2				ND																								ND													
C	4				ND				ND												ND								ND					ND								
Overall																																										
		Mean MFI Value																																								
T	2																																									
T	4								ND												ND													ND								
C	2				ND																								ND													
C	4				ND				ND												ND								ND					ND								
Overall																																										

Percent Positive	81 - 100	61 - 80	41 - 60	21 - 40	0 - 20

Mean MFI	>1000	501-1000	100-500	<100

B

lp25

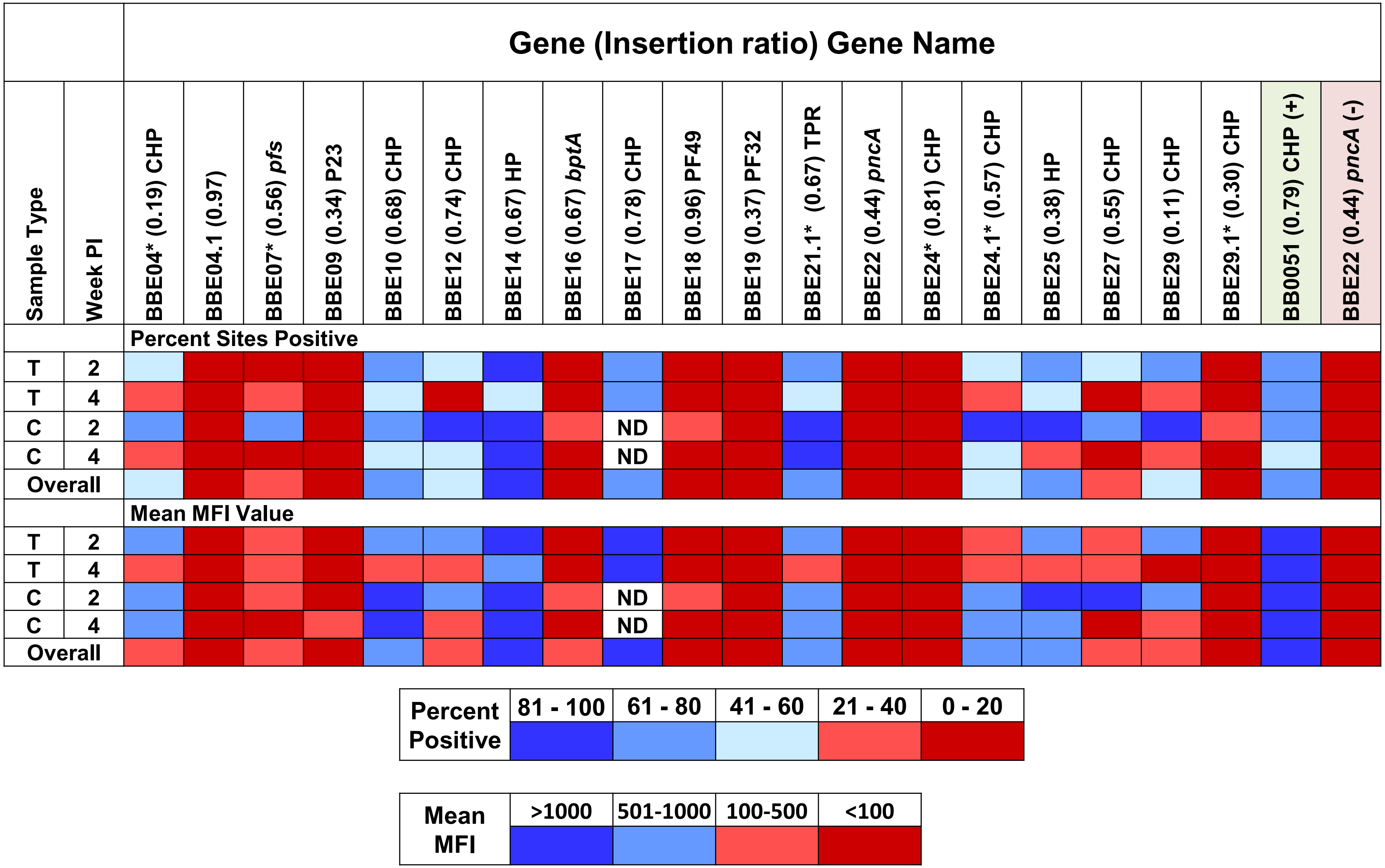

Gene (Insertion ratio) Gene Name
Sample Type
Week PI
BBE04* (0.19) CHP
BBE04.1 (0.97)
BBE07* (0.56) pfs
BBE09 (0.34) P23
BBE10 (0.68) CHP
BBE12 (0.74) CHP
BBE14 (0.67) HP
BBE16 (0.67) bptA
BBE17 (0.78) CHP
BBE18 (0.96) PF49
BBE19 (0.37) PF32
BBE21.1* (0.67) TPR
BBE22 (0.44) pncA
BBE24* (0.81) CHP
BBE24.1* (0.57) CHP
BBE25 (0.38) HP
BBE27 (0.55) CHP
BBE29 (0.11) CHP
BBE29.1* (0.30) CHP
BB0051 (0.79) CHP (+)
BBE22 (0.44) pncA (-)
Percent Sites Positive
T 2
T 4
C 2
C 4
Overall
ND
ND
Mean MFI Value
T 2
T 4
C 2
C 4
Overall
ND
ND
Percent Positive
81 - 100
61 - 80
41 - 60
21 - 40
0 - 20
Mean MFI
>1000
501-1000
100-500
<100

C

lp28-1

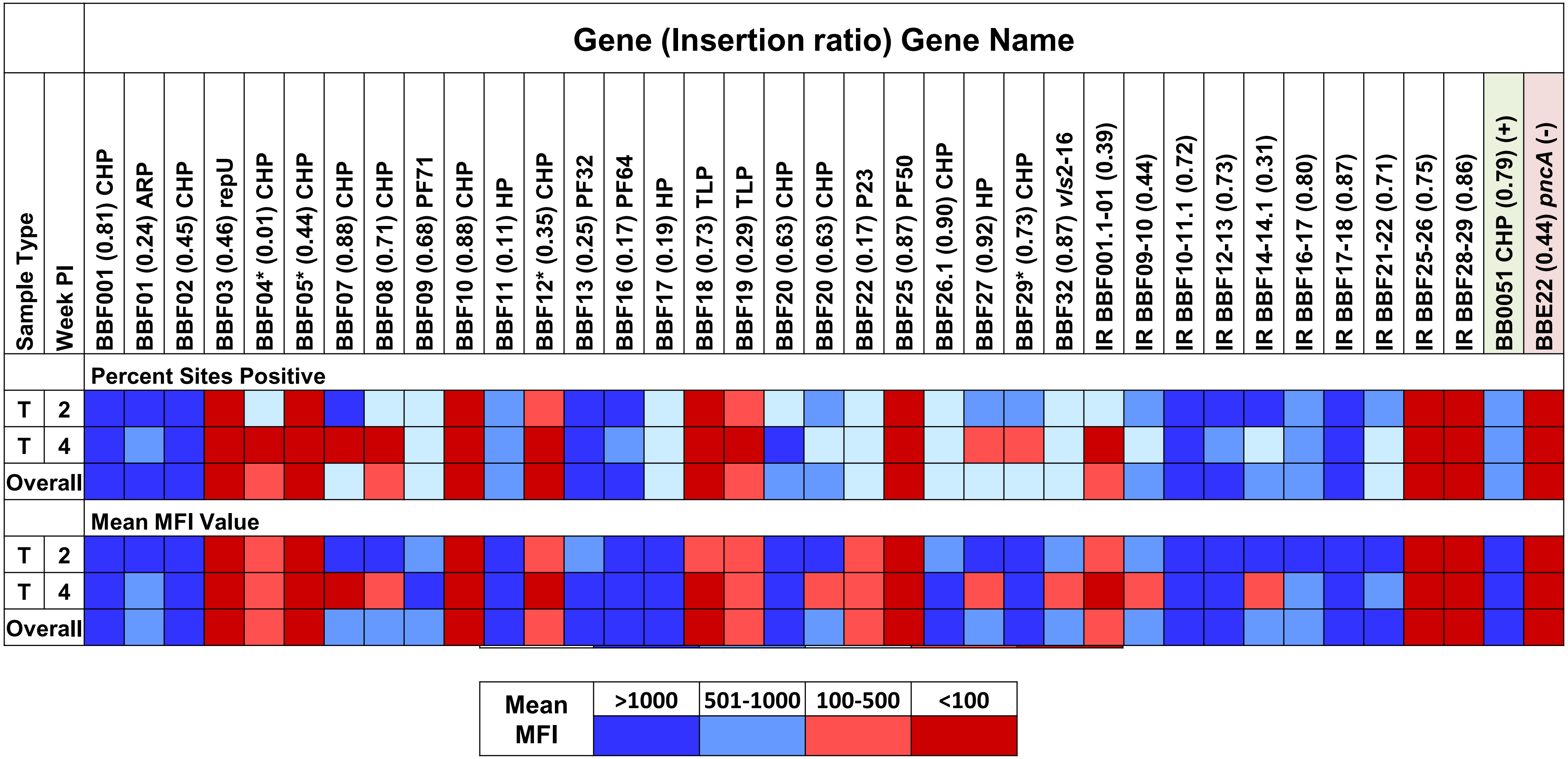
Gene (Insertion ratio) Gene Name
Sample Type
Week PI
BBF001 (0.81) CHP
BBF01 (0.24) ARP
BBF02 (0.45) CHP
BBF03 (0.46) repU
BBF04* (0.01) CHP
BBF05* (0.44) CHP
BBF07 (0.88) CHP
BBF08 (0.71) CHP
BBF09 (0.68) PF71
BBF10 (0.88) CHP
BBF11 (0.11) HP
BBF12* (0.35) CHP
BBF13 (0.25) PF32
BBF16 (0.17) PF64
BBF17 (0.19) HP
BBF18 (0.73) TLP
BBF19 (0.29) TLP
BBF20 (0.63) CHP
BBF20 (0.63) CHP
BBF22 (0.17) P23
BBF25 (0.87) PF50
BBF26.1 (0.90) CHP
BBF27 (0.92) HP
BBF29* (0.73) CHP
BBF32 (0.87) *vls*2-16
IR BBF001.1-01 (0.39)
IR BBF09-10 (0.44)
IR BBF10-11.1 (0.72)
IR BBF12-13 (0.73)
IR BBF14-14.1 (0.31)
IR BBF16-17 (0.80)
IR BBF17-18 (0.87)
IR BBF21-22 (0.71)
IR BBF25-26 (0.75)
IR BBF28-29 (0.86)
BB0051 CHP (0.79) (+)
BBE22 (0.44) *pncA* (-)
Percent Sites Positive
T 2
T 4
Overall
Mean MFI Value
T 2
T 4
Overall
Mean MFI
>1000
501-1000
100-500
<100

D lp36

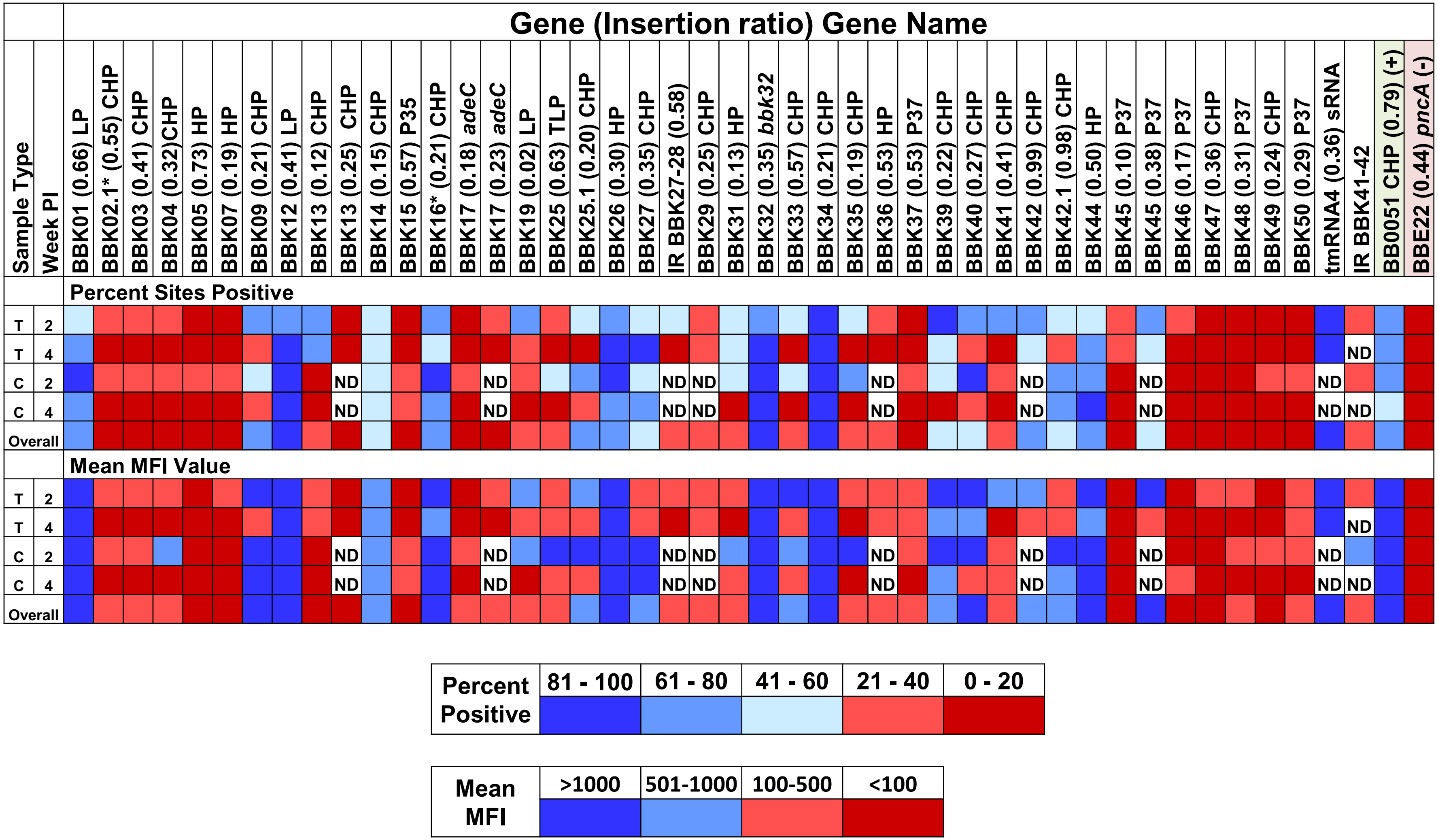
Gene (Insertion ratio) Gene Name
Sample Type
Week PI
BBK01 (0.66) LP
BBK02.1* (0.55) CHP
BBK03 (0.41) CHP
BBK04 (0.32)CHP
BBK05 (0.73) HP
BBK07 (0.19) HP
BBK09 (0.21) CHP
BBK12 (0.41) LP
BBK13 (0.12) CHP
BBK13 (0.25) CHP
BBK14 (0.15) CHP
BBK15 (0.57) P35
BBK16* (0.21) CHP
BBK17 (0.18) adeC
BBK17 (0.23) adeC
BBK19 (0.02) LP
BBK25 (0.63) TLP
BBK25.1 (0.20) CHP
BBK26 (0.30) HP
BBK27 (0.35) CHP
IR BBK27-28 (0.58)
BBK29 (0.25) CHP
BBK31 (0.13) HP
BBK32 (0.35) bbk32
BBK33 (0.57) CHP
BBK34 (0.21) CHP
BBK35 (0.19) CHP
BBK36 (0.53) HP
BBK37 (0.53) P37
BBK39 (0.22) CHP
BBK40 (0.27) CHP
BBK41 (0.41) CHP
BBK42 (0.99) CHP
BBK42.1 (0.98) CHP
BBK44 (0.50) HP
BBK45 (0.10) P37
BBK45 (0.38) P37
BBK46 (0.17) P37
BBK47 (0.36) CHP
BBK48 (0.31) P37
BBK49 (0.24) CHP
BBK50 (0.29) P37
tmRNA4 (0.36) sRNA
IR BBK41-42
BB0051 CHP (0.79) (+)
BBE22 (0.44) pncA (-)
Percent Sites Positive
T 2
T 4
C 2
C 4
Overall
Mean MFI Value
T 2
T 4
C 2
C 4
Overall
Percent Positive
81 - 100
61 - 80
41 - 60
21 - 40
0 - 20
Mean MFI
>1000
501-1000
100-500
<100

E

lp54

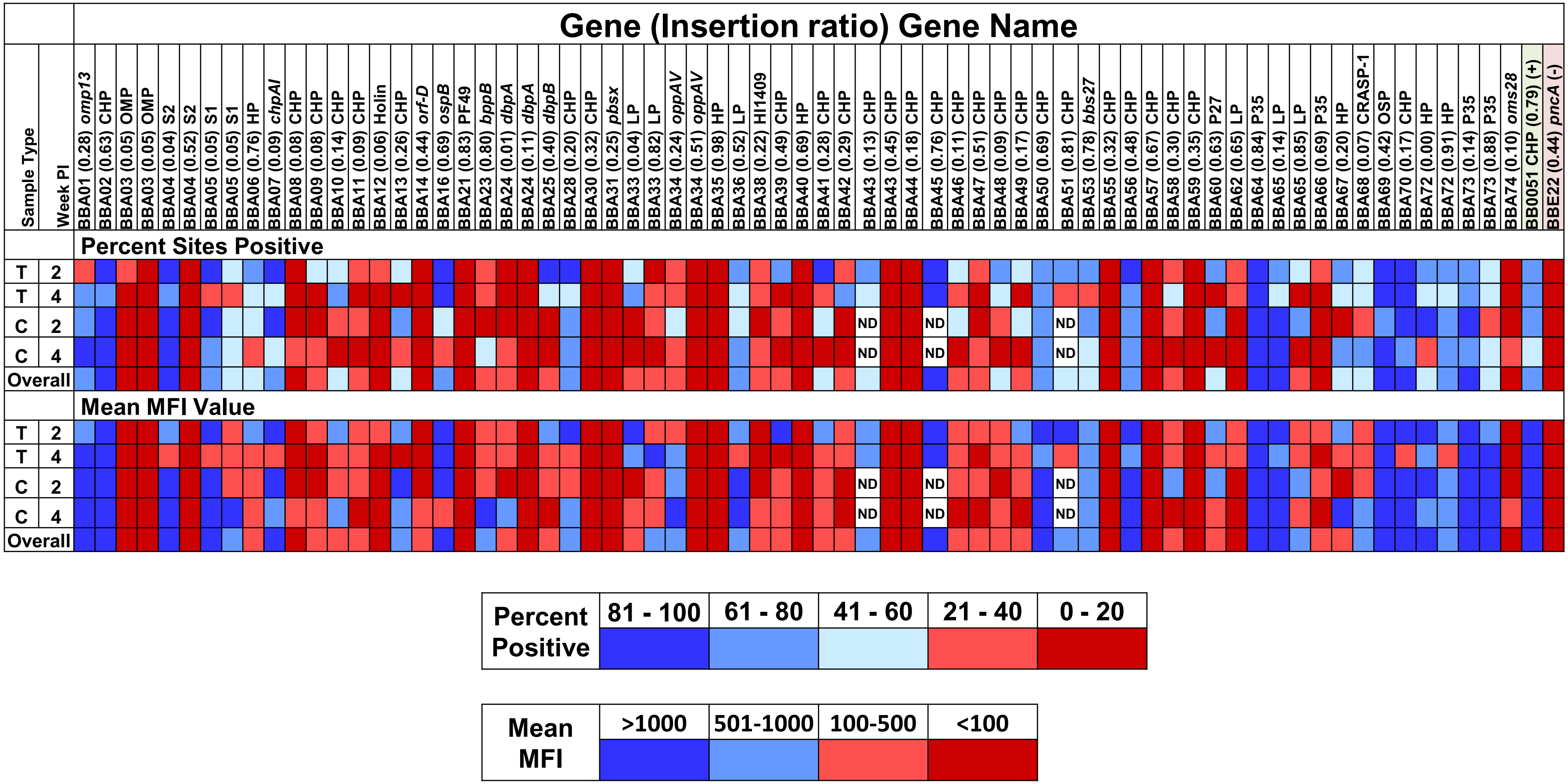
Gene (Insertion ratio) Gene Name
Sample Type
Week PI
BBA01 (0.28) omp13
BBA02 (0.63) CHP
BBA03 (0.05) OMP
BBA03 (0.05) OMP
BBA04 (0.04) S2
BBA04 (0.52) S2
BBA05 (0.05) S1
BBA05 (0.05) S1
BBA06 (0.76) HP
BBA07 (0.09) chpAI
BBA08 (0.08) CHP
BBA09 (0.08) CHP
BBA10 (0.14) CHP
BBA11 (0.09) CHP
BBA12 (0.06) Holin
BBA13 (0.26) CHP
BBA14 (0.44) orf-D
BBA16 (0.69) ospB
BBA21 (0.83) PF49
BBA23 (0.80) bppB
BBA24 (0.01) dbpA
BBA24 (0.11) dbpA
BBA25 (0.40) dbpB
BBA28 (0.20) CHP
BBA30 (0.32) CHP
BBA31 (0.25) pbsx
BBA33 (0.04) LP
BBA33 (0.82) LP
BBA34 (0.24) oppAV
BBA34 (0.51) oppAV
BBA35 (0.98) HP
BBA36 (0.52) LP
BBA38 (0.22) HI1409
BBA39 (0.49) CHP
BBA40 (0.69) HP
BBA41 (0.28) CHP
BBA42 (0.29) CHP
BBA43 (0.13) CHP
BBA43 (0.45) CHP
BBA44 (0.18) CHP
BBA45 (0.76) CHP
BBA46 (0.11) CHP
BBA47 (0.51) CHP
BBA48 (0.09) CHP
BBA49 (0.17) CHP
BBA50 (0.69) CHP
BBA51 (0.81) CHP
BBA53 (0.78) bbs27
BBA55 (0.32) CHP
BBA56 (0.48) CHP
BBA57 (0.67) CHP
BBA58 (0.30) CHP
BBA59 (0.35) CHP
BBA60 (0.63) P27
BBA62 (0.65) LP
BBA64 (0.84) P35
BBA65 (0.14) LP
BBA65 (0.85) LP
BBA66 (0.69) P35
BBA67 (0.20) HP
BBA68 (0.07) CRASP-1
BBA69 (0.42) OSP
BBA70 (0.17) CHP
BBA72 (0.00) HP
BBA72 (0.91) HP
BBA73 (0.14) P35
BBA73 (0.88) P35
BBA74 (0.10) oms28
BB0051 CHP (0.79) (+)
BBE22 (0.44) pncA (-)
Percent Sites Positive
T 2
T 4
C 2
C 4
Overall
Mean MFI Value
T 2
T 4
C 2
C 4
Overall
ND
Percent Positive
81 - 100
61 - 80
41 - 60
21 - 40
0 - 20
Mean MFI
>1000
501-1000
100-500
<100

Figure 8. Heat map representations of the mouse infectivity of transposon mutants in genes by functional group. Gene groups shown include those involved in (A) chemotaxis, (B) flagellar structure and assembly, (C) phosphoenol pyruvate phosphotransferase system (PEP-PTS), (D) other transport systems, and (E) plasmid maintenance. For each clone, results from Luminex STM analysis (see Fig. 4, Table S3) were grouped by week post inoculation (2 weeks or 4 weeks) and by DNA preparation method (Culture = use of organisms cultured from tissue; Tissue = use of DNA extracted directly from tissue). The data in each group were scored according to the percentage of samples with MFI values above the negative threshold (100) or to the mean MFI value; each result was then color coded as indicated in the key. Each colored box in the figure corresponds to 12 to 15 data points (typically 5 tissues from 3 mice). The composite results obtained in all experiments for the positive (*bb0051*) and negative (*pncA*) control mutants are provided at the right side of each figure. HP, hypothetical protein; CHP, conserved hypothetical protein; LP, lipoprotein; OMP, outer membrane protein; OSP, outer surface protein. Keys that indicate the clones and STM experiments utilized in each figure panel are provided at http://www.uth.tmc.edu/pathology/borrelia/.

A

Chemotaxis

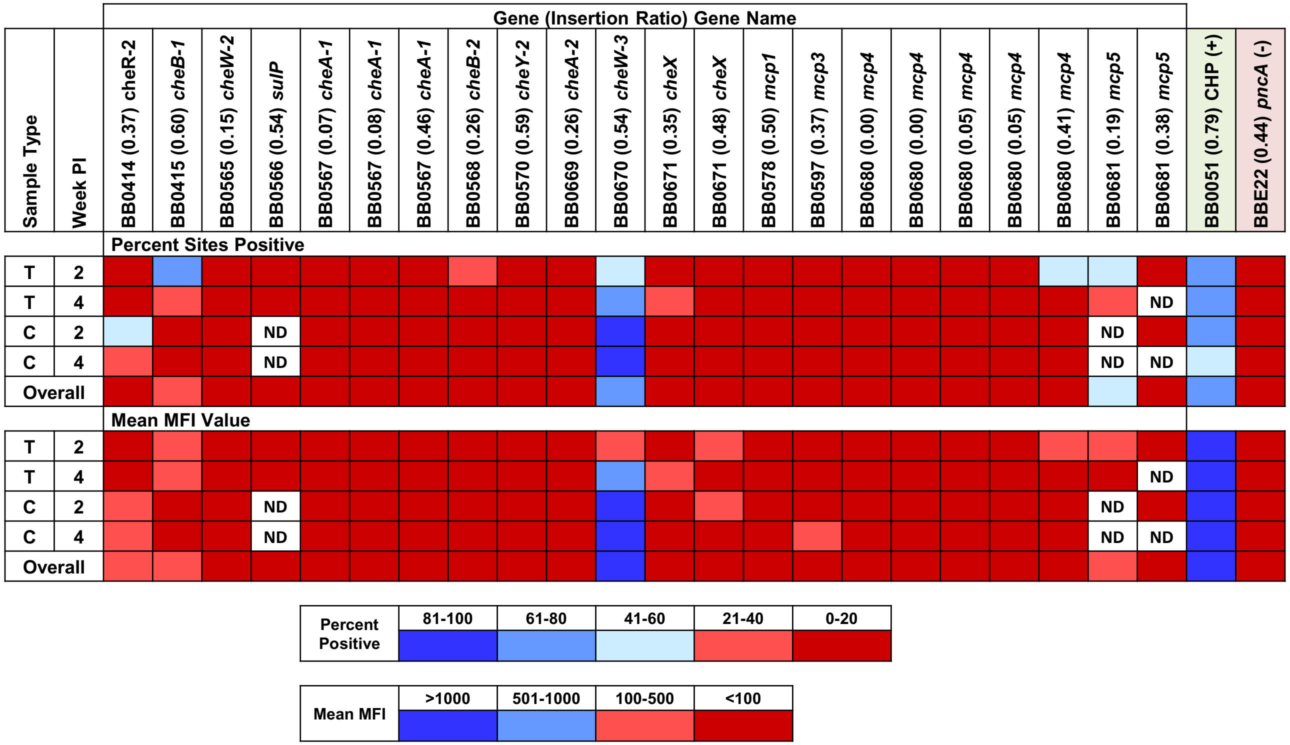

B

Flagellar Structure and Assembly

Sample Type	Week PI	BB0149 (0.28) *fliD*	BB0221 (0.33) *fliG-1*	BB0276 (0.75) *fliZ*	BB0287 (0.65) *flbA*	BB0288 (0.79) *fliI*	BB0289 (0.29) *fliH*	BB0294 (0.00) *flgB*	BB0668 (0.23) *flaA*	BB0772 (0.51) *flgI*	BB0772 (0.67) *flgI*	BB0051 (0.79) CHP (+)	BBE22 (0.44) *pncA* (-)
		Gene (Insertion Ratio) Gene Name											
		Percent Sites Positive											
T	2												
T	4												
C	2							ND					
C	4							ND					
Overall													
		Mean MFI Value											
T	2												
T	4												
C	2							ND					
C	4							ND					
Overall													

Percent Positive	81-100	61-80	41-60	21-40	0-20

Mean MFI	>1000	501-1000	100-500	<100

C

PEP-PTS

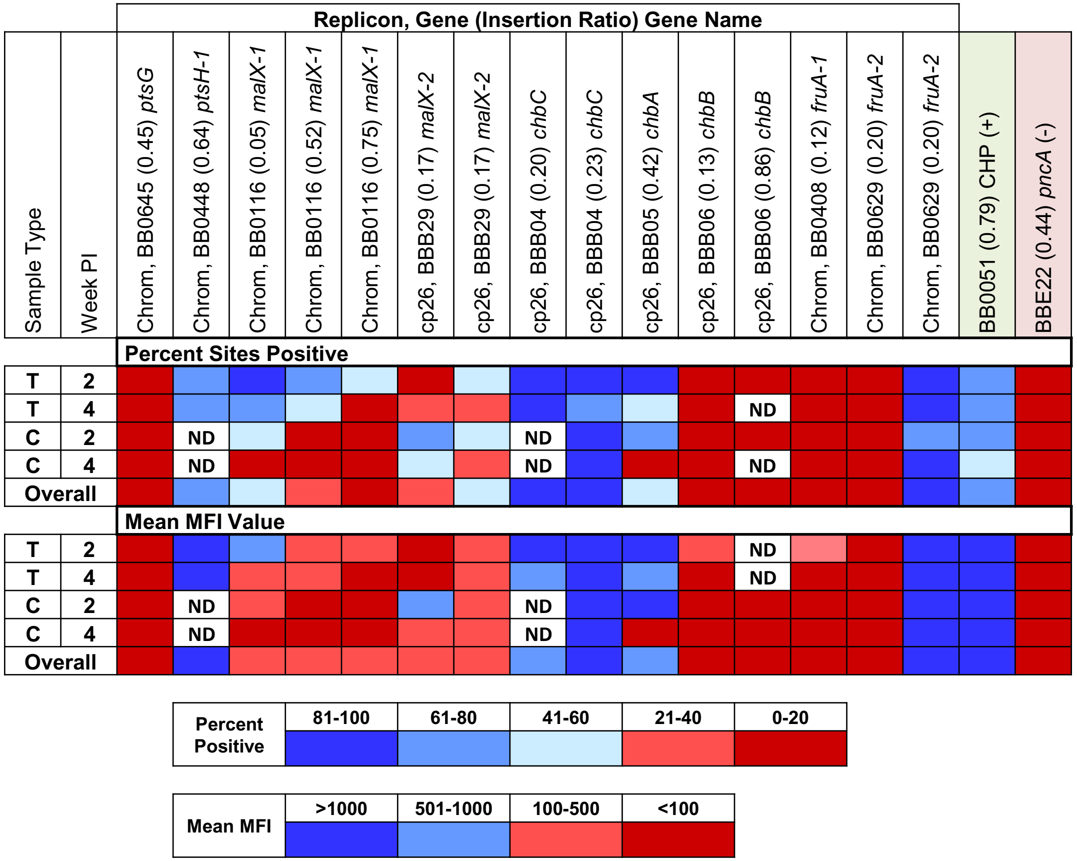
Replicon, Gene (Insertion Ratio) Gene Name
Sample Type
Week PI
Chrom, BB0645 (0.45) *ptsG*
Chrom, BB0448 (0.64) *ptsH-1*
Chrom, BB0116 (0.05) *malX-1*
Chrom, BB0116 (0.52) *malX-1*
Chrom, BB0116 (0.75) *malX-1*
cp26, BBB29 (0.17) *malX-2*
cp26, BBB29 (0.17) *malX-2*
cp26, BBB04 (0.20) *chbC*
cp26, BBB04 (0.23) *chbC*
cp26, BBB05 (0.42) *chbA*
cp26, BBB06 (0.13) *chbB*
cp26, BBB06 (0.86) *chbB*
Chrom, BB0408 (0.12) *fruA-1*
Chrom, BB0629 (0.20) *fruA-2*
Chrom, BB0629 (0.20) *fruA-2*
BB0051 (0.79) CHP (+)
BBE22 (0.44) *pncA* (-)
Percent Sites Positive
T 2
T 4
C 2
C 4
Overall
ND
Mean MFI Value
T 2
T 4
C 2
C 4
Overall
ND
Percent Positive
81-100
61-80
41-60
21-40
0-20
Mean MFI
>1000
501-1000
100-500
<100

D

Other Transport Systems

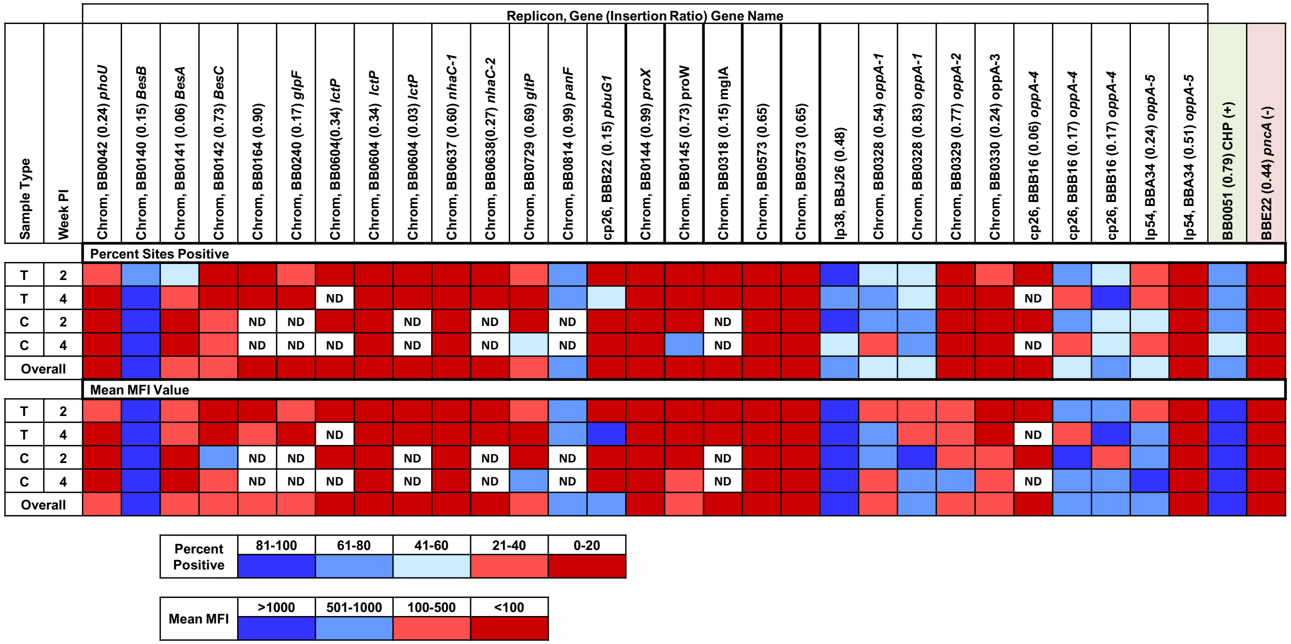

E

Plasmid Maintenance

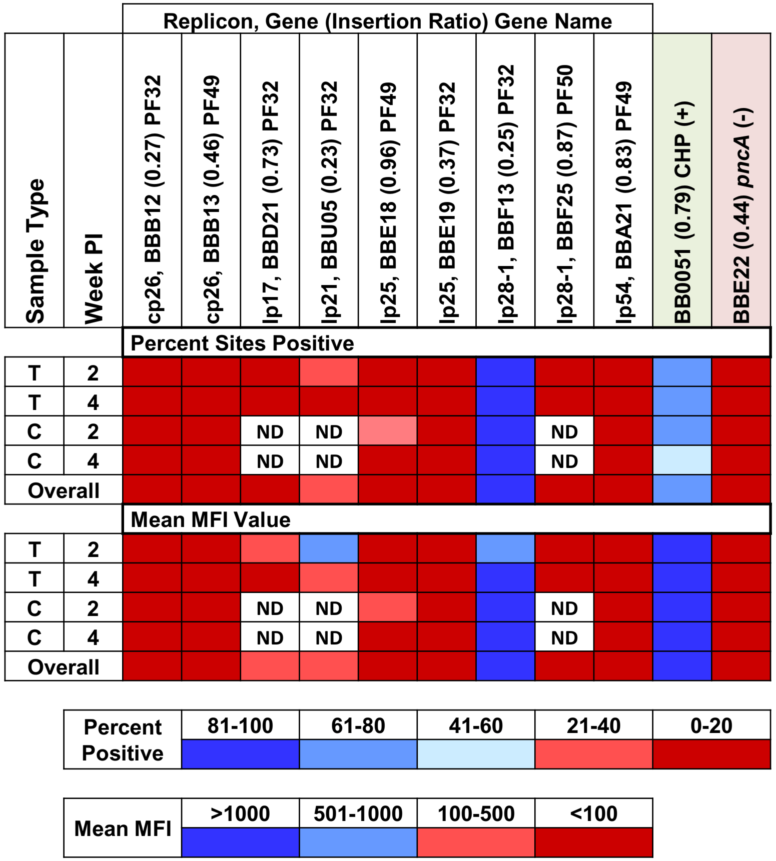

Supplement: File S1 — contains supporting information Tables 1 – 4 and Figures 1 –8. (PDF) [file pone.0047532.s001.pdf]
